# Synthesis and Light-Induced Actuation of Photo-Labile 2-Pyridyl-1,2,3-Triazole Ru(*bis*-bipyridyl) Appended Ferrocene Rotors

**DOI:** 10.3390/molecules23082037

**Published:** 2018-08-14

**Authors:** James A. Findlay, Jonathan E. Barnsley, Keith C. Gordon, James D. Crowley

**Affiliations:** Department of Chemistry, University of Otago, P.O. Box 56, Dunedin 9054, Otago, New Zealand; jono.barnsley@postgrad.otago.ac.nz (J.E.B.); keith.gordon@otago.ac.nz (K.C.G.)

**Keywords:** photochemical ligand-ejection, ruthenium(II) photo-ejection, light-induced molecular motion, molecular switches, nanotechnology

## Abstract

To realise useful control over molecular motion in the future an extensive toolbox of both actionable molecules and stimuli-responsive units must be developed. Previously, our laboratory has reported 1,1′-disubstituted ferrocene (Fc) rotor units which assume a contracted/π-stacked conformation until complexation of cationic metal ions causes rotation about the Ferrocene (Fc) molecular ‘ball-bearing’. Herein, we explore the potential of using the photochemical ejection of [Ru(2,2′-bipyridyl)_2_]^2+^ units as a stimulus for the rotational contraction of new ferrocene rotor units. Fc rotors with both ‘regular’ and ‘inverse’ 2-pyridyl-1,2,3-triazole binding pockets and their corresponding [Ru(2,2′-bipyridyl)_2_]^2+^ complexes were synthesised. The rotors and complexes were characterised using nuclear magnetic resonance (NMR) and ultraviolet (UV)-visible spectroscopies, Electro-Spray Ionisation Mass Spectrometry (ESI–MS), and electrochemistry. The 1,1′-disubstituted Fc ligands were shown to π-stack both in solution and solid state. Density Functional Theory (DFT) calculations (CAM-B3LYP/6-31G(d)) support the notion that complexation to [Ru(2,2′-bipyridyl)_2_]^2+^ caused a rotation from the *syn*- to the *anti*-conformation. Upon photo-irradiation with UV light (254 nm), photo-ejection of the [Ru(2,2′-bipyridyl)_2_(CH_3_CN)_2_]^2+^ units in acetonitrile was observed. The re-complexation of the [Ru(2,2′-bipyridyl)_2_]^2+^ units could be achieved using acetone as the reaction solvent. However, the process was exceedingly slowly. Additionally, the Fc ligands slowly decomposed when exposed to UV irradiation meaning that only one extension and contraction cycle could be completed.

## 1. Introduction

Exploring stimuli controlled motion in synthetic molecules will lead to advances in nanotechnology, and as such, molecular switches and machines have received increasing attention over recent years [1,2,3,4,5,6]. Relatively simple components, such as reversibly extendable/contractible units, using a stimulus as ubiquitous as light, could form useful components of larger assemblies. Many classes of molecular switches and machines have been realised, with molecular motion induced by a range of stimuli including chemical [7,8,9], electrochemical [10,11,12], and photochemical inputs [13,14,15]. Light-induced processes are particularly attractive as the input may be considered a clean energy source with excellent spatial resolution, and as such, photoactivated switching has been a focus of many research groups [16,17,18,19,20,21]. Some of the most notable examples of this subclass of molecular machines are those of Feringa and co-workers. They have developed a series of overcrowded alkenes which undergo unidirectional rotation through consecutive photo-induced isomerisation and thermal relaxation processes [22,23,24,25]. Examples of light-induced linear and rotational motion in rotaxanes and catenanes have been reported [26]. The photo-activated motion within these mechanically interlocked molecules, (MIMs), uses either the photoisomerisation of molecular recognition sites [27,28] or photo-induced redox processes (often through the use of a photosensitiser) to alter the preferred interaction between a macrocycle and the molecular fragment from which it is mechanically inseparable. The majority of the systems developed to date are built from mainly organic components. However, there are a few in which photoactive metals complexes are exploited to generate the molecular motion [29]. Of note is work of Sauvage and co-workers who have reported copper(I)/copper(II) photo-oxidation switches [30,31], but more relevantly have also generated a significant amount of work around photo-induced ligand ejection from ruthenium(II) ions [32,33,34,35].

The most commonly investigated metal ion for photo-induced ligand ejection is Ru(II), although examples involving osmium(II) complexes have been reported [36]. Photo-induced ligand-ejection from ruthenium(II), which is usually considered to form inert coordination bonds, has been recently studied for medicinal applications, with a number of reports developing prodrugs which undergo photo-ligand ejection, towards ‘switch-on cytotoxicity’ type technology to fight diseases such as cancer [37,38,39,40,41,42]. However, other potential applications have been investigated [43], including photo-induced self-assembly [44], and molecular motion [45]. There are two main strategies used when designing bidentate ligands for the purpose of photo-ejection from [Ru(L)_2_]^2+^ (where L is a bidentate N–N donor ligand) type units: One is to create a sterically encumbered coordination environment, often by adding functionalities in the *ortho* positions of coordinating pyridyl donors [32,40,46,47]. The other is to simply create a ligand with weaker donor capability [48]. It has been shown bidentate ligands of the 2-pyridyl-1,2,3-triazole (pytri) or the bis-triazolyl type are suitable for this purpose as 1,2,3-triazole rings possess a weaker donor ability than pyridine [49,50,51].

While there are reports of materials, such as nanoparticles [52] and polymers [53,54,55], able to undergo expansion/contraction type motion upon application of a light stimulus, the literature is lacking in photo-induced expansion/contraction type mechanical motions of discrete molecules. Ferrocene (Fc) has been exploited as a molecular ‘ball-bearing’, because the two cyclopentadienyl (cp) rings are able to rotate about the iron centre. One can envisage substituents of the cp rings of an Fc unit being reversibly brought together and repelled through use of one or more stimuli. There are examples of rotatable ferrocene switches controlled by hydrogen bonding [56,57], chloride ions [58], metal ion coordination [59], and electrochemistry [60]. Previously our lab reported [61,62] the electrochemically driven expansion and contraction of Fc based molecular actuators/‘folding rulers’. The switching mechanism used in both of these systems exploits the switching system developed by Sauvage and Schmittel and co-workers [31,63,64,65,66]. Both ferrocene rotors bear 2,2′-bipyridine chelators able to bind Cu(I) ions in heteroleptic tetrahedral complexes with bulky 6,6′-dimesityl-2,2′-bipyridine ligands. This forms the extended state of the Fc rotors as the bulk of the co-ligands require rotation about the Fc units. Oxidation to Cu(II) alters the coordination tendency of the metal ion such that it de-complexes from the Fc rotors to bind to terpy ligands present in solution. This allows the Fc rotors to re-establish their native π–π stacked contracted conformations.

**Figure 1:**
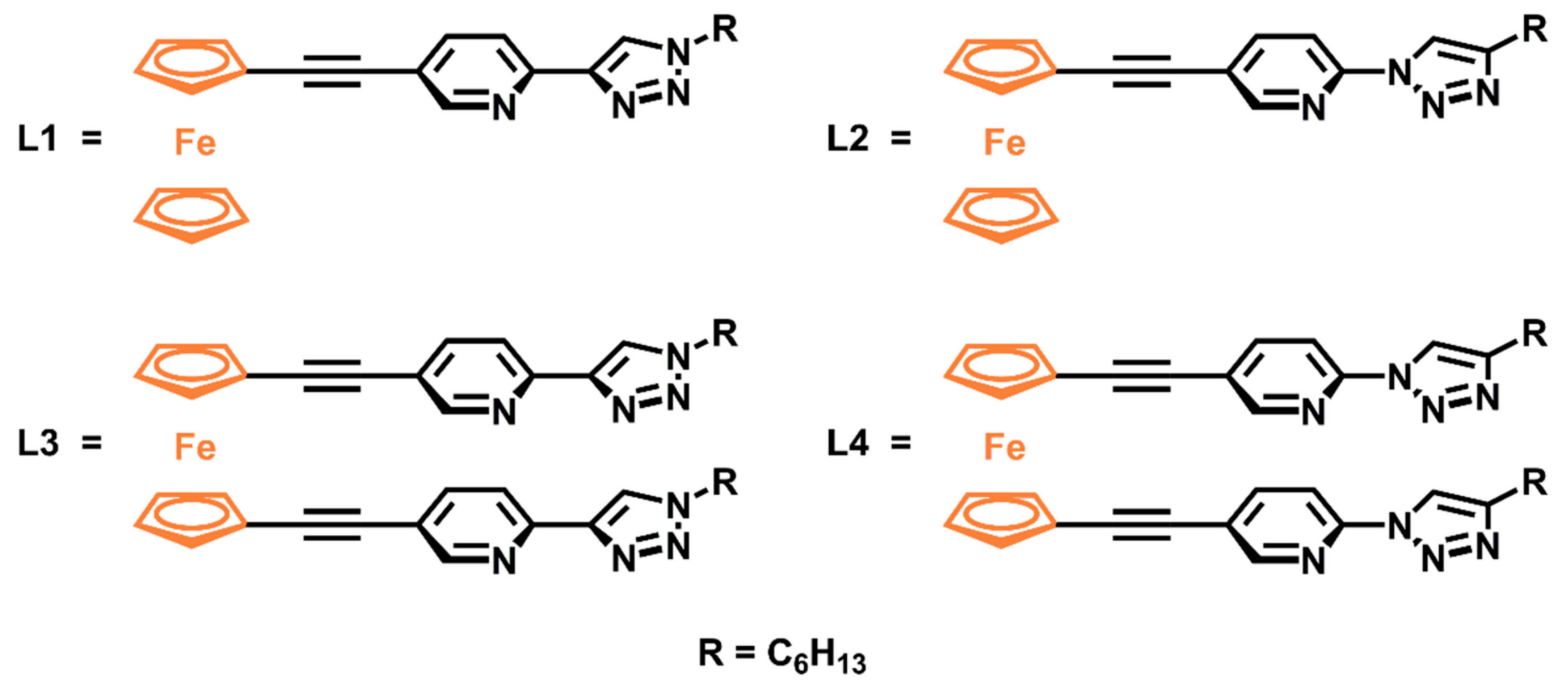
The molecular structures of ferrocene model (**L1**–**L2**) and rotor ligands (**L3**–**L4**).

Herein, we explore the potential of *bis*-(2,2′-bipyridyl)-ruthenium(II) ([Ru(bipy)_2_]^2+^) units as a stimulus to cause extension within new *bis*-bidentate pytri Fc ligands from their native *syn* π–π stacked conformational state, and the reverse process of photo-ejection of the [Ru(bipy)_2_]^2+^ units. Two different structural isomers of bidentate pytri binding pockets, namely ‘regular (*reg*)’ and ‘inverse (*inv*)’ pytri ligands were decided upon as likely photo-labile candidates as we have previously shown that [Ru(bipy)(*inv*-pytri)]^2+^ complexes would photo-eject the *inv*-pytri ligands when exposed to UV irradiation in acetonitrile [49]. The ‘inverse’ pytri structural isomers are weaker chelators than their ‘regular’ analogues as the nitrogen donor in the 2-position of the 1,2,3-triazole is less electron rich [49]. We have synthesised two new Fc containing *inv*-pytri ligands (**L2** and **L4**) to form a small family with two *reg*-pytri analogues (**L1** and **L3**, Figure 1), previously reported by us [67]. The corresponding model [Ru(pytri)(bipy)_2_](BF_4_)_2_ complexes **L1Ru** and **L2Ru** were generated from the model ligands **L1** and **L2**. The extended tetra-cationic rotor complexes **L3Ru** and **L4Ru** were then generated from the Fc rotor ligands **L3** and **L4**. The four Ru(II) complexes were characterised using nuclear magnetic resonance (NMR), infrared (IR), and ultraviolet (UV)-visible absorption spectroscopies, along with electro-spray ionisation mass spectrometry (ESI–MS) and electrochemical investigations. Additionally, irradiation of the four Ru(II) complexes in acetonitrile solution with UV-light (254 nm) was shown to liberate [Ru(bipy)_2_(CD_3_CN)_2_](BF_4_)_2_, as shown through ^1^H NMR spectroscopy and ESI–MS. Thermal re-coordination of the Ru(II) fragment(s) to complete the switching cycle was then attempted. However, the reverse process could not be completed because the Fc ligand decomposed during the photo-ejection process.

## 2. Results

### 2.1. Synthesis of Ligands and Ru(II)(bipy)_2_ Complexes

**Figure 2:**
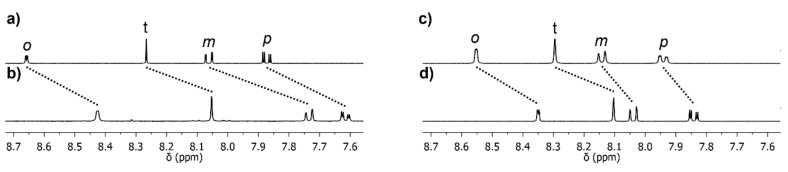
Partial stacked ^1^H nuclear magnetic resonance (NMR) spectra (400 MHz, 298 K) of: (**a**) **L1**, and (**b**) **L3** (CD_3_CN), and (**c**) **L2**, and (**d**) **L4** (CDCl_3_); labels refer to *ortho*, *meta*, and *para* pyridyl and the triazole, t, signals of the ligands.

Ligands **L1**−**L4** (Figure 1) were all synthesised using Pd(0)-catalysed Sonogashira cross coupling and Cu(I) catalysed azide-alkyne cyclo addition (CuACC) protocols. The syntheses of new ligands **L2** and **L4** are shown in the Supplementary Material (Schemes S1 and S2), along with full experimental procedures for all previously unreported intermediates. **L1**−**L4** were characterised by ^1^H NMR spectroscopy, ^13^C NMR spectroscopy, ESI–mass spectrometry, elemental analysis, UV-visible spectroscopy, and in the cases of **L2** and **L4**, X-ray crystallography (see Supplementary Material). Comparision of the ^1^H NMR spectra of the model ligand with the rotor systems indicated that the Fc rotor based switches (**L3** and **L4**) adopt a π–π stacked *syn*-conformation in solution. As shown in Figure 2, the ^1^H NMR signals of the aromatic binding pockets of the disubstituted Fc ligands are shifted up-field relative to the corresponding signals in the model ligands, indicative of π–π interactions.

**Figure 3:**
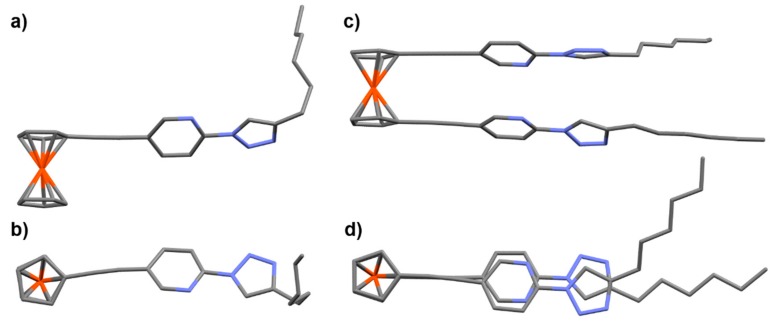
Depiction of the solid state structures of **L2** and **L4**; (**a**) **L2** side view, (**b**) **L2** top view, (**c**) **L4** side view, (**d**) **L4** top view. Hydrogen atoms omitted for clarity.

The solid-state structures of **L2** and **L4** (Figure 3) show in both cases the Fc units exhibit the expected eclipsed conformation of the cyclopentadienyl rings. The aromatic pytri binding pockets of **L4** are also π–π stacked (pyridine–pyridine centroid distance = 3.58 Å, tri–tri centroid distance 3.913 Å and the angle between the ferrocene substituents = 6.58°, (see Supplementary Material, Figure S53), providing further support for the contracted *syn* conformation of the disubstituted Fc ligands in their native state.

**Figure 4:**
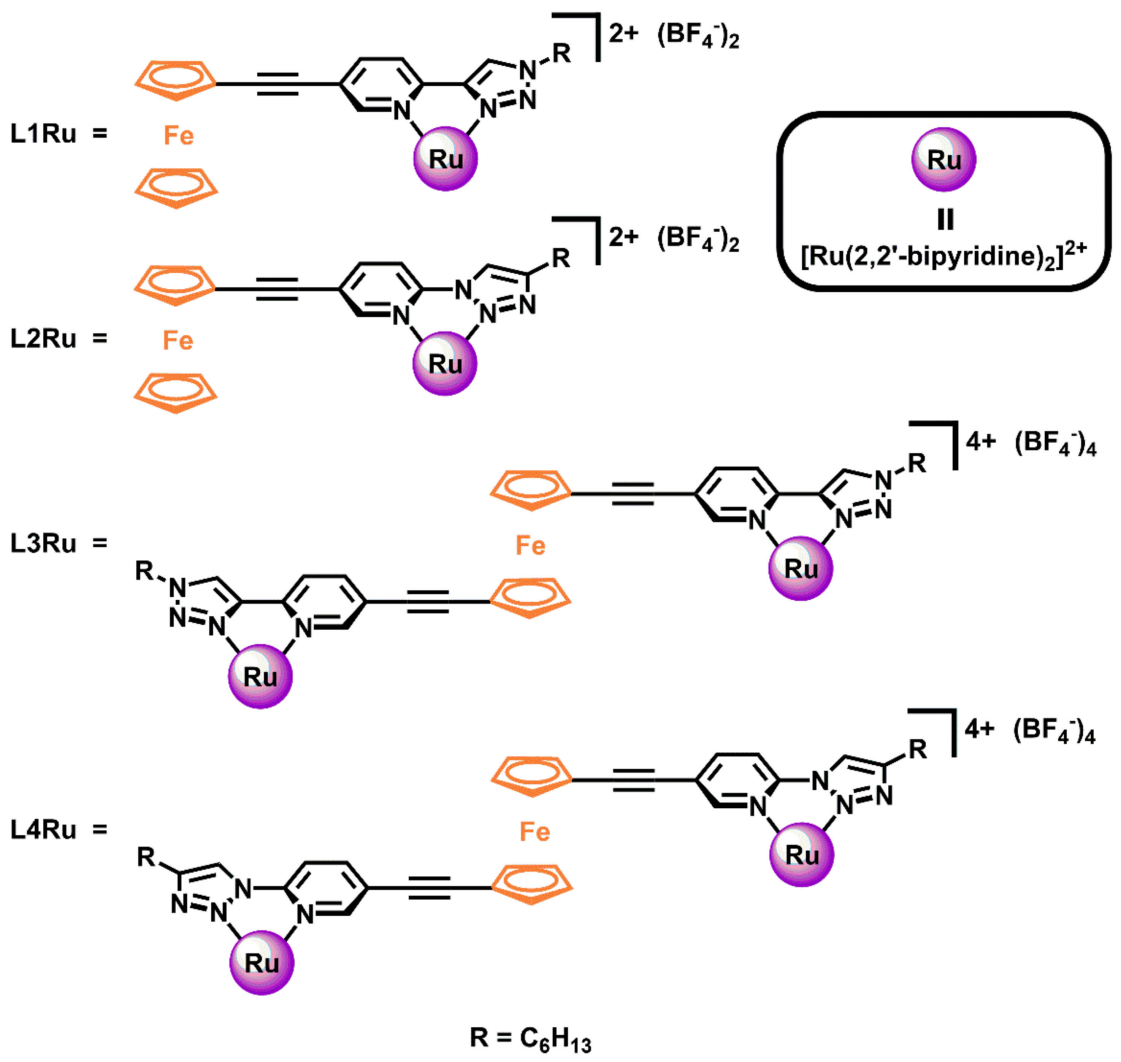
The molecular structures of the ruthenium complexes **L1Ru**–**L4Ru**.

The synthesis of the [Ru(bipy)_2_]^2+^ complexes of each of **L1**−**L4** (Figure 4) was achieved by first generating [Ru(bipy)_2_(acetone)_2_](BF_4_)_2_ by stirring [Ru(bipy)_2_(Cl)_2_] and two equivalents of AgBF_4_ in acetone. Refluxing this Ru(II) reactive intermediate with each of **L1**−**L4** in acetone gave the desired di- (**L1**−**L2**) and tetra-cationic (**L3**−**L4**) complexes in good yields (74–94%). **L1Ru**−**L4Ru** were all characterised by ^1^H NMR spectroscopy, ESI–mass spectrometry, elemental analysis and UV-visible spectroscopy. The ^1^H NMR spectra of **L1Ru**−**L4Ru** all showed downfield shifts of the ligand signals, most notably the large downfield shift of the triazole signal in all cases, indicative of complexation to Ru(II) (Supplementary Material). Furthermore, the two symmetric Fc cyclopentadienyl signals in the ^1^H NMR of both **L3** and **L4** are replaced by a more complicated set of signals in the ^1^H NMR of **L3Ru** and **L4Ru**, presumably due to the different isomeric mixtures expected with respect to the Ru(II) centres (Δ/Δ, Δ/Λ, and Λ,Λ). Diffusion-ordered ^1^H NMR spectroscopy (DOSY NMR) was used to further confirm the numerous signals in the aromatic regions of the ^1^H NMR spectra of **L1Ru**−**L4Ru** were, indeed, diffusing at the same rate, thus, belonging to species of the same molecular weight (Supplementary Material). Interestingly the aromatic signals in the ^1^H NMR spectrum of **L3Ru** suggested the presence of two isomers (or two NMR equivalent pairs of isomers) in equal proportion, however, the same was not observed in the spectrum of **L4Ru** which displays a fully symmetric spectrum. Despite the potential for different isomeric mixtures (Δ/Δ, Δ/Δ, and Λ,Λ), the DOSY and mass spectral data support only one molecular weight being present in each case (Supplementary Material).

**Figure 5:**
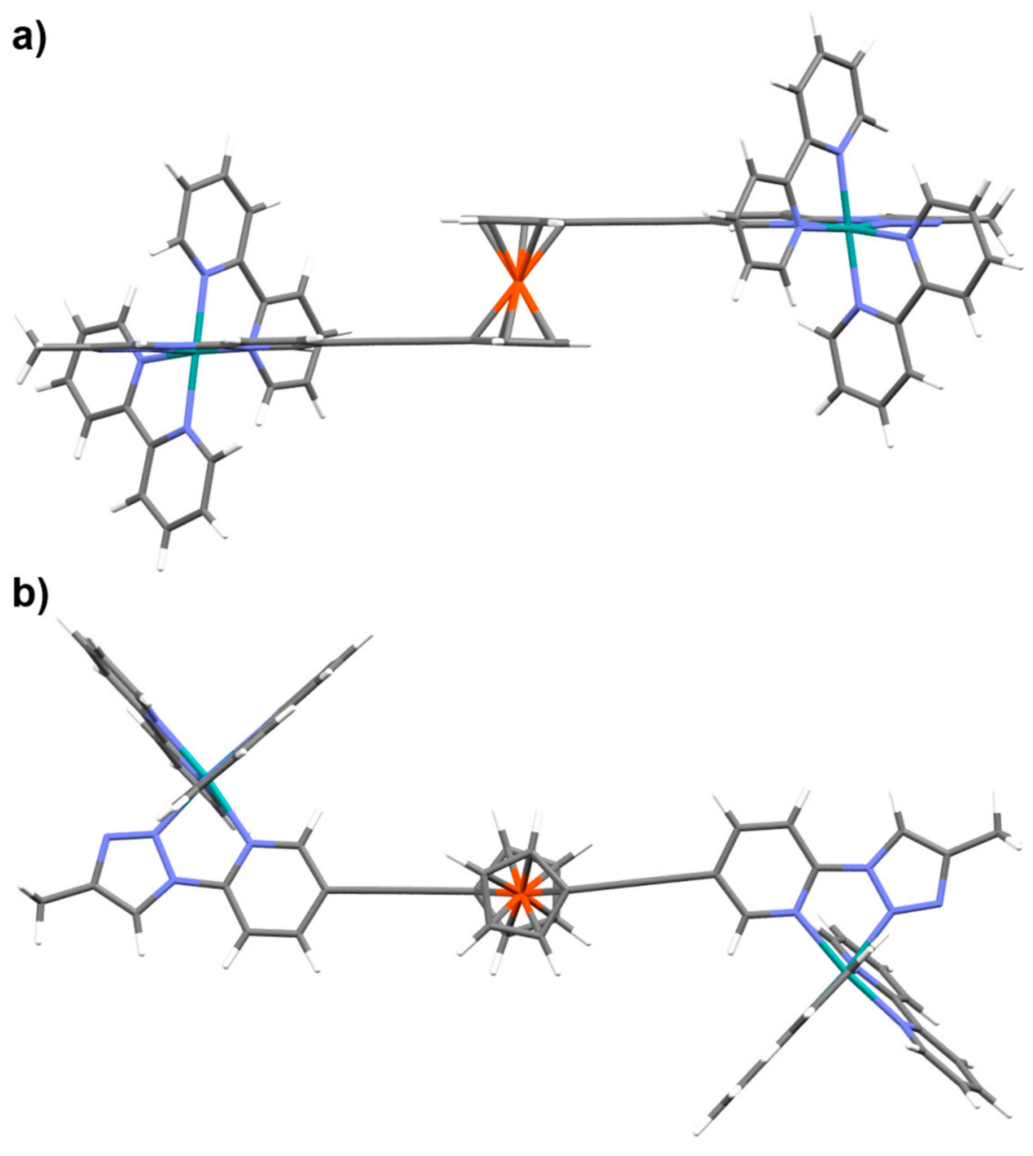
Density Functional Theory (DFT) (CAM-B3LYP, 6-31G(d)) optimised structure of **L4Ru** (dihedral ≈ 180°) with (**a**) side view and (**b**) top view.

Density Functional Theory (DFT) calculations (see the Supplementary Material for the details of the computational methods) were performed to support the notion the rotor complexes **L3Ru** and **L4Ru** must adopt an extended *anti*-conformation. The optimised open structure (dihedral angle ≈ ±180°) was observed to be the lowest energy conformation for both complexes (Figure 5 and Supplementary Material, Figures S55−S62). As the dihedral angle is decreased, the relative energy increases (see Figure S54). In the ranges of ±180° to ±110° the relative energy is within 5 kJ mol^−1^, and according to the Boltzmann distribution, will be accessible at room temperature. Dihedral angles of ±80° (>7 kJ mol^−1^) or higher become energetically disfavoured, with populations of less than 6%, according to the Boltzmann distribution. This energy scan indicates that the ±180° to ±110° range will be most vastly populated, and smaller dihedral angles will be disfavoured. Thus, when the Fc rotors (**L3** and **L4**) are uncomplexed, a *syn*-stacked conformation is preferred. However, complexation to the [Ru(bipy)_2_]^2+^ units causes a switch to an *anti*-extended conformation presumably the switching is driven by a combination of charge repulsion between the Ru(II) ion and destabilising steric interactions.

### 2.2. UV-Visible Spectroscopy

**Figure 6:**
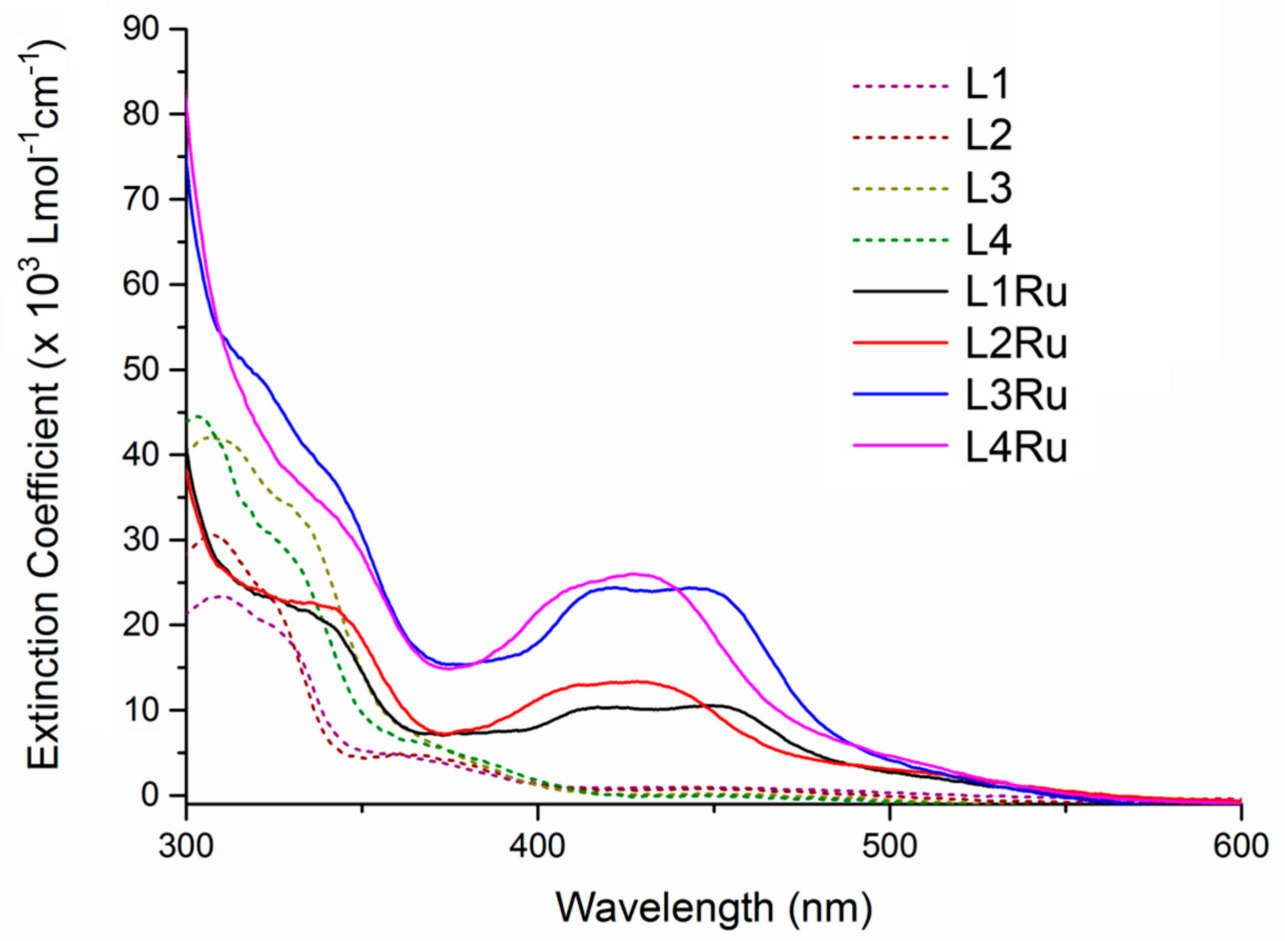
Overlaid UV-Visible spectra of **L1–L4** and **L1Ru−L4Ru** recorded in dichloromethane solution (~10^−5^ molL^−1^, 298 K).

The UV-Visible spectra of all ligands (**L1**−**L4**) and complexes (**L1Ru**−**L4Ru**) were recorded in dichloromethane (DCM) solution (Figure 6 and Supplementary Material, Figures S38 and S39). In the electronic absorption spectra of the ligands, the features of note are the π–π* transitions around 310 and 370 nm, and the transition at around 450 nm which is assigned as an Fc based process [68,69]. In the spectra of the complexes, convoluted absorbance features between 400 and 470 nm are observed (along with shoulder features at around 500 nm) and are assigned as Ru based Metal-to-Ligand Charge Transfer (MLCT) transitions. For **L1Ru** and **L2Ru,** the extinction coefficient(s) for these transitions are ~10 and ~13 × 10^3^ L mol^−1^ cm^−1^ (at 422 and 428 nm) respectively. Likewise, for **L3Ru** and **L4Ru**, extinction coefficients of ~24 and ~26 × 10^3^ L mol^−1^ cm^−1^ (at 422 and 427 nm) are observed respectively. The ratio of Ru(II) ions to Fc units (1:1 for **L1Ru** and **L2Ru**, and 2:1 for **L3Ru** and **L4Ru**) correlates with the approximate two-fold increase in the intensity of the transition(s) assigned to ruthenium-based MLCT transitions. The UV-Visible absorption spectra of **L1Ru**−**L4Ru** were also recorded in acetonitrile (see Supplementary Material, Figure S40) and differ little to the spectra recorded in DCM.

### 2.3. Electrochemistry

Cyclic and Differential Pulse Voltammetry (CV and DPV) experiments were carried out on 1 mM dichloromethane (DCM) solutions of **L1**−**L4** and the corresponding ruthenium complexes **(L1Ru**−**L4Ru)**, with 0.1 M Bu_4_NPF_6_ as supporting electrolyte. Cyclic voltammograms for all eight species, with inset DPV plots, can be found in the Supplementary Material (Figures S30–S37), and the half cell potential (E°) for all observed processes in Table 1 below. The current and potential observed for all processes were reproducible over multiple cycles. **L1**−**4** displayed the expected Fc^+^/Fc couple between 0.62 and 0.75 V. The two ferrocenyl oxidation potentials for the mono-substituted ligands are slightly lower than those displayed by the di-substituted ligands (Table 1). All four of **L1Ru**−**L4Ru** displayed the Fc^+^/Fc couple along with a Ru^III/II^ couple and two reduction processes assigned to the two bipy ligands on each Ru(II) centre. As displayed by the ligands, the ferrocenyl based oxidation process occurs at a lower potential in the mono-nuclear complexes versus the di-nuclear analogues. As expected, the current response for the Ru^III/II^ couple is larger for **L3Ru** and **L4Ru** versus **L1Ru** and **L2Ru**, consistent with the molecular formulae. The ruthenium based oxidation process occurs at a higher potential in the ‘inverse’ pytri complexes (**L2Ru** and **L4Ru**) than in the ‘regular’ pytri analogues, similar to previously reported related compounds [49]. Furthermore, both bipy reduction processes are also shifted to the anodic potential in the ‘inverse’ analogues, relative to the ‘regular’. Reduction processes for the pytri ligands were not observed under the conditions employed here within the solvent window.

### 2.4. Photo-Reactivity of L1Ru−L4Ru

UV irradiation (254 nm) of [D3]acetonitrile solutions (2 mM) of each of **L1Ru**−**L4Ru** resulted in a photochemical reaction in all cases. At least one new set of signals appeared in the ^1^H NMR of UV-irradiated samples, which increased in signal height relative to the initial spectrum over time (see Supplementary Material, Figures S41–S44). In all four cases, the major species being produced was confirmed to be [Ru(bipy)_2_(CD_3_CN)_2_]^2+^ through direct comparison with the ^1^H NMR (CD_3_CN) of an independently synthesised sample of [Ru(bipy)_2_(CH_3_CN)](BF_4_)_2_. This was also confirmed using ESI–MS. Interestingly no signals corresponding to the liberated free ligands were observed in any case. Irradiation of **L1Ru**−**L4Ru** under identical conditions in [D6]acetone resulted in no photo-reaction, suggesting a more coordinating solvent (or other ligand) was required for photo-ejection of **L1**−**4** to proceed.

**Figure 7:**
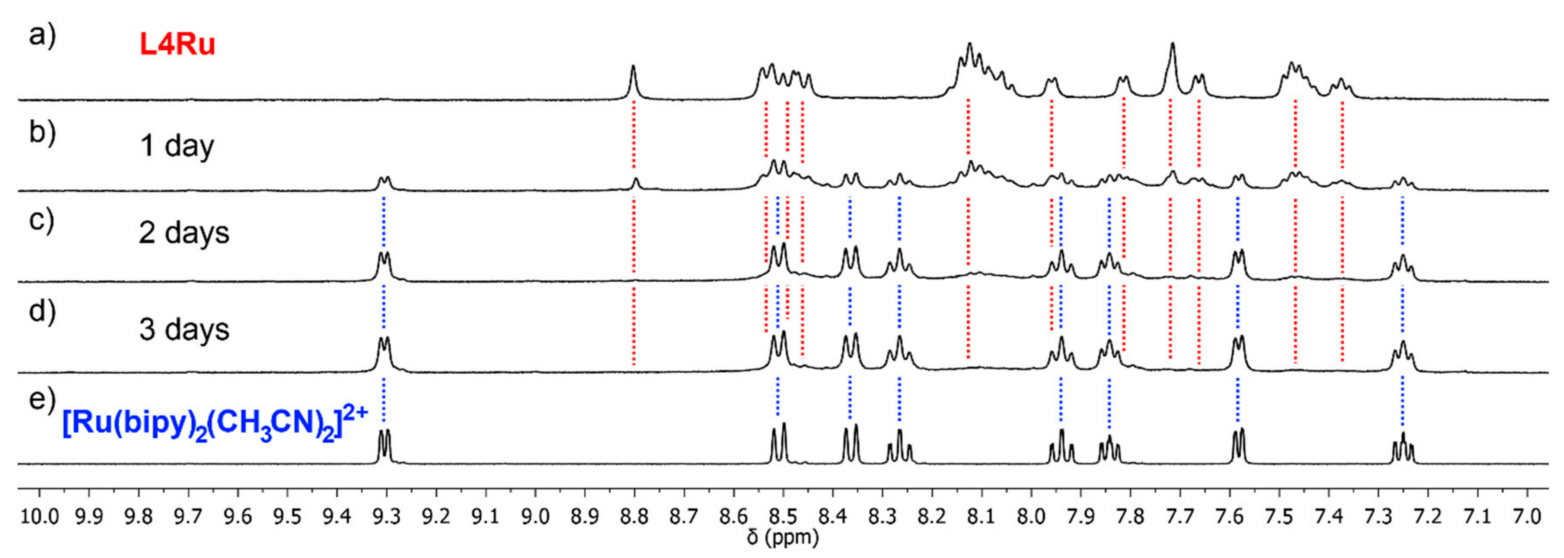
Partial stacked ^1^H NMR spectra (400 MHz, CD_3_CN, 298 K), showing the photo-ejection of [Ru(bipy)_2_(CD_3_CN)_2_]^2+^ (blue dashed lines) from L4Ru (red dashed lines) over time under UV (254 nm) irradiation; (**a**) ^1^H NMR of unirradiated **L4Ru**, (**b**–**d**) ^1^H NMR of **L4Ru** after 1–3 days of UV irradiation, and (**e**) ^1^H NMR of independently synthesised [Ru(bipy)_2_(CH_3_CN)_2_](BF_4_)_2_. Note: **L4** is insoluble in acetonitrile, hence, no spectrum of a free ligand for comparison.

Complexes **L2Ru** and **L4Ru** showed full dissociation of the [Ru(bipy)_2_(CD_3_CN)_2_]^2+^ units. No signals of significant intensity were observed in either case which might indicate the formation of intermediate species (for instance, in the case of **L4Ru**, where one [Ru(bipy)_2_(CD_3_CN)_2_]^2+^ unit had dissociated but the other had not yet). Interestingly, **L2Ru** and **L4Ru** underwent ligand ejection at different rates. Greater than 95% of **L2Ru** ([D3]acetonitrile solution) was converted to [Ru(bipy)_2_(CD_3_CN)_2_]^2+^ after 7 days of irradiation, while **L4Ru** took less than 3 days under identical conditions (Figure 7). The explanation as to why **L4Ru** reaches a fully ejected state in less than half the time taken for the same process in **L2Ru** is not entirely clear, but we suggest two plausible reasons: **L4Ru** was able to absorb more photons in the appropriate energy range of the light source used, per Ru(II) centre, or perhaps once one Ru(II) centre is lost from **L4Ru**, the free pytri binding pocket may have become involved in the second ejection process.

**Scheme 1:**
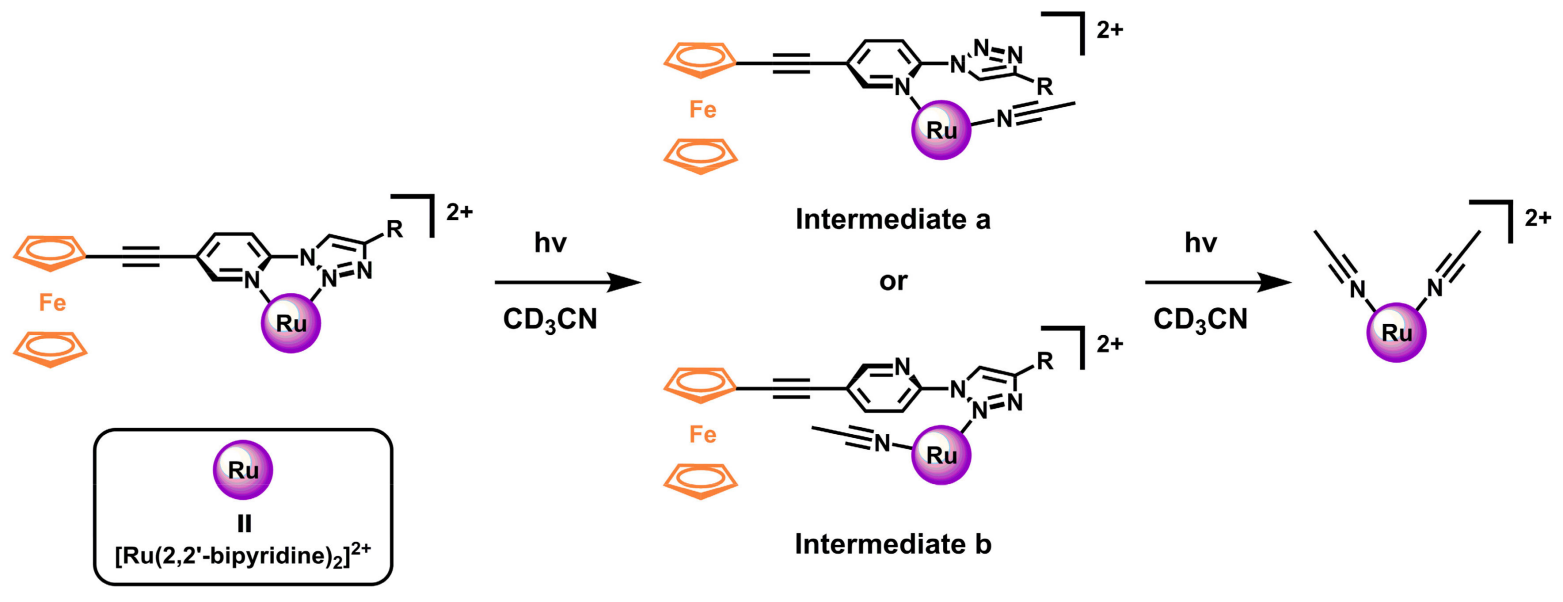
Representative example of the formation of intermediate species, where one of either the pyridine or triazole of the 2-pyridyl-1,2,3-triazole is photo-ejected initially giving rise to either intermediate a or b before full ligand dissociation is achieved at a later time.

The behaviour of the *reg*-pytri analogues was significantly different to that of the *inv*-analogues. After 26 days of irradiation in [D3]acetonitrile solution, **L1Ru** had undergone approximately 75% conversion to the photoreaction products (based on the integration of key ^1^H NMR signals). Signals that did not correspond to the starting Ru complex, nor [Ru(bipy)_2_(CD_3_CN)_2_]^2+^ or the free ligand began to grow at the beginning of the irradiation experiment. These signals were presumed to be intermediate ligand ejection products (Scheme 1 below), where either the 1,2,3-triazole (intermediate a) or the pyridine (intermediate b) of the ferrocene ligand had been replaced by an acetonitrile solvent molecule, but not the other. This is consistent with the observation of at least two sets of these unassigned signals having similar chemical shifts and multiplicity appearing and is also consistent with previously reported photo ejection experiments of pytri ligands [49].

Interestingly, after the same length of time (26 days), **L3Ru** had reached a point where essentially none of the original signals of the intact **L3Ru** species were present. However, full conversion to [Ru(bipy)_2_(CD_3_CN)_2_]^2+^ had also not occurred, as a significant amount of the material appeared to be in an intermediate stage. When analogous [Ru(bipy)_2_(*reg-*Bnpytri)]^2+^ complexes (where *reg*-Bnpytri = 2-(1-benyzl-1H-1,2,3-triazol-4-yl)pyridine), which were not substituted with Fc, were irradiated (254 nm, [D3]acetonitrile), no photo-reaction was observed [49]. We propose a different absorption profile due to the substituents of the pytri ligands presented here as the likely cause of this difference.

With the photochemical behaviour of the four ruthenium complexes under UV irradiation determined, we turned our attention to the reverse process of thermal re-coordination. Our hypothesis was heating the [D3]acetonitrile solution of photo-reaction products would affect re-coordination of the Fc ligands and re-extended the rotors, thus rendering the system(s) reversible. However, heating the [D3]acetonitrile solutions of fully photo-ejected samples of **L2Ru** and **L4Ru** at 80 °C in the dark, resulted in almost no change to the ^1^H NMR of the mixtures within 7 days, and certainly no return of the signals corresponding to the original complexes. Samples of **L1Ru** and **L3Ru** in [D3]acetonitrile which had been irradiated for 26 days were also heated to 80 °C in the dark. After 48 h no change to the ^1^H NMR was observed. We then took completely photo-ejected samples of **L2Ru** and **L4Ru** that had not been heated and evaporated the [D3]acetonitrile solvent under reduced pressure to dryness. Re-dissolving the photo-reaction product residue in [D6]acetone and heating at 65 °C in the dark produced no reaction also.

The lack of re-coordination and the absence of free ligand signals in the NMR spectra of the photo-irradiated samples suggested that the Fc ligands may be decomposing under the conditions of the experiments. To test this postulate control experiments were carried out on fresh separate samples of **L1** and **L2**. Samples of **L1** and **L2** in [D3]acetonitrile were irradiated under the same conditions as the complexes and were monitored by ^1^H NMR (see Supplementary Material Figures S49–S50). Within one day the orange colour of the solution had lessened, and brown precipitate was being generated, confirming the UV-promoted degradation of the ligands.

Additionally, we attempted to react each of the model ligands **L1** and **L2** with one equivalent of independently synthesised [Ru(bipy)_2_(CH_3_CN)_2_](BF_4_)_2_ to generate **L1Ru** and **L2Ru** from the two components that should be present in the photo-reaction product mixtures. Combining [Ru(bipy)_2_(CH_3_CN)_2_](BF_4_)_2_ and either **L1** or **L2** in [D3]acetonitrile and heating at 80 °C in the dark for 24 h resulted in the replacement of the CH_3_CN ligands with [D3]acetonitrile, as evidenced by ^1^H NMR. However, none of **L1Ru** or **L2Ru** was generated (see Supplementary Material, Figures S45 and S46). The sample of these two mixtures after 24 h of heating had also produced some black precipitate; presumably degraded Fc ligand, generated due to prolonged heating in the coordinating [D3]acetonitrile solvent. This was corroborated by the decrease in intensity of the signals of the free ligands relative to the [Ru(bipy)_2_(CH_3_CN)_2_](BF_4_)_2_ signals in the ^1^H NMR. Separate, pure, samples of **L1** and **L2** were heated to 80 °C in [D3]acetonitrile, and within 24 h a significant amount of brown precipitate had formed confirming the Fc ligands degrade when heated in acetonitrile solution. The thermal coordination experiments between each model ligand and [Ru(bipy)_2_(CH_3_CN)_2_](BF_4_)_2_ were then repeated in [D6]acetone solution (see Supplementary Material, Figures S47 and S48). Interestingly, [D6]acetone molecules had begun replacing the coordinated acetonitrile of [Ru(bipy)_2_(CH_3_CN)_2_](BF_4_)_2_ at room temperature, in the time required to collect a ^1^H NMR. Upon heating at 65 °C in the dark for 4 h, signals which corresponded to those of **L1Ru** and **L2Ru** had appeared in the respective mixtures. The proportion of **L1Ru** and **L2Ru** grew overtime with continued heating. After 12 days the ^1^H NMR of the mixture of **L1** and [Ru(bipy)_2_(CH_3_CN)_2_](BF_4_)_2_ was almost identical to that of **L1Ru**. The equivalent process was observed for **L2**, however at a lower rate. This showed that the thermal re-coordination of at least **L1** or **L2** to [Ru(bipy)_2_(CH_3_CN)_2_]^2+^ was, in fact, possible, but not from the mixture produced by UV-irradiation in [D3]acetonitrile, due to the decomposition of the ligands post Ru(II) photo-ejection.

The equivalent re-coordination experiments in [D6]acetone for **L3** and **L4** were not attempted, as the ejection from **L3** was extremely slow, and both have a very low solubility in acetone, making neither an ideal candidate for reversibly switchable rotors.

## 3. Conclusions

In summary, 2-pyridyl-1,2,3-triazole (pytri) ferrocene rotor ligands and the corresponding [Ru(bipy)_2_]^2+^ complexes were successfully synthesised and characterised. The ferrocene rotors adopt a *syn* π-stacked conformation. Complexation of the rotors to two [Ru(bipy)_2_]^2+^ units generates a conformational switch to an *anti*-extended state. UV irradiation experiments resulted in photo-labilisation of the Ru(II) units, and formation of [Ru(bipy)_2_(CH_3_CN)_2_]^2+^, concomitant with contraction of the ferrocene rotors to the *syn* π-stacked conformation. Both the ‘regular’ (**L3Ru**) and ‘inverse’ pytri rotor complexes (**L4Ru**) underwent photo-ejection in acetonitrile solution. However, as expected from earlier model studies the *inv*-system photo-ejected more rapidly (3–7 days) than the *reg*-complexes (approx. a month). While photo-ejection of the [Ru(bipy)_2_(CH_3_CN)_2_]^2+^ units completed an extension-and-contraction cycle, further reversible switching could not be achieved as the Fc ligands decomposed under the conditions of the UV-irradiation experiments.

While the current Fc switches are unstable under the photo-switching conditions, the results obtained herein suggest that ruthenium(II) based ligand photo-election reactions could be exploited to develop reversible photo-switches in correctly designed, more robust systems. Efforts towards new more rapid and robust ruthenium(II) based photo-switches based on other “click” 1,2,3-triazoles ligands are underway.

## 4. Materials and Methods

Unless otherwise stated, all reagents were purchased from commercial sources and used without further purification. 1,1′-diiodoferrocene was synthesised and purified according to a literature preparation [70]. The ligands **L1** and **L3** were synthesised using our previously reported methods [67]. Solvents were laboratory reagent grade. Substances (and their abbreviations) used herein include, methanol (MeOH), dichloromethane (DCM), tetrahydrofuran (THF), acetonitrile (CH_3_CN), ethylenediaminetetraacetate (EDTA), *tris*((1-benzyl-1*H*-1,2,3-triazol-4-yl)methyl)amine (TBTA). Petrol refers to the fraction of petroleum ether boiling in the range 40–60 °C. ^1^H and ^13^C NMR spectra were recorded on either a 400 MHz Varian 400 MR or Varian 500 MHz VNMRS spectrometer (Varian, Santa Clara, CA, USA). Chemical shifts are reported in parts per million and referenced to residual solvent peaks (CDCl_3_: ^1^H δ 7.26 ppm, ^13^C δ 77.16 ppm; CD_3_CN: ^1^H δ 1.94, ^13^C δ 1.32, 118.26 ppm, *d*_6_-acetone: ^1^H δ 2.05 ppm; ^13^C δ 29.84, 206.26 ppm). Coupling constants (*J*) are reported in Hertz (Hz). Standard abbreviations indicating multiplicity were used as follows: m = multiplet, q = quartet, t = triplet, dt = double triplet, d = doublet, dd = double doublet, ddd = double double doublet, s = singlet. Infrared spectra were recorded on a Bruker ALPHA FT-IR spectrometer with an attached ALPHA-P Attenuated Total-internal Reflection (ATR) measurement module (Bruker Daltonik, Bremen, Germany). Microanalyses were performed at the Campbell Microanalytical Laboratory at the University of Otago. Electrospray ionisation mass spectra (ESI–MS) were collected on a Bruker micro-TOF-Q spectrometer (Bruker Daltonic, Bremen, Germany). UV-visible absorption spectra were acquired with a Shimadzu UV-2600 UV-Vis spectrophotometer (Shimadzu, Kyoto, Japan). A CEM S-class microwave reactor (CEM corporation, Mathews, NC, USA) was used to carry out microwave enhanced reactions.

**Safety Note:** Whilst no problems were encountered during the course of this work, azide compounds are potentially explosive, and appropriate precautions should be taken when working with them.

**Synthesis of L2:** To degassed toluene (5 mL) was added 2-(6-azido-3-pyridinyl)ethynylferrocene (220 mg, 4.95 mmol), [Cu(CH_3_CN)_4_](PF_6_) (0.369 mg, 0.989 mmol), TBTA (0.262 mg, 0.495 mmol), and 1-octyne (1.46 mL, 9.89 mmol) and the mixture was refluxed under an inert atmosphere for 48 h. The cooled reaction mixture was diluted with DCM (100 mL) and EDTA/NH_4_OH_(aq)_ solution (0.1 M, 100 mL) and stirred vigorously for 4 h. The organic layer was separated and aqueous phase was extracted with DCM (2 × 50 mL). The combined organic layers were washed with brine (100 mL), dried over Na_2_SO_4_, filtered, and the solvents were removed under reduced pressure. The residue was purified by column chromatography (silica gel, 9:1 DCM/acetone) giving the product as an orange solid upon removal of solvents. Yield 268 mg, 91%. M. P. = 82°C; ^1^H NMR (400 MHz, CDCl_3_) δ 8.55 (d, *J* = 2.0 Hz, 1H, H_d_), 8.30 (s, 1H, H_g_), 8.14 (d, *J* = 8.4 Hz, 1H, H_f_), 7.94 (dd, *J* = 8.5, 2.1 Hz, 1H, H_e_), 4.55 (t, *J* = 1.7 Hz, 2H, H_c_), 4.31 (t, *J* = 1.8 Hz, 2H, H_b_), 4.27 (s, 5H, H_a_), 2.81 (t, *J* = 7.7 Hz, 2H, H_h_), 1.74 (p, *J* = 7.6 Hz, 2H, H_i_), 1.40 (m, 2H, H_j_), 1.33 (q, *J* = 3.4 Hz, 4H, H_k_ and H_l_), 0.89 (t, *J* = 7.0 Hz, 3H, H_m_); ^13^C NMR (101 MHz, CDCl_3_) δ ^13^C NMR (101 MHz, CDCl_3_) δ 150.7, 149.2, 147.5, 141.2, 120.9, 118.2, 113.2, 93.5, 81.8, 71.8, 70.2, 69.5, 64.1, 31.7, 29.4, 29.0, 25.8, 22.7, 14.2; IR (ATR): ν (cm^−1^) 3139, 2951, 2922, 2854, 2212, 1591, 1493, 1456, 1432, 1230, 1031, 815; HR–ESI MS: (MeOH) *m*/*z* = 439.1571 (calc. for C_25_H_27_N_4_Fe 439.1580), 461.1373 (calc. for C_25_H_26_N_4_Fe·Na 461.1399); UV-Vis (CH_2_Cl_2_) λ_max_ (ε/10^3^ L mol^−1^ cm^−1^) 307 (23), 444 (1); Anal. calc. for C_25_H_26_N_4_Fe C, 68.50; H, 5.98; N, 12.78%. Found C, 68.46; H, 6.18; N, 12.87%.

**Synthesis of L4:** 1,1′-Diiodoferrocene (469 mg, 1.07 mmol), 5-ethynyl-2-(4-hexyl-1H-1,2,3-triazol-1-yl)pyridine (600 mg, 2.36 mmol), CuI (40.8 mg, 0.214 mmol), [Pd(CH_3_CN)_2_(Cl)_2_] (16.7 mg, 0.064 mmol), and [PH(^t^Bu)_3_](BF_4_) (37.3 mg, 0.129 mmol) were combined in degassed diisopropylamine (15 mL) and stirred under microwave irradiation (200W) at 100 °C for 2 h. The crude reaction mixture was diluted with DCM (50 mL) and washed with EDTA/NH_4_OH_(aq)_ (0.1 M, 100 mL). The organic layer was separated and the aqueous phase extracted with DCM (4 × 50 mL). The combined organic layers were washed with brine (80 mL), dried over Na_2_SO_4_, filtered, and the solvent was removed under reduced pressure. The residue was purified by column chromatography (silica gel, gradient DCM, then 9:1 DCM/acetone) to give an orange solid upon removal of solvent. The product was further purified by precipitation from DCM with petrol, collection by filtration, and desiccating. Yield: 509 mg, 68%. M. P. = 144°C; ^1^H NMR (400 MHz, CDCl_3_) δ 8.35 (dd, *J* = 2.1, 0.8 Hz, 1H, H_c_), 8.10 (d, *J* = 0.8 Hz, 1H, H_f_), 8.04 (dd, *J* = 8.5, 0.8 Hz, 1H, H_e_), 7.84 (dd, *J* = 8.5, 2.2 Hz, 1H, H_d_), 4.61 (t, *J* = 1.9 Hz, 2H, H_b_), 4.40 (t, *J* = 1.9 Hz, 2H, H_a_), 2.76 (t, *J* = 7.7 Hz, 2H, H_g_), 1.72 (p, *J* = 7.3 Hz, 2H, H_h_), 1.45–1.37 (m, 2H, H_i_), 1.36–1.30 (m, 4H, H_j_ and H_k_), 0.89 (t, *J* = 7.0 Hz, 2H, H_l_); ^13^C NMR (101 MHz, CDCl_3_) δ 150.6, 149.2, 147.4, 141.0, 120.5, 118.0, 113.1, 91.7, 83.1, 73.2, 71.2, 66.7, 31.8, 29.3, 29.1, 25.8, 22.7, 14.2; IR (ATR): ν (cm^−1^) 3123, 3104, 2924, 2855, 2212, 1592, 1500, 1464, 1434, 1238, 1047, 823; HR-ESI MS: (MeOH) *m*/*z* = 713.2728 (calc. for C_40_H_42_N_8_Fe·Na 713.2775), 1403.5553 (calc. for C_80_H_84_N_16_Fe·Na 1403.5661); UV-Vis (CH_2_Cl_2_) λ_max_ (ε/10^3^ L mol^−1^ cm^−1^) 304 (45), 409 (1); Anal. calc. for C_40_H_42_N_8_Fe·0.3H_2_O C, 69.02; H, 6.17; N, 16.10%. Found C, 68.77; H, 6.21; N, 16.33%.

**General procedure for synthesis of [Ru(bipy)_2_]^2+^ complexes of L1**–**L4:** [Ru(bipy)_2_(Cl)_2_] (1.2 eq. for **L1** and **L2**, and 2.2 eq. for **L3** and **L4**) and AgBF_4_ (2.0 eq./Ru ion) were combined in degassed acetone (typically 5 mL) in the dark, and stirred at room temperature for two hours. The resulting AgCl precipitate was filtered off through celite, and the solution was added to a degassed acetone solution (typically 10 mL) of the desired ferrocene ligand under argon. The mixture was then refluxed in the dark overnight. The resulting solution was filtered through Celite and the solvent removed under reduced pressure. The residue was taken up in the minimum amount of DCM, precipitated by addition of diethyl ether, filtered over a vacuum and rinsed with diethyl ether (2 × 5 mL). The air-dried products were then further dried in a desiccator for at least 24 h.

**Synthesis of [L1Ru(bipy)_2_](BF_4_)_2_:** Following the general procedure with: 39.8 mg (0.082 mmol) of [Ru(bipy)_2_(Cl)_2_], 32.0 mg (0.164 mmol) of AgBF_4_, and 30.0 mg (0.068 mmol) of **L1**. The product was obtained as an orange powder. Yield: 65 mg (93%, based on **L1**). ^1^H NMR (400 MHz, *d*_6_-acetone) δ 9.26 (s, 1H, H_g_), 8.88–8.82 (m, 2H, H_bipy_), 8.81 (dt, *J* = 8.2, 1.1 Hz, 1H, H_bipy_), 8.77 (dt, *J* = 8.0, 1.0 Hz, 1H, H_bipy_), 8.37 (d, *J* = 8.3 Hz, 1H, H_f_), 8.31 (d, *J* = 5.6 Hz, 1H, H_bipy_), 8.27–8.11 (m, 7H, H_e_ and H_bipy_), 8.00 (ddd, *J* = 5.7, 1.6, 0.8 Hz, 1H, H_bipy_), 7.84 (s, 1H, H_d_), 7.66–7.58 (m, 3H, H_bipy_), 7.53 (ddd, *J* = 7.6, 5.7, 1.3 Hz, 1H, H_bipy_), 4.51 (t, *J* = 7.0 Hz, 2H, H_h_), 4.47 (s, 2H, H_c_), 4.36 (s, 2H, H_b_), 4.21 (s, 5H, H_a_), 1.87 (p, *J* = 7.1 Hz, 2H, H_i_), 1.24–1.11 (m, 6H, H_j_, H_k_, and H_l_), 0.81 (t, *J* = 6.9 Hz, 3H, H_m_); ^13^C NMR (101 MHz, CDCl_3_) δ 158.8, 158.6, 158.3, 158.1, 153.9, 153.3, 153.2, 153.1, 153.1, 150.5, 148.3, 141.0, 139.0 (2 coincident signals), 139.0, 138.7, 128.8, 128.7, 128.6, 127.9, 127.4, 125.4, 125.4, 124.9, 124.6, 123.7, 123.3, 96.6, 81.3, 72.5, 72.5, 70.9, 70.6, 65.1, 53.0, 31.6, 30.2, 26.3, 23.0, 14.2; IR (ATR): ν (cm^−1^) 3116, 2929, 2861, 2206, 1604, 1580, 1466, 1446, 1053, 1034, 765; HR–ESI MS: (MeOH) *m*/*z* = 426.0975 (calc. for C_45_H_42_FeN_8_Ru^2+^ 426.0964), 939.1941 (calc. for C_45_H_42_FeN_8_RuBF_4_^+^ 939.1969); UV-Vis (CH_2_Cl_2_) λ_max_ (ε/10^3^ L mol^−1^ cm^−1^) 288 (75), 422 (10), 449 (10); Anal. calc. for C_45_H_42_FeN_8_RuB_2_F_8_ C, 49.07; H, 3.84; N, 10.17%. Found C, 49.23; H, 3.76; N, 10.47%.

**Synthesis of [L2Ru(bipy)_2_](BF_4_)_2_:** Following the general procedure with: 39.8 mg (0.082 mmol) of [Ru(bipy)_2_(Cl)_2_], 32.0 mg (0.164 mmol) of AgBF_4_, and 30.0 mg (0.068 mmol) of **L2**. The product was obtained as a brown powder. Yield: 66 mg (94%, based on **L2**). ^1^H NMR (400 MHz, *d*_6_-acetone) δ 9.34 (s, 1H, H_g_), 8.89–8.84 (m, 2H, H_bipy_), 8.83 (d, *J* = 8.2 Hz, 1H, H_bipy_), 8.79 (d, *J* = 8.3 Hz, 1H, H_bipy_), 8.54 (d, *J* = 8.6 Hz, 1H, H_f_ and H_bipy_), 8.42–8.36 (m, 2H, H_e_ and H_bipy_), 8.31–8.17 (m, 5H, H_bipy_), 8.08–8.03 (m, 2H, H_d_ and H_bipy_), 7.99 (dd, *J* = 5.5, 1.2 Hz, 1H, H_bipy_), 7.64 (m, 3H, H_bipy_), 7.59–7.53 (m, 1H, H_bipy_), 4.46 (m, 2H, H_c_), 4.36 (t, *J* = 1.9 Hz, 2H, H_b_), 4.20 (s, 5H, H_a_), 2.75–2.70 (m, 1H, H_h_), 1.68–1.56 (m, 2H, H_i_), 1.31–1.19 (m, 6H, H_j_, H_k_, and H_l_), 0.84 (t, *J* = 6.5 Hz, 3H, H_m_); ^13^C NMR (101 MHz, CDCl_3_) δ 158.5, 158.5, 158.2, 157.9, 153.9, 153.4, 153.4, 153.4, 153.3, 153.0, 148.3, 143.2, 139.5, 139.5 (2 coincident signals), 139.2, 129.0, 128.8, 128.7, 128.2, 125.6, 125.5, 125.1, 124.8, 124.5, 124.0, 115.6, 97.2, 80.7, 72.5, 72.5, 70.9, 70.7, 63.6, 32.1, 29.2, 29.1, 26.2, 23.2, 14.3; IR (ATR): ν (cm^−1^) 3082, 2928, 2857, 2207, 1604, 1504, 1466, 1446, 1245, 1053, 1034, 764; HR–ESI MS: (MeOH) *m*/*z* = 426.0987 (calc. for C_45_H_42_FeN_8_Ru^2+^ 426.0964), 939.1963 (calc. for C_45_H_42_FeN_8_RuBF_4_^+^ 939.1969); UV-Vis (CH_2_Cl_2_) λ_max_ (ε/10^3^ L mol^−1^ cm^−1^) 285 (68), 428 (13); Anal. calc. for C_45_H_42_FeN_8_RuB_2_F_8_ C, 49.07; H, 3.84; N, 10.17%. Found C, 49.44; H, 4.03; N, 10.47%.

**Synthesis of [L3(Ru(bipy)_2_)_2_](BF_4_)_4_:** Following the general procedure with: 61.7 mg (0.127 mmol) of [Ru(bipy)_2_(Cl)_2_], 49.6 mg (0.255 mmol) of AgBF_4_, and 40.0 mg (0.058 mmol) of **L3**. The product was obtained as an orange powder. Yield: 100 mg (93%, based on **L3**). ^1^H NMR (400 MHz, *d*_6_-acetone) δ 9.22 (s, 2H, H_f_), 8.89 (dd, *J* = 8.5, 3.6 Hz, 2H, H_bipy_), 8.84–8.73 (m, 6H, H_bipy_), 8.31–8.07 (m, 15H, H_e’_ and H_bipy_), 8.04 (d, *J* = 8.3 Hz, 1H, H_e_), 8.01 (d, *J* = 5.6 Hz, 1H, H_bipy_), 7.98 (d, *J* = 5.7 Hz, 1H, H_bipy_), 7.88 (d, *J* = 1.7 Hz, 1H, H_c’_), 7.85 (dd, *J* = 8.2, 1.8 Hz, 1H, H_d’_), 7.76 (d, *J* = 1.8 Hz, 1H, H_c_), 7.70 (dd, *J* = 7.6, 5.6 Hz, 1H, H_bipy_), 7.66–7.50 (m, 7H, H_bipy_), 7.15 (dd, *J* = 8.4, 1.9 Hz, 1H, H_d_), 4.50 (t, *J* = 7.2 Hz, 4H, H_g_), 4.48–4.40 (m, 4H, H_b_), 4.38 (m, 4H, H_a_), 1.88 (m, 4H, H_h_), 1.26–1.08 (m, 12H, H_i_, H_j_, and H_k_), 0.84–0.76 (m, 6H, H_l_); Compound solubility insufficient for adequate ^13^C NMR collection; IR (ATR): ν (cm^−1^) 3115, 2930, 2862, 2212, 1603, 1465, 1445, 1240, 1052, 1032, 765; HR–ESI MS: (MeOH) *m*/*z* = 845.6856 (calc. for C_80_H_74_FeN_16_B_2_F_8_^2+^ 845.6910), 1778.3752 (calc. for C_80_H_74_FeN_16_B_3_F_12_^+^ 1778.3861); UV-Vis (CH_2_Cl_2_) λ_max_ (ε/10^3^ L mol^−1^ cm^−1^) 288 (137), 422 (24), 443 (1); Anal. calc. for C_80_H_74_FeN_16_B_4_F_16_·3.5H_2_O C, 49.84; H, 4.23; N, 11.62%. Found C, 49.61; H, 3.87; N, 11.43%.

**Synthesis of [L4(Ru(bipy)_2_)_2_](BF_4_)_4_:** Following the general procedure with: 30.9 mg (0.064 mmol) of [Ru(bipy)_2_Cl_2_], 24.8 mg (0.127 mmol) of [AgBF_4_], and 20.0 mg (0.029 mmol) of **L4**. The product was obtained as an orange powder. Yield: 40 mg (74%, based on **L4**). ^1^H NMR (400 MHz, *d*_6_-acetone) δ 9.26 (s, 2H, H_f_), 8.88–8.74 (m, 8H, H_bipy_), 8.51 (d, *J* = 8.7 Hz, 2H, H_e_), 8.38 (d, *J* = 5.6 Hz, 2H, H_bipy_), 8.23 (m, 10H, H_d_ and H_bipy_), 8.16 (d, *J* = 5.7 Hz, 2H, H_bipy_), 8.07 (d, *J* = 2.4 Hz, 2H, H_c_), 8.04 (d, *J* = 5.6 Hz, 2H, H_bipy_), 7.96 (d, *J* = 5.6 Hz, 2H, H_bipy_), 7.66–7.58 (m, 6H, H_bipy_), 7.55 (t, *J* = 6.8 Hz, 2H, H_bipy_), 4.52–4.44 (m, 4H, H_b_), 4.40 (s, 4H, H_a_), 2.75–2.69 (m, 4H, H_g_), 1.63 (d, *J* = 8.7 Hz, 4H, H_h_), 1.25 (m, 12H, H_i_, H_j_, and H_k_), 0.89–0.79 (m, 6H, H_l_); Compound solubility insufficient for adequate ^13^C NMR collection; IR (ATR): ν (cm^−1^) 3119, 2928, 2859, 2210, 1604, 1504, 1466, 1446, 1054, 1031, 765; HR–ESI MS: (MeOH) *m*/*z* = 845.6841 (calc. for C_80_H_74_FeN_16_B_2_F_8_^2+^ 745.6910), 1778.3734 (calc. for C_80_H_74_FeN_16_B_3_F_12_^+^ 1778.3861); UV-Vis (CH_2_Cl_2_) λ_max_ (ε/10^3^ L mol^−1^ cm^−1^) 285 (148), 427 (26); Anal. calc. for C_80_H_74_FeN_16_B_4_F_16_·5H_2_O C, 49.15; H, 4.33; N, 11.46%. Found C, 49.01; H, 4.07; N, 11.59%.

All cyclic voltammetry (CV) experiments were performed in solutions at 20 °C (DCM) with a concentration of 1 mM of electroactive analyte and 0.1 M NBu_4_PF_6_ as the supporting electrolyte. A three-electrode cell was used with Cypress Systems 1.4 mm diameter glassy carbon working, Ag/AgCl reference and platinum wire auxiliary electrodes. Voltammograms were recorded with the aid of a Powerlab/4sp computer-controlled potentiostat (AD instruments, Castle Hill, NSW, Australia). Potentials for all complexes were referenced to the reversible formal potential (taken as E° = 0.00 V) of the [Fc*]^+/0^ redox couple of decamethylferrocene [71]. In the cases of **L1**–**L4**, scans between −2.0 V and 1.7 V revealed no processes other than the ferrocenyl oxidation.

All photochemical experiments were carried out in a custom built UV photochemical reactor (at the University of Otago, Dunedin, NZ, USA), containing four UV-C lamps (Rayonet RPR-2537A, Southern New England Ultraviolet Co., Branford, CT, USA) emitting monochromatic 254 nm radiation, arranged symmetrically around the perimeter of the photoreactor. Samples were suspended in the centre of the UV-bulb arrangement, and an in-built fan was used to maintain a constant temperature of 35 °C during irradiation experiments.

## Figures and Tables

**Table 1:** Formal electrode potentials (E°) exhibited by **L1**–**L4** and **L1Ru**–**L4Ru** in dichloromethane (DCM) solution.

	E° (ΔE) (V (mV)) ^a^			
Compound	Bipy 1^(0/-)^	Bipy 2^(0/-)^	Fc^(+/0)^	Ru^(III/II)^
**L1**	-	-	0.63 (88)	-
**L2**	-	-	0.62 (86)	-
**L3**	-	-	0.69 (90)	-
**L4**	-	-	0.75 (84)	-
**L1Ru**	−1.51 (150)	−1.22 (80)	0.69 (79)	1.51 (110)
**L2Ru**	−1.38 (150)	−1.14 (86)	0.70 (76)	1.60 ^b^
**L3Ru**	−1.50 (200)	−1.21 (84)	0.83 (72)	1.48 (84)
**L4Ru**	−1.41 (200)	−1.15 (120)	0.83 (88)	1.57 (170)

^a^ E° values determined by cyclic voltammetry (CV) at 20 °C in DCM at 100 mVs^−1^, 0.1 M Bu_4_NPF_6_; E° = (E_pa_ + E_pc_)/2 and ΔE = |E_pa_ – E_pc_|, referenced to [Fc*]^(+/0)^. ^b^ E° determined from differential pulse voltammetry (DPV), as E_pa_ for the process in CV was at the limit of the solvent window.

## Supplementary Materials

The following are available online. Full experimental data of all the synthetic intermediates in the synthesis of **L2** and **L4**. Additionally, ^1^H and ^13^C NMR, ESI-MS, and UV-visible spectral data, CV and DPV plots, and X-ray crystallographic data for the compounds are provided. The computational methods are also detailed.
