# Supplementary material for: Synthesis and Light-Induced Actuation of Photo-Labile 2-Pyridyl-1,2,3-Triazole Ru(bis-bipyridyl) Appended Ferrocene Rotors
Source: Molecules. 2018 Aug 14;23(8):2037. doi: 10.3390/molecules23082037 (PMC6222349; doi:10.3390/molecules23082037)
Supplement: Supplementary file 1 [file molecules-23-02037-s001.pdf]

Supporting Information

Synthesis and Light Induced Actuation of Photo-labile 2-Pyridyl-1,2,3-Triazole Ru(bis-bipyridyl) Appended Ferrocene Rotors

James A. Findlay,[a] Jonathon E. Barnsley,[a] Keith C. Gordon,[a] and James D. Crowley*[a]*

[a]Department of Chemistry, University of Otago, PO Box 56, Dunedin,

New Zealand; Fax: +64 3 479 7906; Tel: +64 3 479 7731.

***jcrowley@chemistry.otago.ac.nz, finja479@student.otago.ac.nz**

Contents

1 Experimental Procedures 3
1.1 General Experimental Information 3
2 Synthetic Schemes for Ligands L1-4 4
3 Experimental Procedures 6
3.1 2-(6-bromo-3-pyridinyl)ethynylferrocene 6
3.2 2-(6-azido-3-pyridinyl)ethynylferrocene 7
3.3 1-(5-yl-Ethynyl-2-(4-hexyl-1H-1,2,3-triazol-1-yl)pyridine)ferrocene, L2 8
3.4 4-(6-azidopyridin-3-yl)-2-methylbut-3-yn-2-ol 9
3.5 4-(6-(4-hexyl-1H-1,2,3-triazol-1-yl)pyridin-3-yl)-2-methylbut-3-yn-2-ol 10
3.6 5-ethynyl-2-(4-hexyl-1H-1,2,3-triazol-1-yl)pyridine 11
3.7 1,1'-Di-(5-yl-ethynyl-2-(4-hexyl-1H-1,2,3-triazol-1-yl)pyridine)ferrocene, L4 12
3.8 $[L1Ru(bipy)_2](BF_4)_2$ 13
3.9 $[L2Ru(bipy)_2](BF_4)_2$ 15
3.10 $[L3(Ru(bipy)_2)_2](BF_4)_4$ 17
3.11 $[L4(Ru(bipy)_2)_2](BF_4)_4$ 19
3.12 $[Ru(bipy)_2(CH_3CN)_2](BF_4)_2$ 21
4 ^1H DOSY NMR Spectra 23
5 Electrochemistry 26
6 UV-Visible Spectra 31
7 UV-light Exposure Time Course Experiments ^1H NMR Stack Plots 33
8 Selected Thermal Re-coordination Experiment Attempt Results (^1H NMR and ESI-MS) 36
9 X-ray Crystallography Data 39
9.1 L2 ($P2_1/c$) 39
9.2 L4 ($P\bar{1}$) 41
10 Computational Information 43
11 References 48

1 Experimental Procedures

1.1 General Experimental Information

Unless otherwise stated, all reagents were purchased from commercial sources and used without further purification. Solvents were laboratory reagent grade. Petrol refers to the fraction of petroleum ether boiling in the range 40-60 °C. ^{1}H and ^{13}C NMR spectra were recorded on either a 400 MHz Varian 400 MR or Varian 500 MHz VNMRS spectrometer. Chemical shifts are reported in parts per million and referenced to residual solvent peaks ($CDCl_3$: ^{1}H δ 7.26 ppm, ^{13}C δ 77.16 ppm; CD_3CN: ^{1}H δ 1.94, ^{13}C δ 1.32, 118.26 ppm, d_6-acetone: ^{1}H δ 2.05 ppm; ^{13}C δ 29.84, 206.26 ppm). Coupling constants (*J*) are reported in Hertz (Hz). Standard abbreviations indicating multiplicity were used as follows: m = multiplet, q = quartet, t = triplet, dt = double triplet, d = doublet, dd = double doublet, s = singlet, atd = apparent triple doublet. Microanalyses were performed at the Campbell Microanalytical Laboratory at the University of Otago. Electrospray ionisation mass spectra (ESI - MS) were collected on a Bruker micro-TOF-Q spectrometer. UV-visible absorption spectra were acquired with a Shimadzu UV-2600-UV-Vis spectrophotometer.

Safety Note: Whilst no problems were encountered during the course of this work, azide compounds are potentially explosive and appropriate precautions should be taken when working with them.

2 Synthetic Schemes for Ligands L1-4

The syntheses of **L1** and **L3**, including all intermediates, are previously reported by us [1]. The synthetic schemes for the synthesis of **L2** and **L4** are shown below.

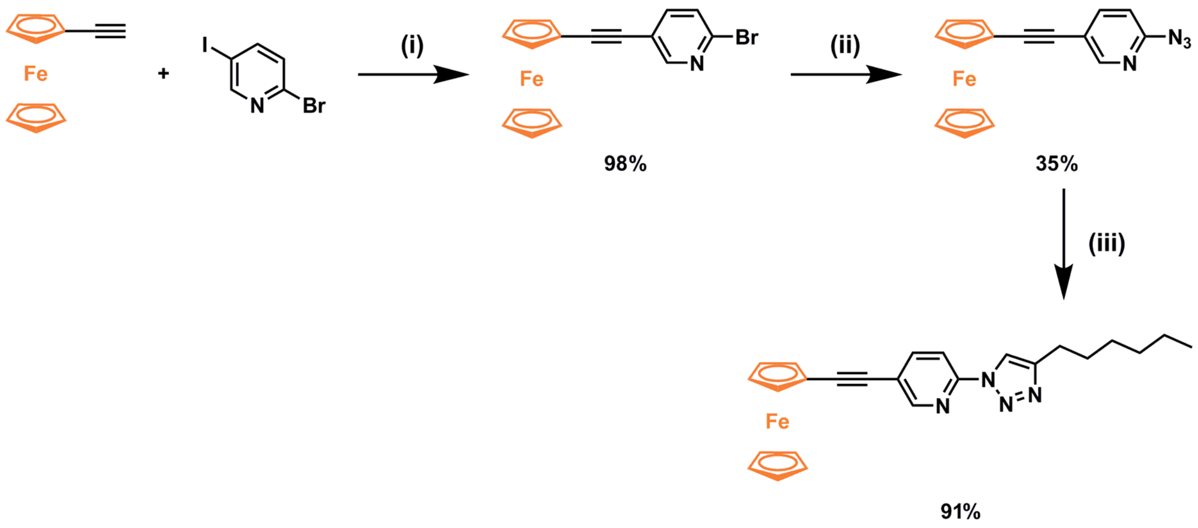

Scheme S1. Synthesis of **L2**. (i) $[Pd(PPh_3)_2(Cl)_2]$, CuI, triethylamine/THF (1:3, v/v), RT, overnight; (ii) sodium azide, CuI, sodium ascorbate, TMEDA, EtOH/H_2O (7:3 v/v), reflux, 7 hours; (iii) $[Cu(CH_3CN)_4](PF_6)$, TBTA, 1-octyne, toluene, reflux, 2 days.

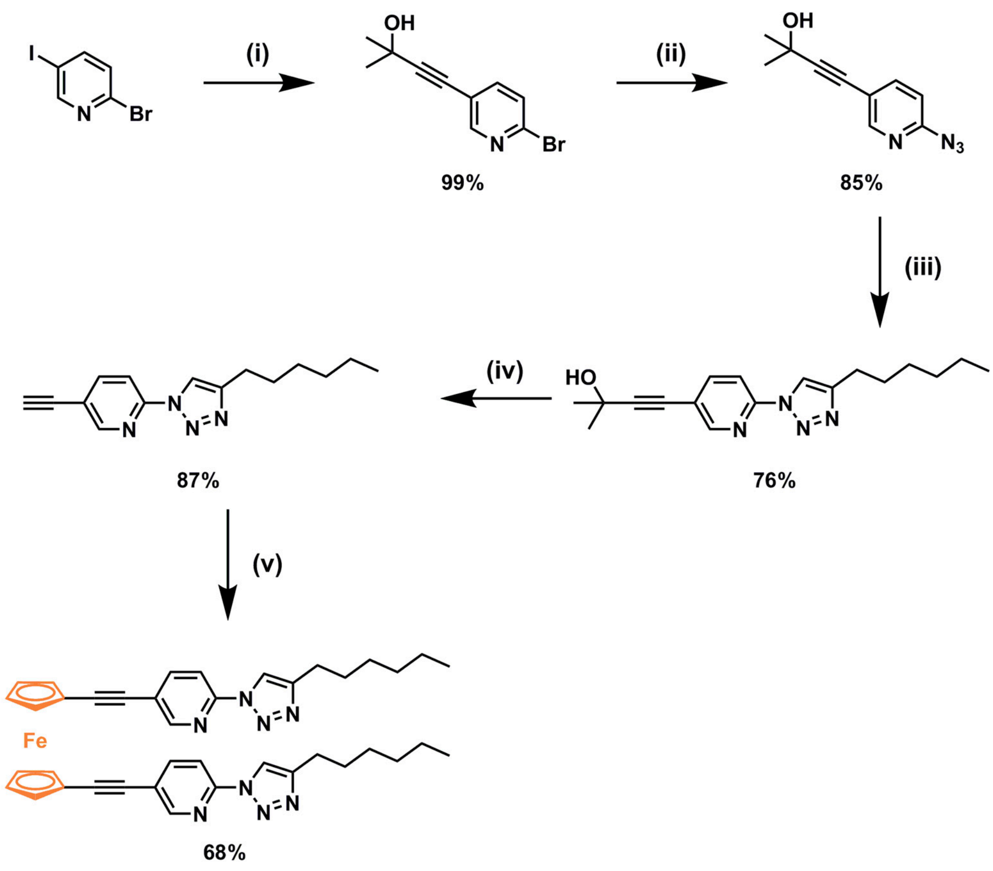

Scheme S2: Synthesis of **L4**. (i) 2-methylbut-3-yn-2-ol, [Pd(PPh$_3$)$_2$(Cl)$_2$], CuI, triethylamine/THF (1:3 v/v), RT, overnight; (ii) sodium azide, CuI, sodium ascorbate, TMEDA, ethanol/water (7:3), reflux, one hour; (iii) Cu(CH$_3$CN)$_4$PF$_6$, TBTA, toluene reflux, 2 days; (iv) NaOH, toluene, reflux 1 hour; (v) 1,1'-diiodoferrocene, [Pd(CH$_3$CN)$_2$(Cl)$_2$], CuI, [HP(*t*-Bu)$_3$](BF$_4$), diisopropylamine, microwave irradiation (200 W, 100 ˚C, 2 hours).

3 Experimental Procedures

3.1 2-(6-bromo-3-pyridinyl)ethynylferrocene

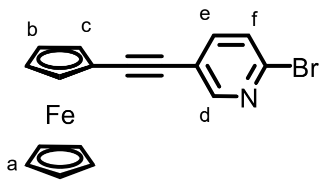

1-Ethynylferrocene (500 mg, 2.38 mmol) was dissolved in THF/TEA (20 mL, 1:1 v/v) and the solution was degassed (argon) for 10 minutes. 2-Bromo-5-iodopyridine (614 mg, 2.16 mmol), $[Pd(CH_3CN)_2Cl_2]_2$ (38 mg, 0.054 mmol) and CuI (41 mg, 0.216 mmol) were added and the mixture was stirred at RT under inert atmosphere overnight. The reaction mixture was diluted with CH_2Cl_2 (50 mL) and washed with EDTA/$NH_4OH_{(aq)}$ (0.1 M, 100 mL). The organic layer was separated and the aqueous layer was extracted with CH_2Cl_2 (2 x 50 mL). The combined organic layers were washed with brine (80 mL), dried over Na_2SO_4, filtered, and the solvent was removed under reduced pressure. Purification by column chromatography (silica gel, gradient 1:1 CH_2Cl_2/petrol to CH_2Cl_2 to 19:1 CH_2Cl_2/acetone) gave the product as an orange crystalline solid upon removal of solvent. Yield: 0.776 g, 98%. ^{1}H NMR (400 MHz, $CDCl_3$) 8.45 (d, J = 2.3 Hz, 1H, H_d), 7.59 (dd, J = 8.4, 2.4 Hz, 1H, H_e), 7.44 (d, J = 8.2 Hz, 1H, H_f), 4.53 (bs, 2H, H_c), 4.30 (bs, 2H, H_b), 4.26 (bs, 5H, H_a); ^{13}C NMR (100 MHz, $CDCl_3$) δ 152.2, 140.4, 140.3, 127.7, 120.6, 93.9, 81.4, 71.8, 70.3, 69.6, 64.1; HRESI-MS: (MeOH) m/z = 365.9575 (calc. for $C_{17}H_{13}NFeBr$ 365.9577); Anal. calc. for $C_{17}H_{12}NFeBr$•0.2 acetone C, 55.98; H, 3.52; N, 3.71%. Found C, 55.98; H, 3.27; N, 3.41%.

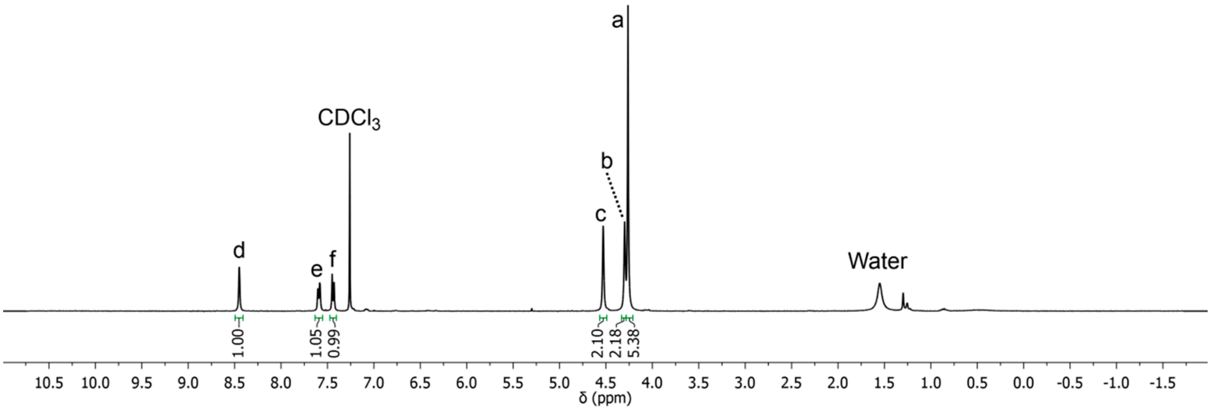

Figure S1: ^{1}H NMR (400 MHz, 298 K, $CDCl_3$) of 2-(6-bromo-3-pyridinyl)ethynylferrocene.

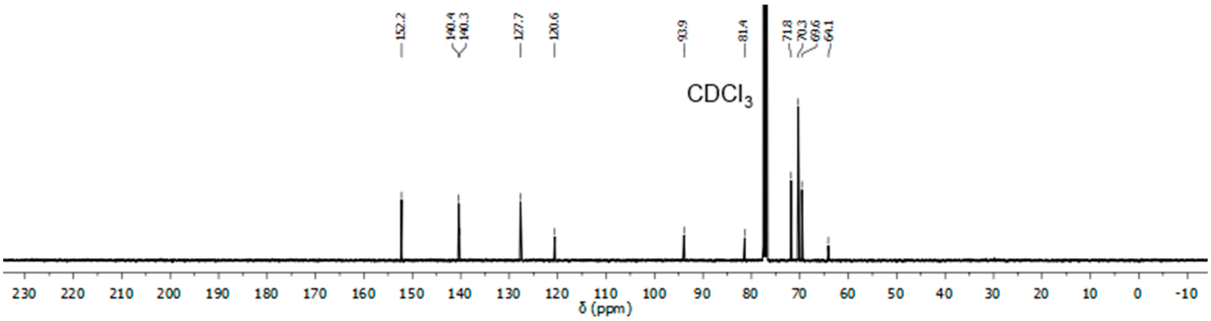

Figure S2: ^{13}C NMR (100 MHz, 298 K, $CDCl_3$) of 2-(6-bromo-3-pyridinyl)ethynylferrocene.

3.2 **2-(6-azido-3-pyridinyl)ethynylferrocene**

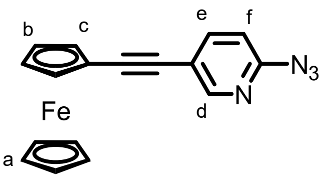

To a degassed ethanol/water mixture (17 mL, 7:3) was added 2-(6-bromo-3-pyridinyl) ethynylferrocene (700 mg, 1.91 mmol), sodium azide (249 mg, 3.82 mmol), CuI (36.4 mg, 0.191 mmol), sodium ascorbate (28.4 mg, 0.143 mmol) and tetramethylethylenediamine (0.043 mL, 0.287 mmol) against a flow of argon. The mixture was refluxed under an inert atmosphere for 7 hours. The cooled mixture was diluted with water (100 mL) and CH_2Cl_2 (50 mL) and the organic layer was separated. The aqueous phase was extracted with CH_2Cl_2 (3 x 50 mL). The combined organic layers were washed with brine (80 mL), dried over Na_2SO_4, filtered, and the solvents were removed under reduced pressure. The residue was purified by column chromatography (silica gel, gradient 1:1 CH_2Cl_2/petrol to CH_2Cl_2) to give an orange solid upon removal of solvent. *Fractions containing the desired product also contained the respective amino functionalised product: 2-(6-amino-3-pyridinyl)ethynylferrocene. Alteration of reaction conditions and eluents during chromatography could not prevent the formation or effect the separation of this side product. The mixture of desired azide and the side product amine was used in the next step with the molar fraction of azide approximated from the ^{1}H NMR.* ^{1}H NMR (400 MHz, $CDCl_3$) δ 8.91 (d, *J* = 1.3 Hz, 1H, H_d), 7.97 (d, *J* = 9.2 Hz, 1H, H_f), 7.69 (dd, *J* = 9.2, 1.4 Hz, 1H, H_e), 4.56 (t, *J* = 1.9 Hz, 2H, H_c), 4.33 (t, *J* = 1.9 Hz, 2H, H_b), 4.27 (s, 5H, H_a).

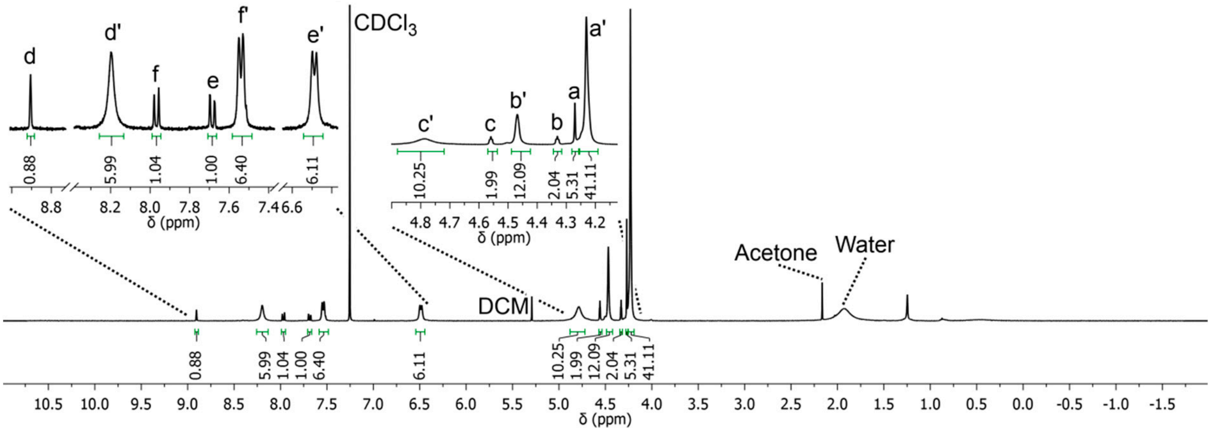

Figure S 3: ^{1}H NMR (400 MHz, 298 K, $CDCl_3$) of 2-(6-azido-3-pyridinyl)ethynylferrocene. Primed assignments correspond to equivalent environments in the amino side product.

3.3 1-(5-yl-Ethynyl-2-(4-hexyl-1H-1,2,3-triazol-1-yl)pyridine)ferrocene, L2

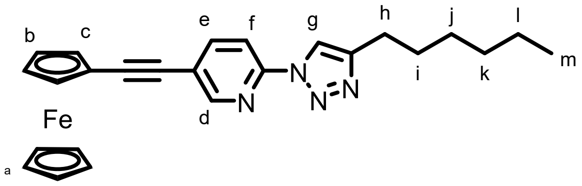

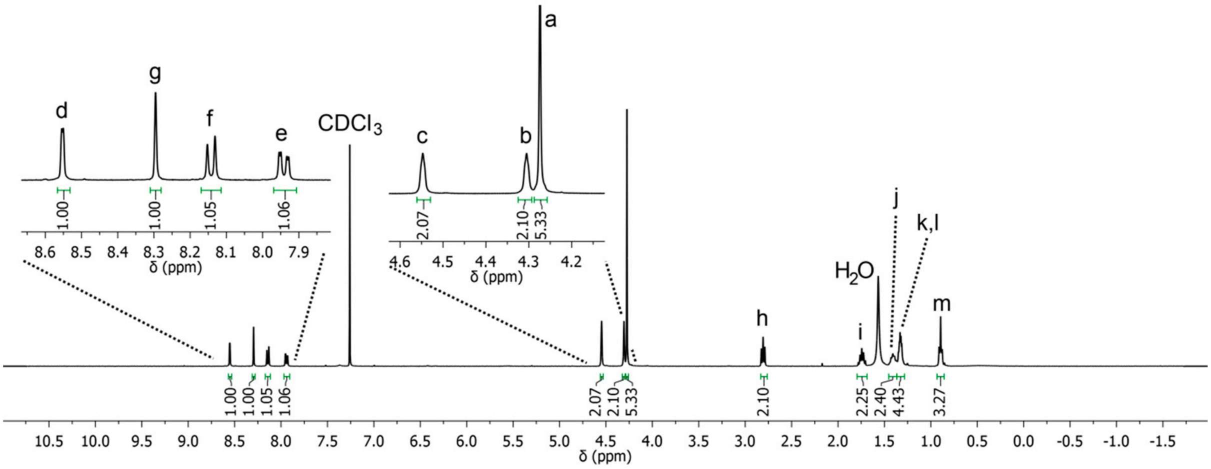

Figure S4: ^{1}H NMR (400 MHz, 298K, $CDCl_3$) of 1-(5-yl-Ethynyl-2-(4-hexyl-1H-1,2,3-triazol-1-yl)pyridine)ferrocene.

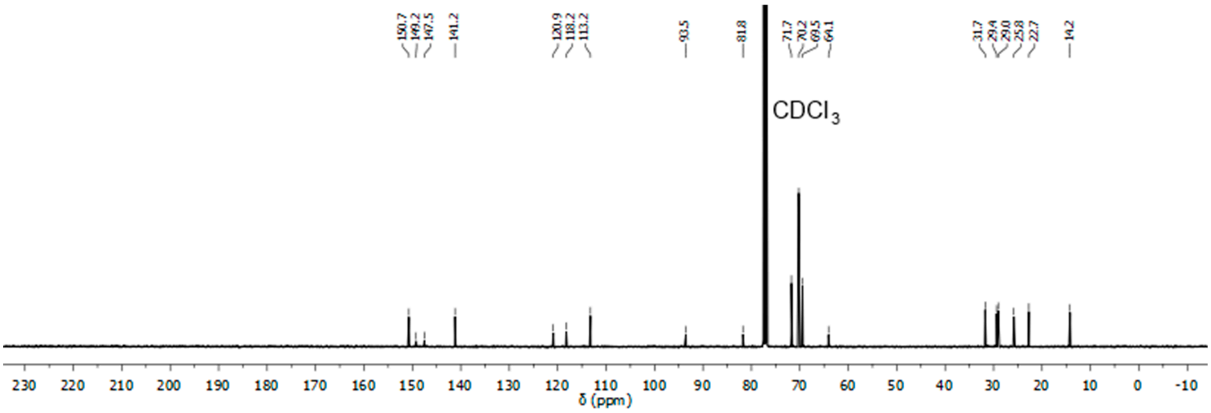

Figure S5: ^{13}C NMR (100 MHz, 298 K, $CDCl_3$) of 1-(5-yl-Ethynyl-2-(4-hexyl-1H-1,2,3-triazol-1-yl)pyridine)ferrocene.

3.4 4-(6-azidopyridin-3-yl)-2-methylbut-3-yn-2-ol

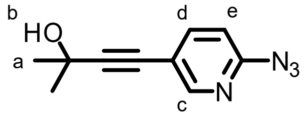

To a degassed ethanol/water mixture (60 mL, 7:3) was added 4-(6-bromopyridin-3-yl)-2-methylbut-3-yn-2-ol (1.85 g, 7.71 mmol), sodium azide (1.00 g, 15.4 mmol), CuI (0.147 g, 0.771 mmol), sodium ascorbate (0.076 g, 0.385 mmol) and TMEDA (0.134 g, 1.16 mmol) against a flow of argon. The mixture was refluxed under an inert atmosphere for one hour. The cooled mixture was diluted with water (100 mL) and CH_2Cl_2 (50 mL) and the organic layer was separated. The aqueous phase was extracted with CH_2Cl_2 (4 x 50 mL). The combined organic layers were washed with brine (80 mL), dried over Na_2SO_4, filtered, and the solvents were removed under reduced pressure. The residue was purified by column chromatography (deactivated (3% TEA in CH_2Cl_2) silica gel, 9:1 CH_2Cl_2/acetone) giving a white solid on removal of solvents. Yield: 1.56 g, 85%. ^{1}H NMR (400 MHz, $CDCl_3$) δ 8.88 (dd, *J* = 1.3, 1.1 Hz, 1H, H_c), 7.98 (dd, *J* = 9.3, 1.1 Hz, 1H, H_e), 7.63 (dd, *J* = 9.2, 1.5 Hz, 1H, H_d), 1.66 (s, 6H, H_a); ^{13}C NMR (100 MHz, $CDCl_3$) δ 147.6, 135.2, 127.7, 115.5, 113.9, 98.9, 76.2, 65.7, 31.3; HR-ESI MS: (MeOH) *m/z* = 203.0932 (calc. for $C_{10}H_{11}N_4O$ 203.0933), 225.0760 (calc. for $C_{10}H_{10}N_4O{\cdot}Na$ 225.0752); Anal. calc. for $C_{10}H_{10}N_4O{\cdot}$acetone C, 59.94; H, 5.28; N, 26.20%. Found C, 59.69; H, 5.04; N, 26.35%.

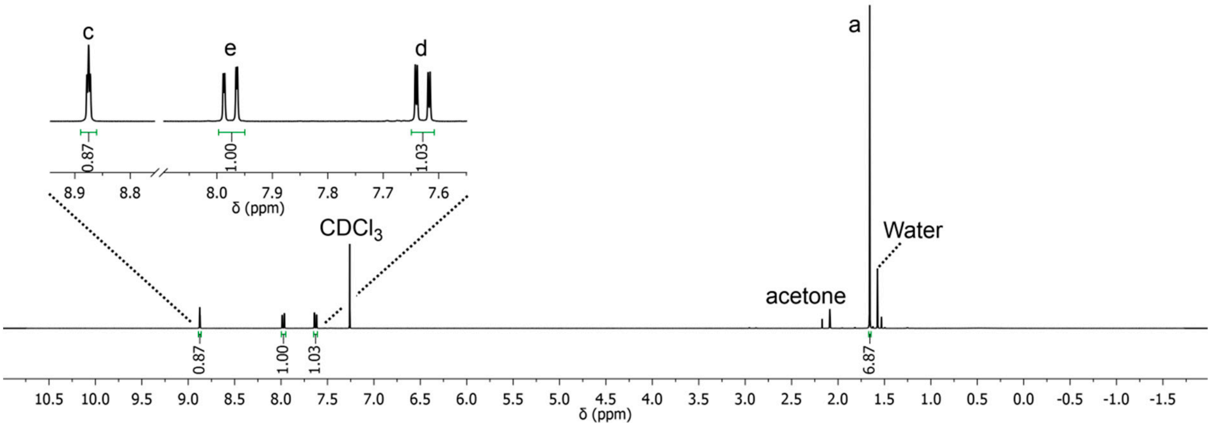

Figure S6: ^{1}H NMR (400 MHz, 298K, $CDCl_3$) of 4-(6-azidopyridin-3-yl)-2-methylbut-3-yn-2-ol.

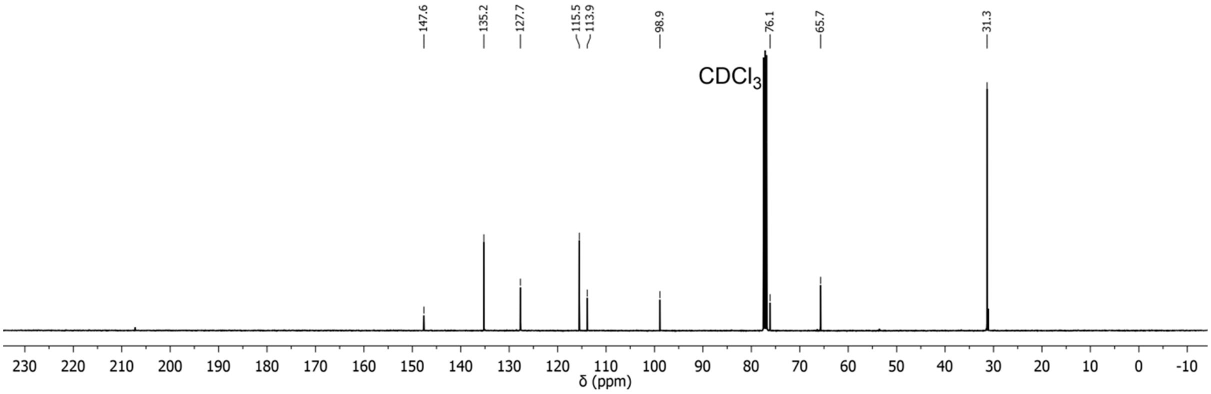

Figure S7: ^{13}C NMR (100 MHz, 298 K, $CDCl_3$) of 4-(6-azidopyridin-3-yl)-2-methylbut-3-yn-2-ol.

3.5 **4-(6-(4-hexyl-1H-1,2,3-triazol-1-yl)pyridin-3-yl)-2-methylbut-3-yn-2-ol**

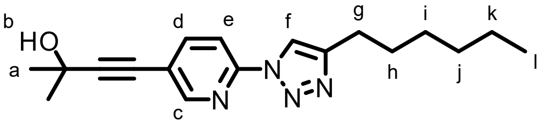

4-(6-azidopyridin-3-yl)-2-methylbut-3-yn-2-ol (1.00 g, 4.95 mmol), [Cu(CH_3CN)$_4$](PF_6) (0.369 g, 0.989 mmol), TBTA (0.495 g, 0.495 mmol) and 1-octyne (1.46 mL, 9.89 mmol) were added to degassed toluene (20 mL) against a flow of argon. The mixture was refluxed under an inert atmosphere for 48 hours. The cooled mixture was diluted with CH_2Cl_2 (100 mL) and EDTA/$NH_4OH_{(aq)}$ solution (0.1 M, 150 mL) and stirred vigorously for 4 hours. The organic layer was separated and aqueous phase was extracted with CH_2Cl_2 (3 x 50 mL). The combined organic layers were washed with brine (100 mL), dried over Na_2SO_4, filtered, and the solvents were removed under reduced pressure. The residue was purified by column chromatography (silica gel, 9:1 CH_2Cl_2/acetone) giving the product as an off white solid upon removal of solvents. Yield 1.17 g, 76%. ^{1}H NMR (400 MHz, $CDCl_3$) δ 8.50 (d, J = 2.2 Hz, 1H, H_c), 8.28 (s, 1H, H_f), 8.14 (d, J = 8.5 Hz, 1H, H_e), 7.89 (dd, J = 8.5, 2.2 Hz, 1H, H_d), 2.80 (t, J = 7.7 Hz, 2H, Hg), 1.73 (p, J = 7.5 Hz, 2H, H_h), 1.40 (t, J = 7.9 Hz, 2H, H_i), 1.32 (m, 4H, H_j and H_k), 0.89 (t, J = 7.0 Hz, 3H); ^{13}C NMR (100 MHz, $CDCl_3$) δ 151.2, 149.3, 148.2, 141.7, 119.6, 118.3, 113.2, 98.2, 78.2, 65.8, 31.7, 31.5, 29.4, 29.0, 25.8, 22.7, 14.2; HR-ESI MS: (MeOH) *m/z* = 335.1818 (calc. for $C_{18}H_{24}N_4O{\cdot}Na$ 335.1842), 647.3742 (calc. for $C_{36}H_{48}N_8O_2{\cdot}Na$ 647.3798); Anal. calc. for $C_{18}H_{24}N_4O$ C, 69.20; H, 7.74; N, 17.93%. Found C, 69.19; H, 8.05; N, 18.32%.

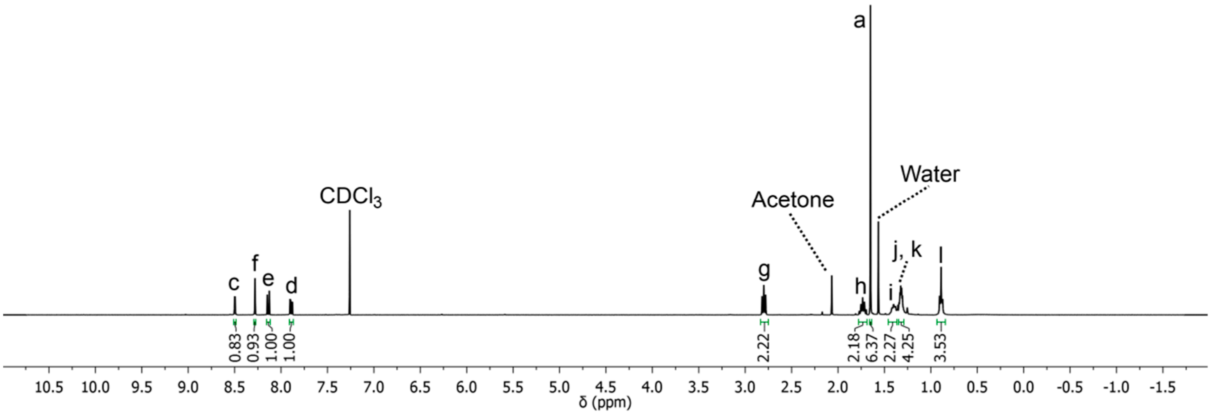

Figure S8: ^{1}H NMR (400 MHz, 298K, $CDCl_3$) of 4-(6-(4-hexyl-1H-1,2,3-triazol-1-yl)pyridin-3-yl)-2-methylbut-3-yn-2-ol.

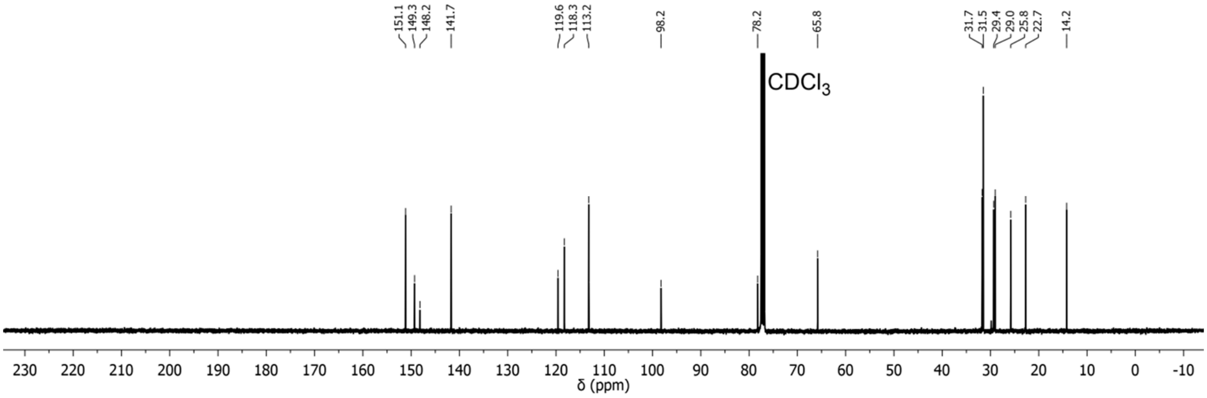

Figure S9: ^{13}C NMR (100 MHz, 298 K, $CDCl_3$) of 4-(6-azidopyridin-3-yl)-2-methylbut-3-yn-2-ol.

3.6 5-ethynyl-2-(4-hexyl-1H-1,2,3-triazol-1-yl)pyridine

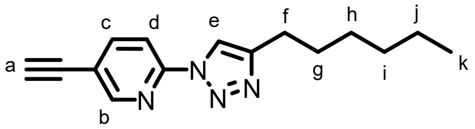

4-(6-(4-hexyl-1H-1,2,3-triazol-1-yl)pyridin-3-yl)-2-methylbut-3-yn-2-ol (1.17 g, 3.74 mmol), and ground sodium hydroxide pellets (0.374 g, 9.34 mmol) were added to a round bottom flask, suspended in toluene (30 mL) and heated to reflux for 2.5 hours. The crude reaction mixture was allowed to cool before being filtered through Celite and the solvent removed under reduced pressure. The residue was purified by column chromatography (silica gel, 9:1 CH_2Cl_2/acetone) giving a white solid upon removal of solvent. Yield: 0.830 g, 87%. ^{1}H NMR (400 MHz, $CDCl_3$) δ 8.58 (dd, *J* = 2.2, 0.8 Hz, 1H, H_b), 8.29 (d, *J* = 0.8 Hz, 1H, H_e), 8.17 (dd, *J* = 8.5, 0.9 Hz, 1H, H_d), 7.97 (dd, *J* = 8.5, 2.1 Hz, 1H, H_c), 3.29 (s, 1H, H_a), 2.80 (dt, *J* = 7.5, 0.8 Hz, 2H, H_f), 1.73 (p, *J* = 7.5 Hz, 2H, H_g), 1.45 – 1.37 (m, 2H, H_h), 1.35 – 1.29 (m, 4H, H_i and H_j), 0.89 (t, *J* = 7.0 Hz, 3H, H_k); ^{13}C NMR (100 MHz, $CDCl_3$) δ 151.7, 149.3, 148.5, 142.2, 118.9, 118.3, 113.2, 81.7, 79.6, 31.7, 29.3, 29.0, 25.8, 22.7, 14.2; IR (ATR): ν (cm^{-1}) 3189, 2956, 2921, 2855, 2104, 1587, 1485, 1475, 1434, 1223, 1039, 857; HR-ESI MS: (MeOH) *m/z* = 277.1415 (calc. for $C_{15}H_{18}N_4$·Na 277.1424), 531.2910 (calc. for $C_{30}H_{36}N_8$·Na 531.2961); Anal. calc. for $C_{15}H_{18}N_4$ C, 70.84; H, 7.13; N, 22.03%. Found C, 70.74; H, 7.12; N, 22.24%.

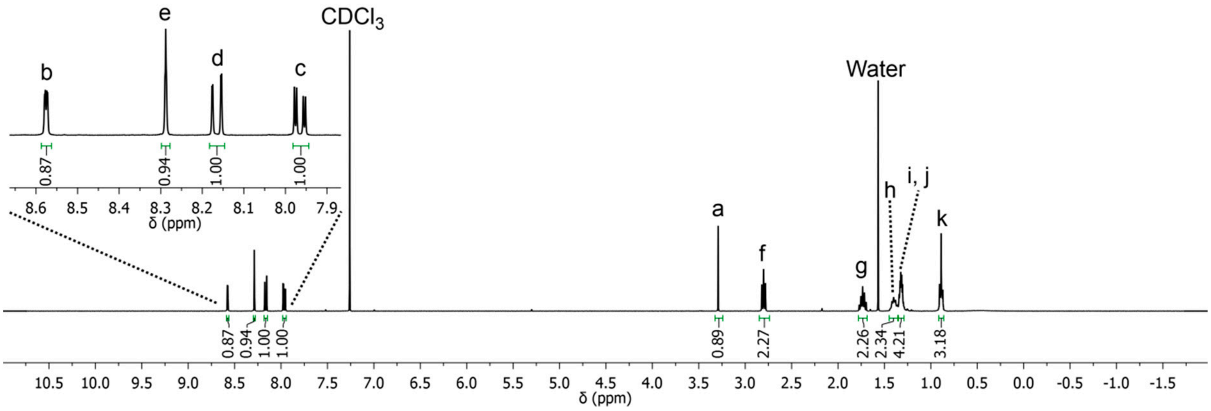

Figure S10: ^{1}H NMR (400 MHz, 298K, $CDCl_3$)of 5-ethynyl-2-(4-hexyl-1H-1,2,3-triazol-1-yl)pyridine.

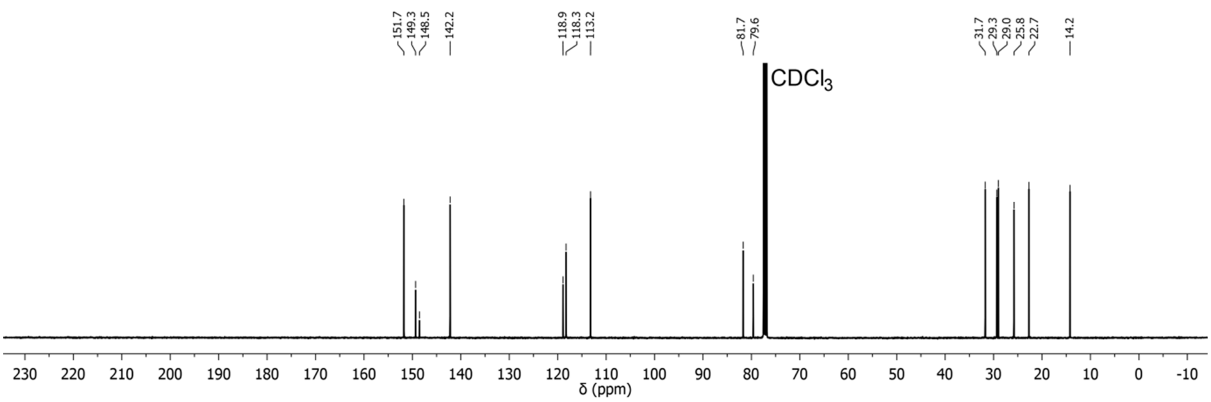

Figure S11: ^{13}C NMR (100 MHz, 298 K, $CDCl_3$) of 5-ethynyl-2-(4-hexyl-1H-1,2,3-triazol-1-yl)pyridine.

3.7 **1,1'-Di-(5-yl-ethynyl-2-(4-hexyl-1H-1,2,3-triazol-1-yl)pyridine)ferrocene, L4**

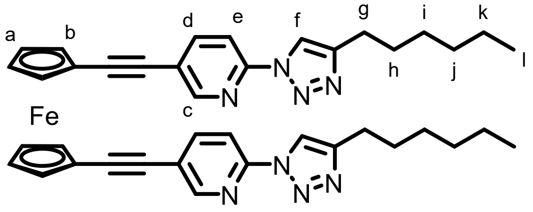

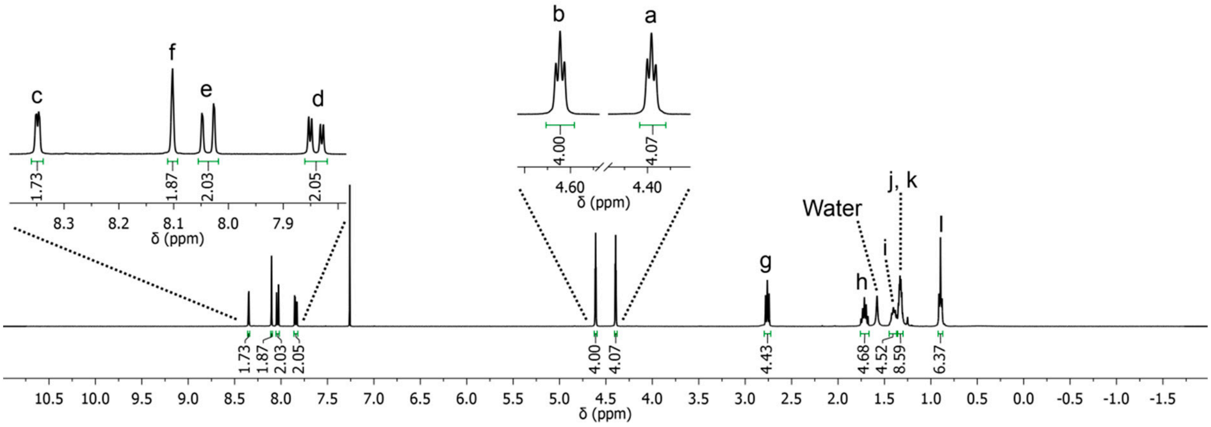

Figure S12: ^{1}H NMR (400 MHz, 298 K, $CDCl_3$) of 1,1'-Di-(5-yl-ethynyl-2-(1-hexyl-1H-1,2,3-triazol-4-yl)pyridine)ferrocene.

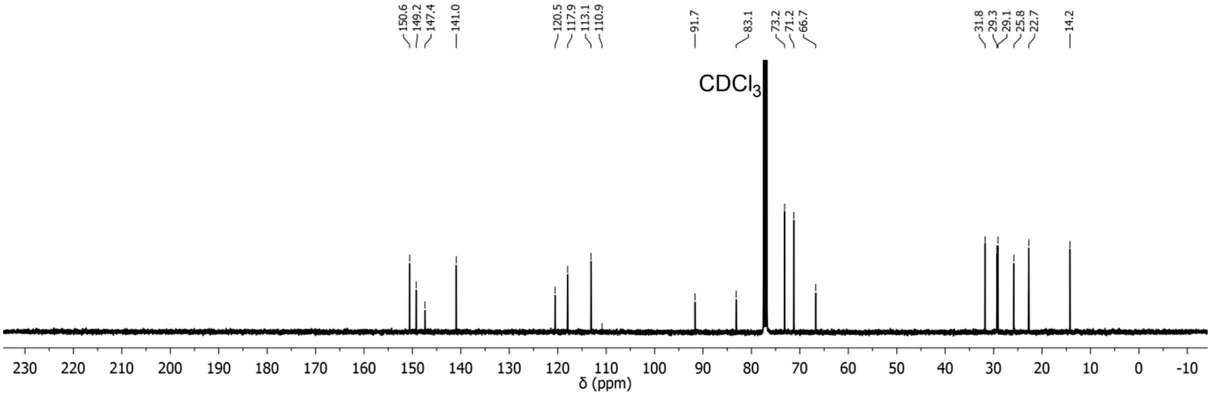

Figure S13: ^{13}C NMR (100 MHz, 298 K, $CDCl_3$) of 1,1'-Di-(5-yl-ethynyl-2-(1-hexyl-1H-1,2,3-triazol-4-yl)pyridine)ferrocene.

3.8 [L1Ru(bipy)$_2$](BF$_4$)$_2$

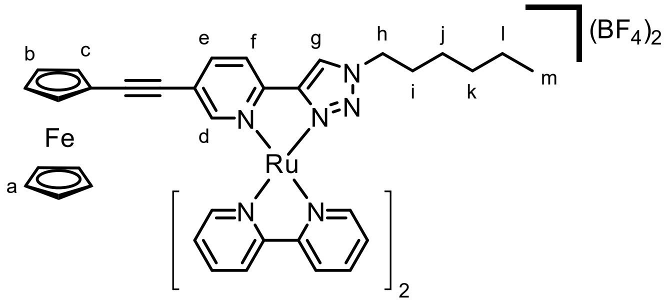

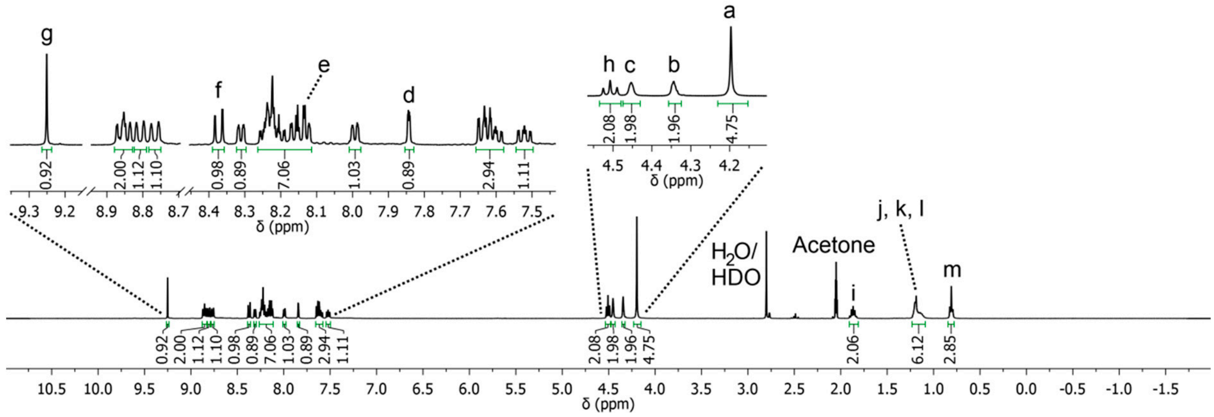

Figure S14: ^{1}H NMR (400 MHz, 298 K, d_6-acetone) of [**L1**Ru(bipy)$_2$](BF$_4$)$_2$.

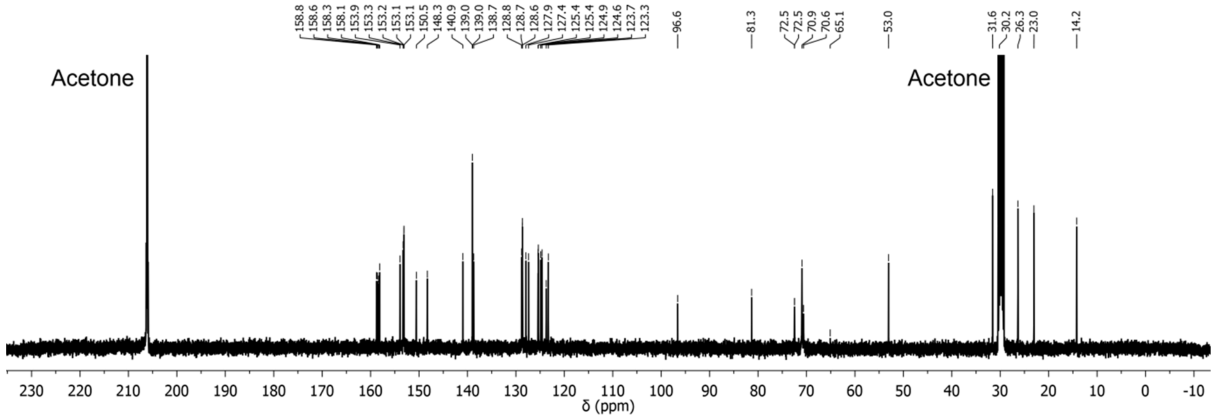

Figure S15: ^{13}C NMR (100 MHz, 298 K, d_6-acetone) of [**L1**Ru(bipy)$_2$](BF$_4$)$_2$.

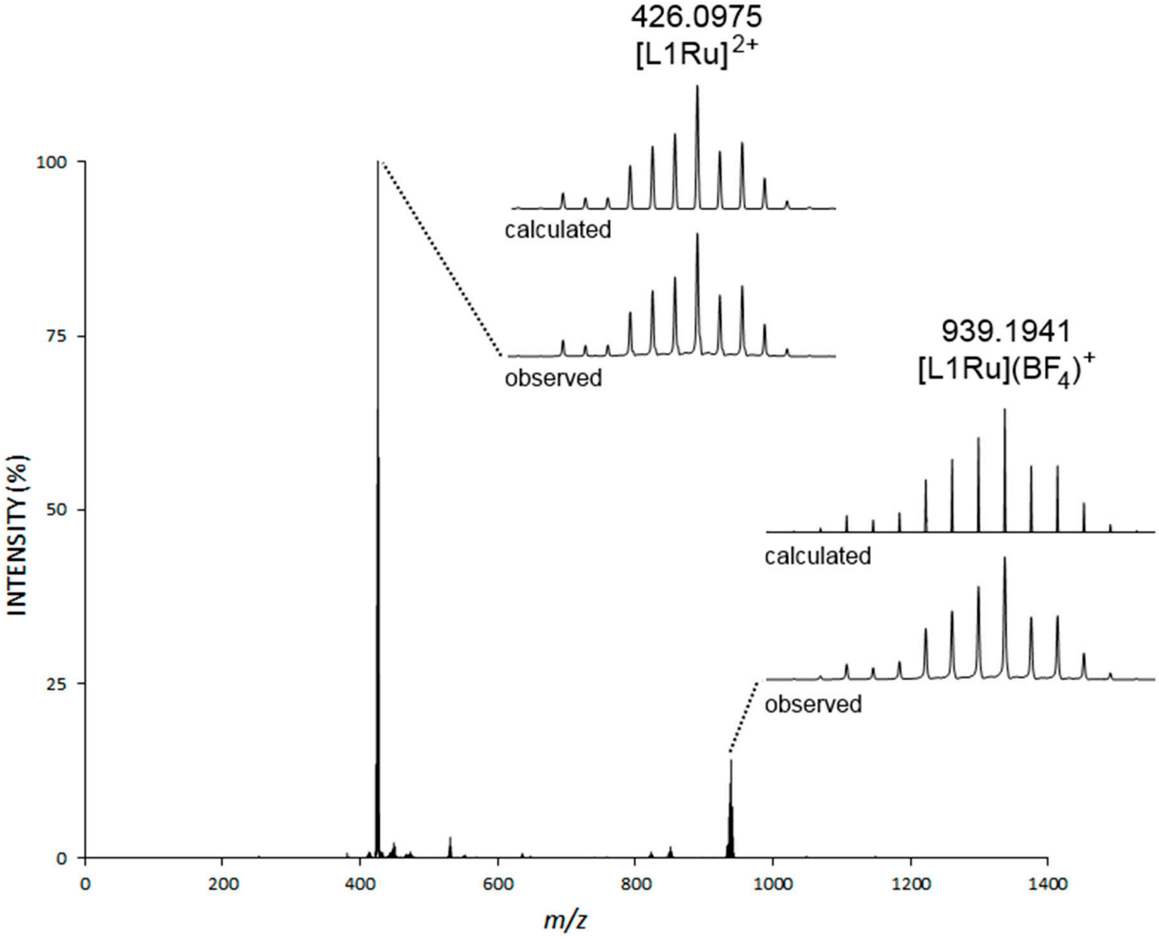

Figure S16: ESI-mass spectrum of [**L1**Ru(bipy)$_2$](BF$_4$)$_2$ (MeOH).

3.9 [L2Ru(bipy)$_2$](BF$_4$)$_2$

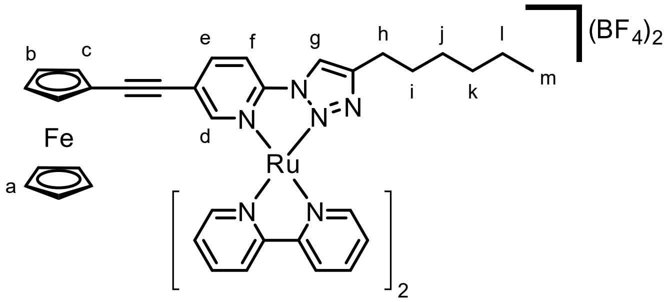

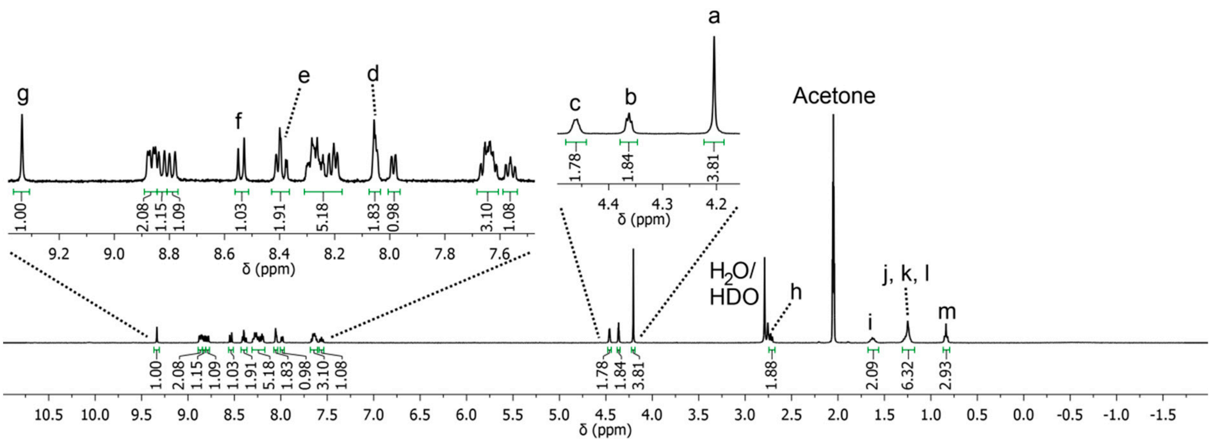

Figure S17: ^{1}H NMR (400 MHz, 298 K, d_6-acetone) of [**L2**Ru(bipy)$_2$](BF$_4$)$_2$.

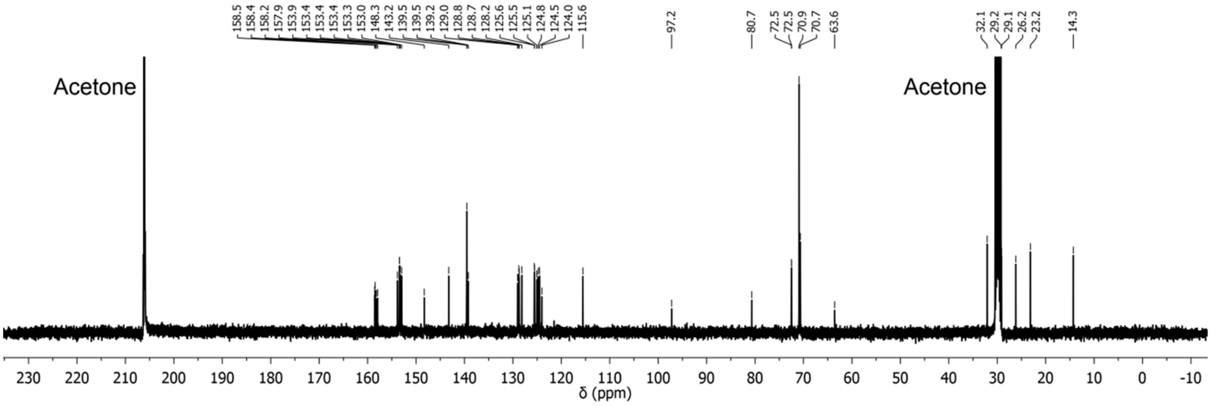

Figure S18: ^{13}C NMR (100 MHz, 298 K, CDCl$_3$) of [**L2**Ru(bipy)$_2$](BF$_4$)$_2$.

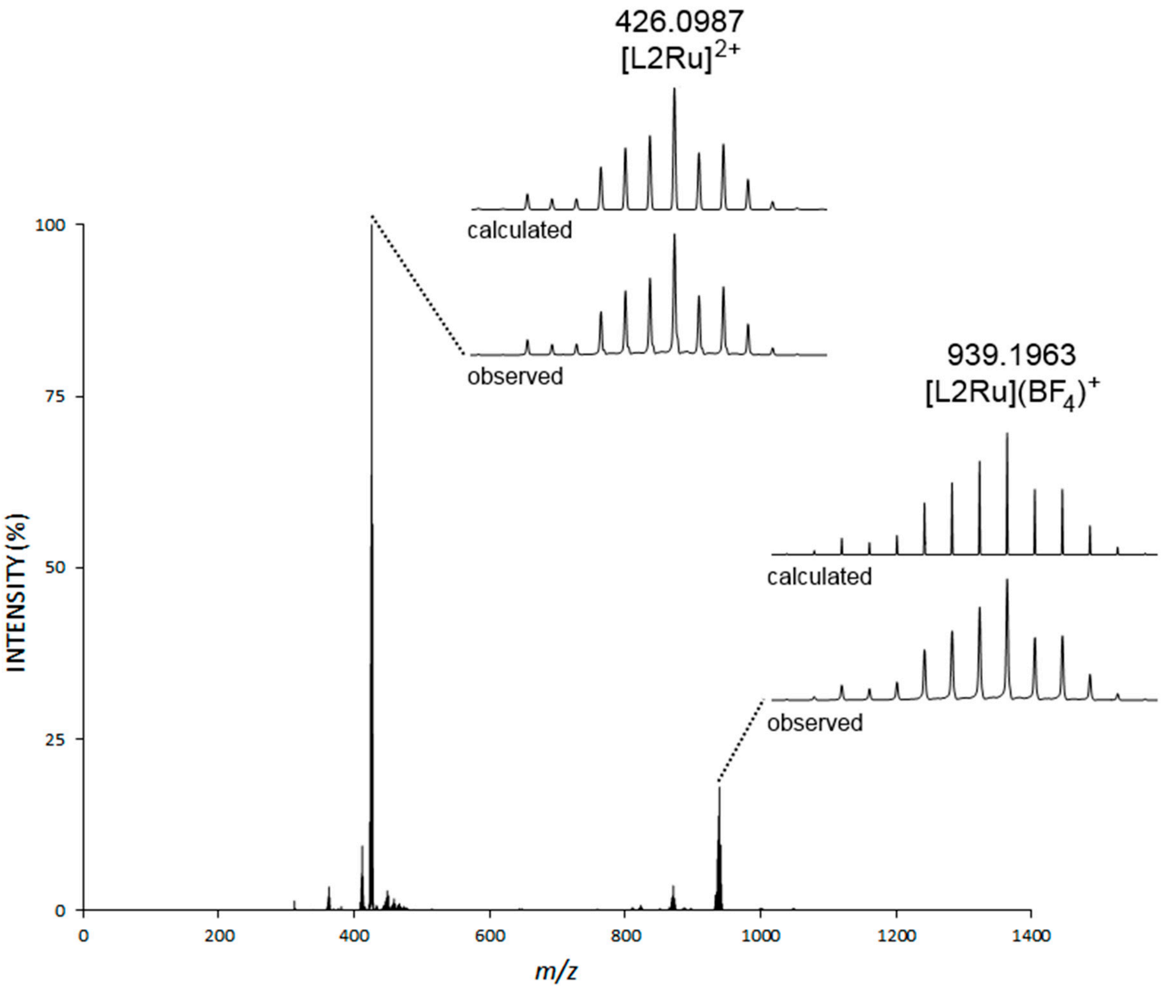

Figure S19: ESI-mass spectrum of [**L2**Ru(bipy)$_2$](BF$_4$)$_2$ (MeOH).

3.10 **[L3(Ru(bipy)$_2$)$_2$](BF$_4$)$_4$**

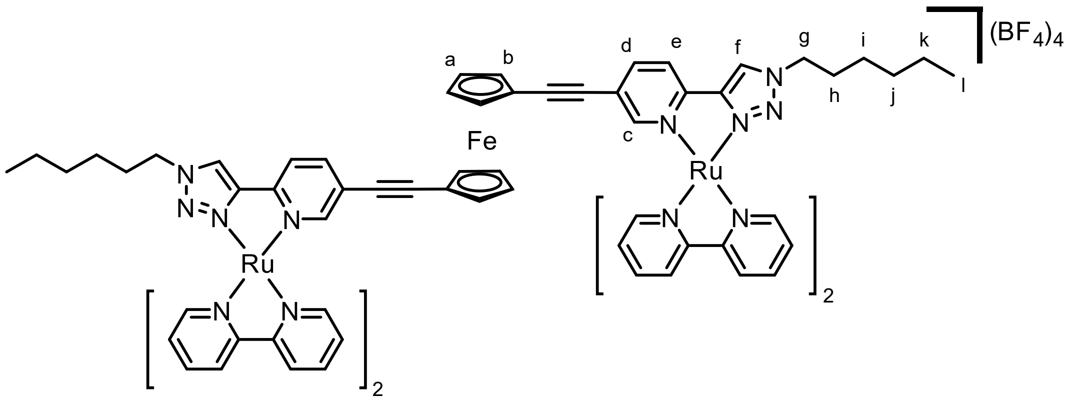

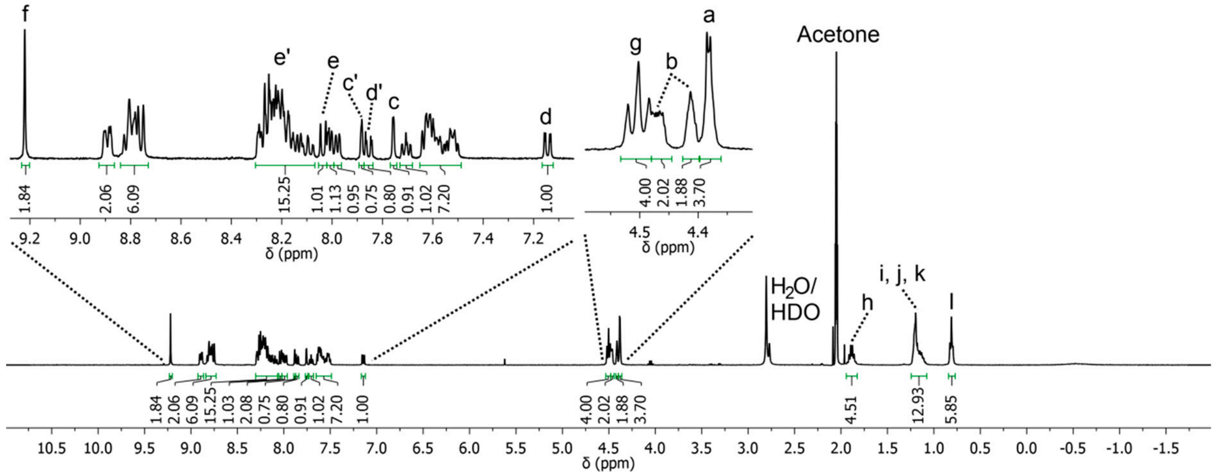

Figure S20: ^{1}H NMR (400 MHz, 298 K, d_6-acetone) of [**L3**(Ru(bipy)$_2$)$_2$](BF$_4$)$_4$.

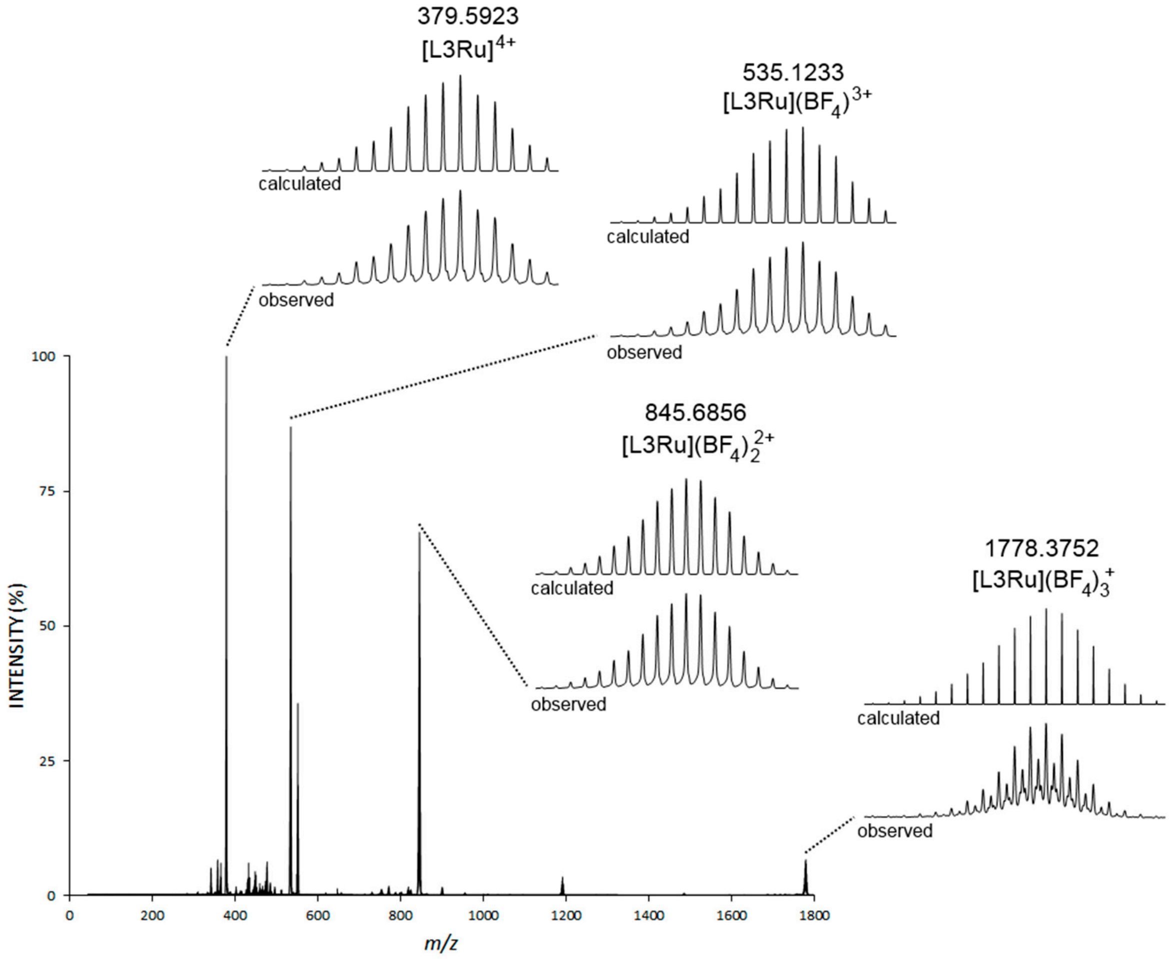

Figure S21: ESI-mass spectrum of [**L3**(Ru(bipy)$_2$)$_2$](BF$_4$)$_4$ (MeOH).

3.11 **[L4(Ru(bipy)$_2$)$_2$](BF$_4$)$_4$**

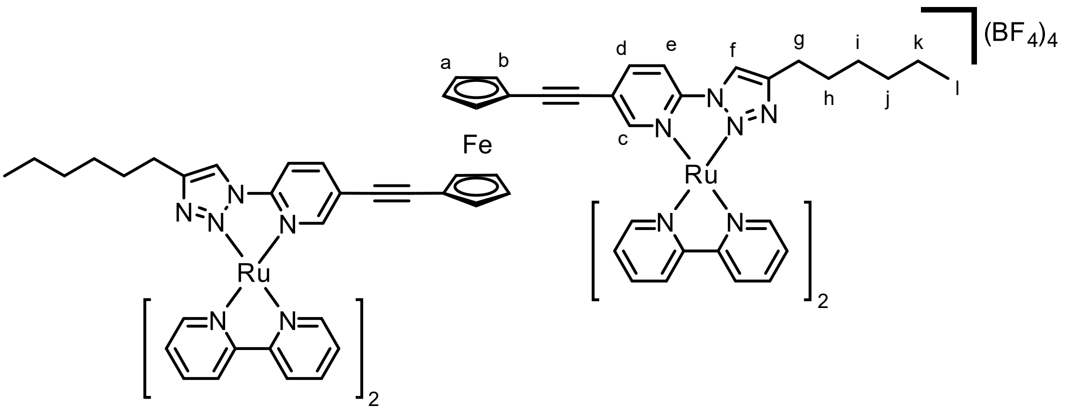

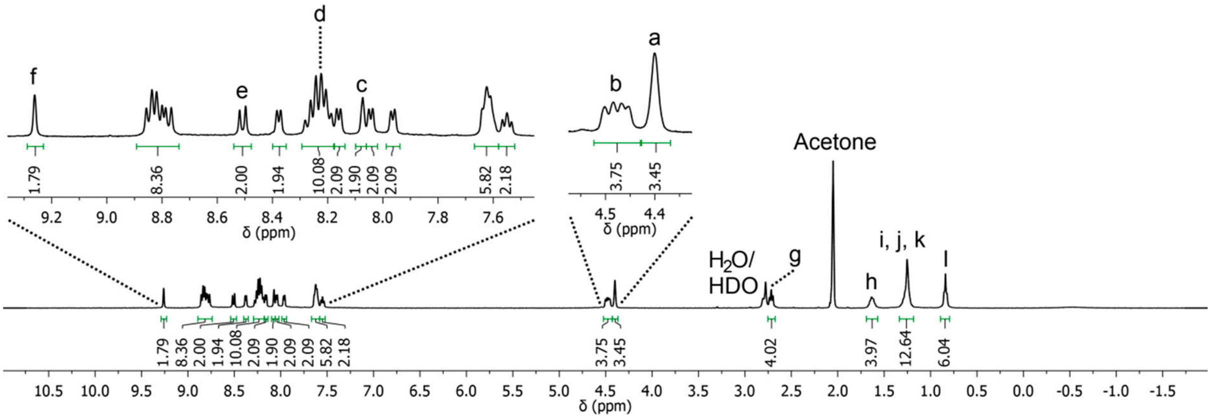

Figure S22: ^{1}H NMR (400 MHz, 298 K, d_6-acetone) of [**L4**(Ru(bipy)$_2$)$_2$](BF$_4$)$_4$.

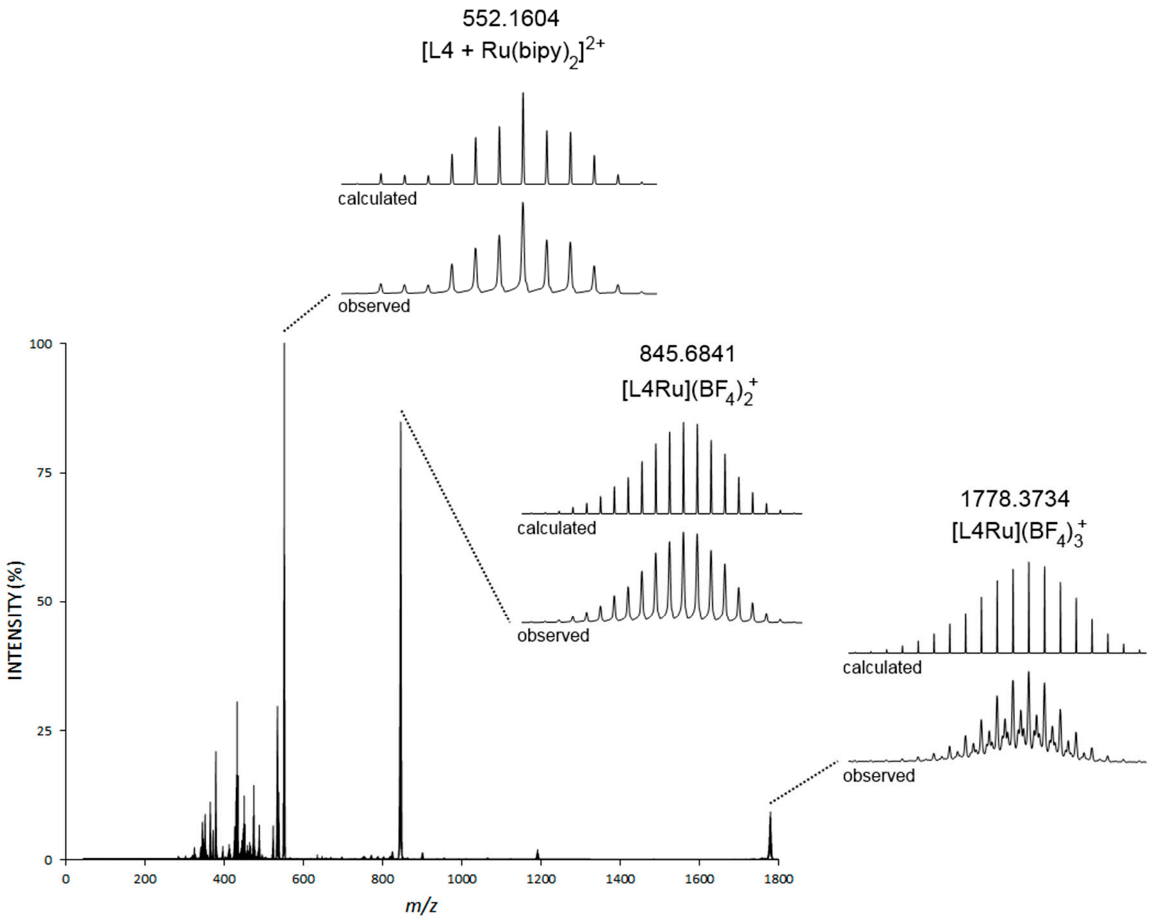

Figure S23: ESI-mass spectrum of [**L4**(Ru(bipy)$_2$)$_2$](BF$_4$)$_4$ (MeOH).

3.12 $[Ru(bipy)_2(CH_3CN)_2](BF_4)_2$

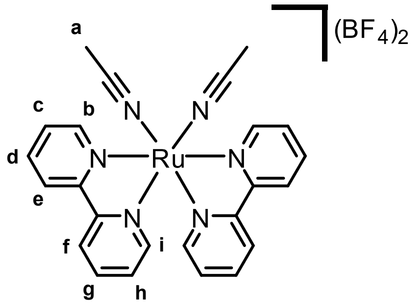

$[Ru(bipy)_2(Cl)_2]$ (30.0 mg, 0.062 mmol) and $[AgBF_4]$ (26.5 mg, 0.136 mmol, 2.2 eq.) were combined in degassed CH_3CN and stirred in the dark at RT for two hours. The resulting suspension was filtered through Celite and the solvent was removed under reduced pressure. The residue was taken up in the minimum volume of CH_2Cl_2, filtered through Celite again, and then precipitated via addition of diethyl ether. The orange powder was collected by vacuum filtration, rinsed with diethyl ether (2 x 5 mL), dried in air and then finally in a desiccator overnight. Yield: 25 mg (61%). ^1H NMR (400 MHz, CD_3CN) δ 9.26 (ddd, J = 5.5, 1.6, 0.7 Hz, 2H, H_b), 8.47 (dt, J = 8.0, 1.0 Hz, 2H, H_e), 8.32 (dt, J = 8.2, 0.9 Hz, 2H, H_f), 8.23 (atd, J = 7.9, 1.5 Hz, 2H, H_d), 7.90 (atd, J = 7.9, 1.5 Hz, 2H, H_g), 7.80 (ddd, J = 7.6, 5.6, 1.3 Hz, 2H, H_c), 7.54 (ddd, J = 5.6, 1.5, 0.7 Hz, 2H, H_i), 7.21 (ddd, J = 7.6, 5.6, 1.3 Hz, 2H, H_h), 2.22 (s, 6H, H_a); HR-ESI MS: (CH_3CN) m/z = 225.0325 (calc. for $C_{20}H_{16}N_4Ru(H_2O)_2^{2+}$ 225.0312), 474.0664 (calc. for $C_{20}H_{16}N_4Ru(CH_3CN)F^+$ 474.0668), 583.0952 (calc. for $C_{20}H_{16}N_4Ru(CH_3CN)_2BF_4^+$ 583.0983).

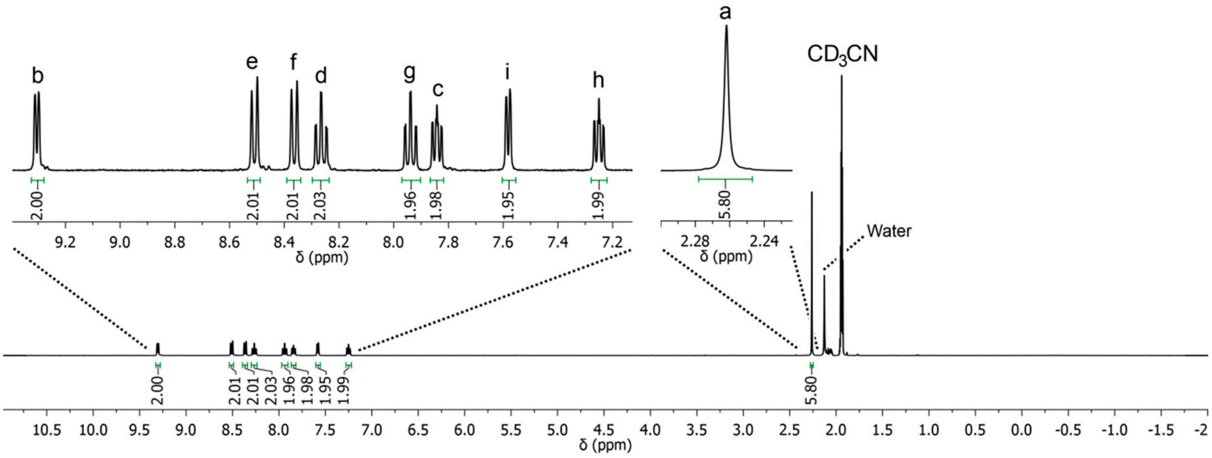

Figure S24: ^1H NMR (400 MHz, 298 K, CD_3CN) of $[Ru(bipy)_2(CH_3CN)_2](BF_4)_2$.

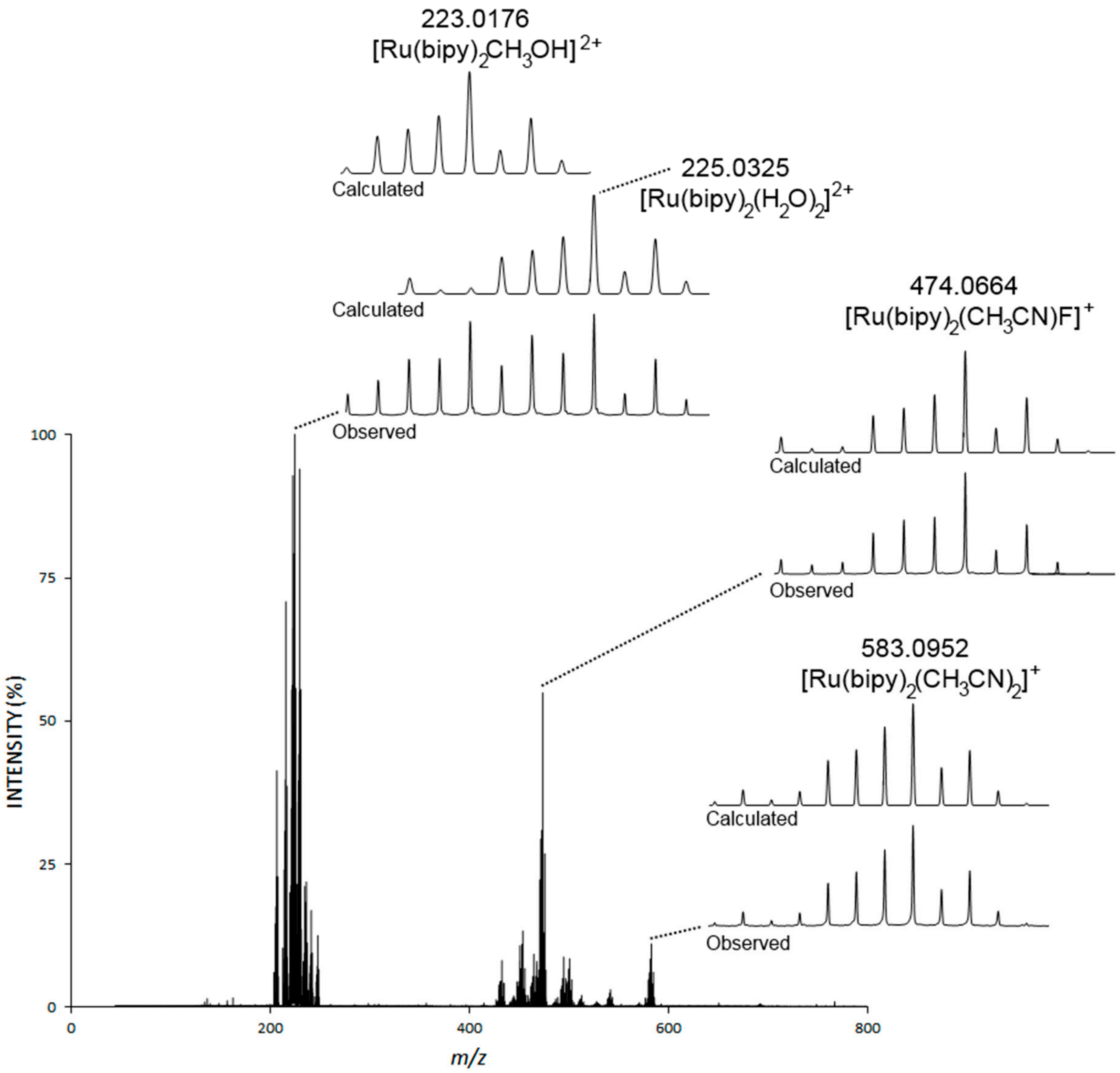

Figure S25: ESI-mass spectrum of $[Ru(bipy)_2(CH_3CN)_2](BF_4)_2$ (MeOH/CH_3CN).

4 ^{1}H DOSY NMR Spectra

Table S1: Molecular weights and diffusion coefficients as determined by ^{1}H DOSY NMR (d$_6$-acetone, 500 MHz, 298 K) for **L1Ru-L4Ru**.

Species	Molecular Weight (gmol^{-1})	Diffusion Coefficient (x 10^{-10} m^2 s^{-1})
**L1Ru**	1025	8.7
**L2Ru**	1025	8.7
**L3Ru**	1865	7.0
**L4Ru**	1865	6.4

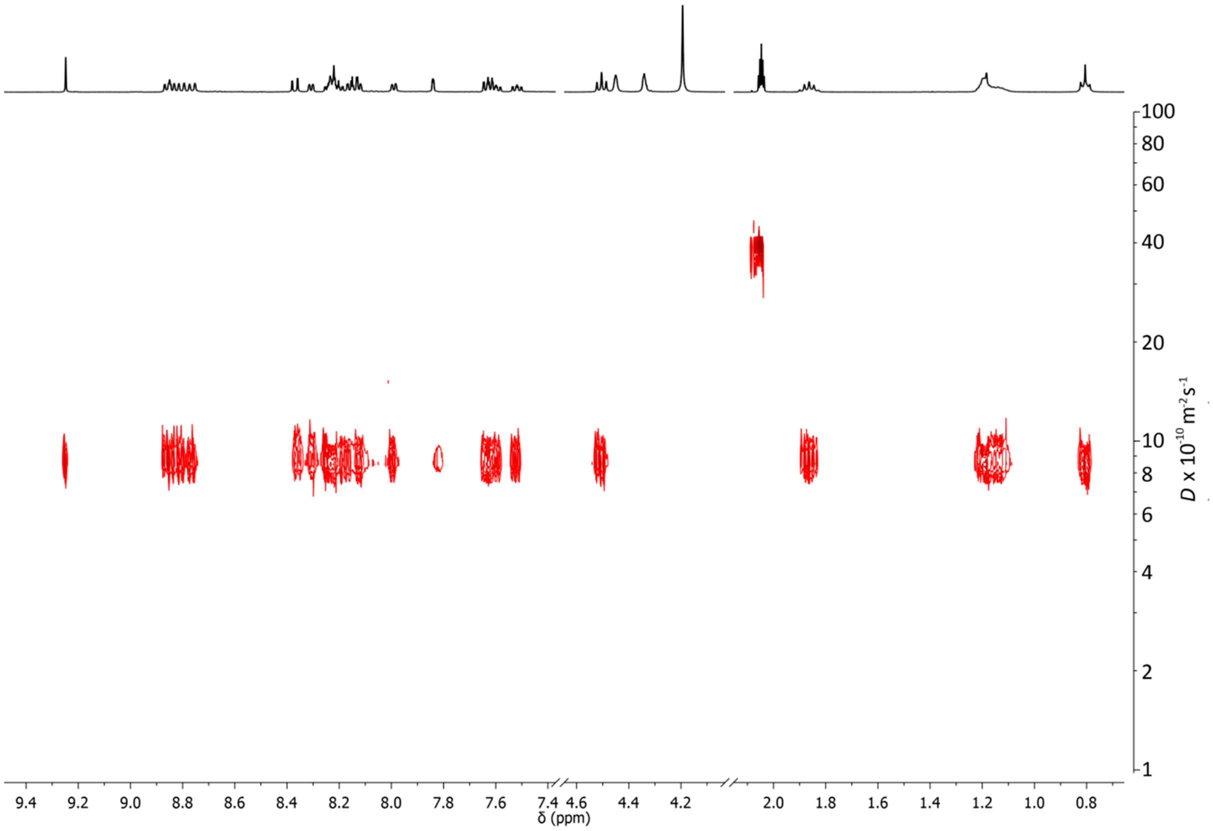

Figure S26: ^{1}H DOSY NMR (d$_6$-acetone, 500 MHz, 298 K) of **L1Ru**.

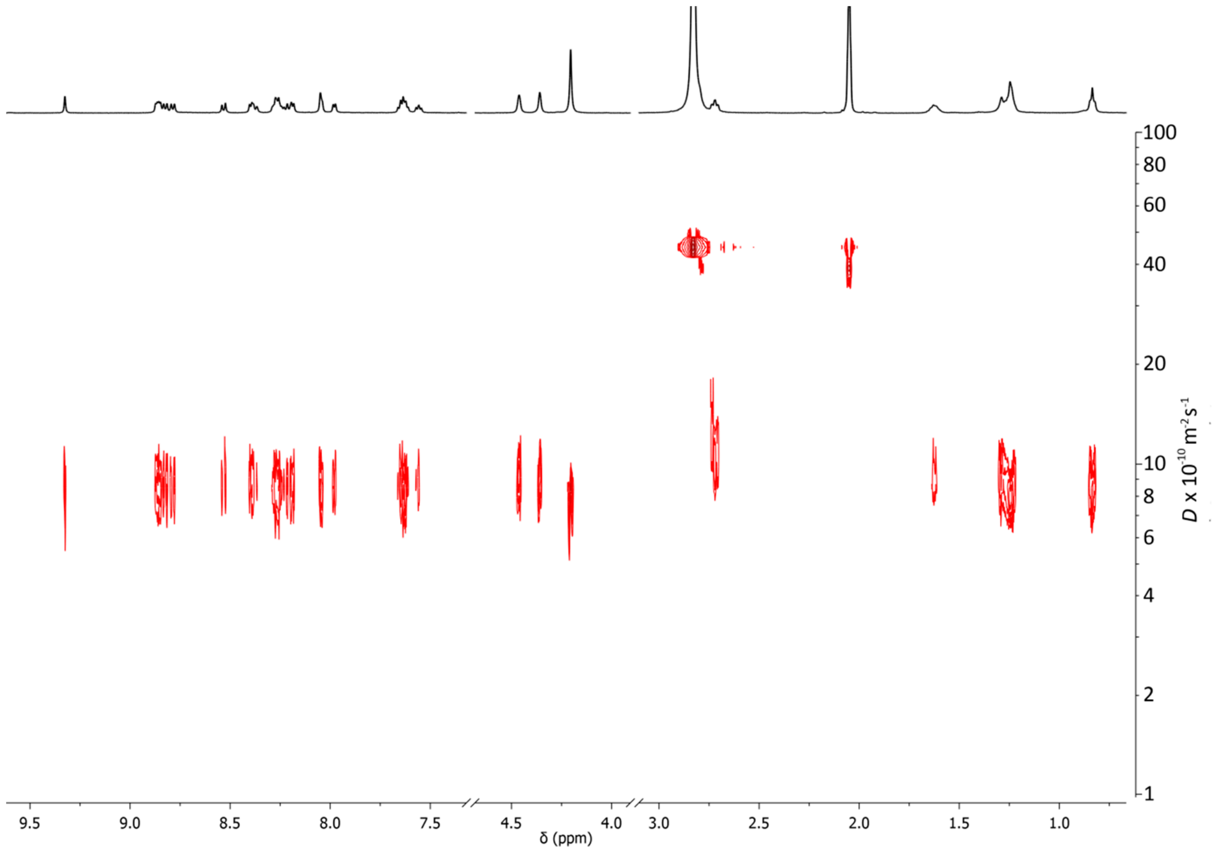

Figure S27: ^{1}H DOSY NMR (d_6-acetone, 500 MHz, 298 K) of **L2Ru**.

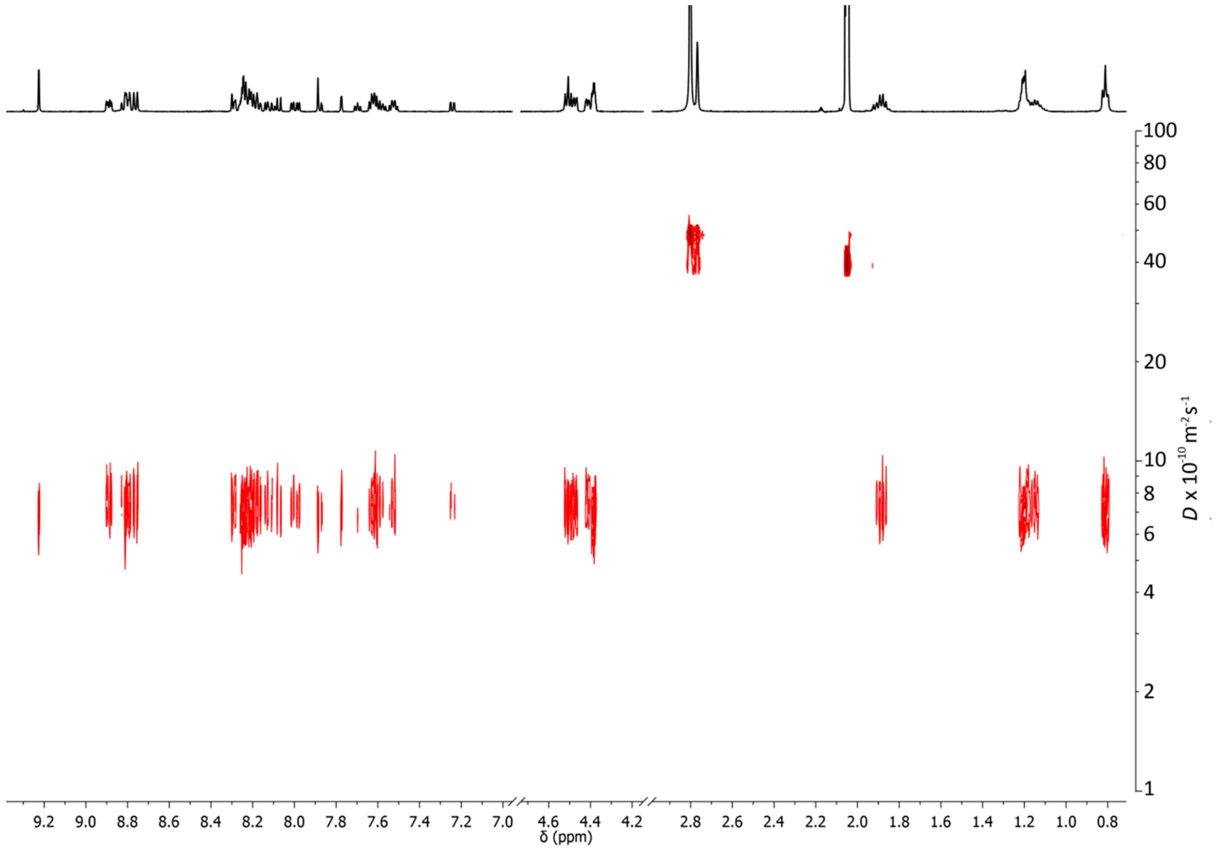

Figure S28: ^{1}H DOSY NMR (d_6-acetone, 500 MHz, 298 K) of **L3Ru**.

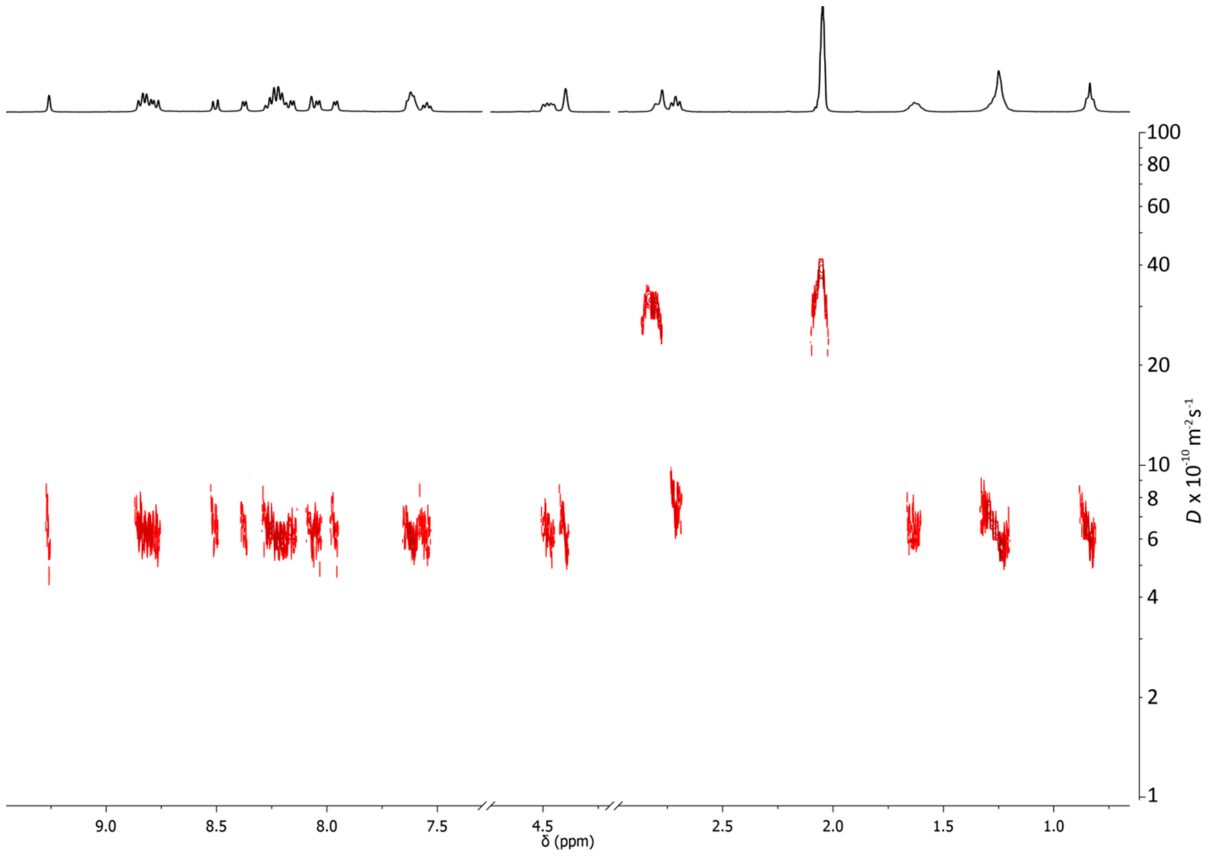

Figure S29: ^{1}H DOSY NMR (d_6-acetone, 500 MHz, 298 K) of **L4Ru**.

5 Electrochemistry

All cyclic voltammetry (CV) experiments were performed in solutions at 20 ˚C (CH_2Cl_2) with a concentration of 1 mM of electroactive analyte and 0.1 M NBu_4PF_6 as the supporting electrolyte. A three-electrode cell was used with Cypress Systems 1.4 mm diameter glassy carbon working, Ag/AgCl reference and platinum wire auxiliary electrodes. Voltammograms were recorded with the aid of a Powerlab/4sp computer-controlled potentiostat. Potentials for all complexes were referenced to the reversible formal potential (taken as E° = 0.00 V) of the $[Fc^*]^{+/0}$ redox couple of decamethylferrocene [2]. In the cases of **L1-4**, scans between -2.0 V and 1.7 V revealed no processes other than the ferrocenyl oxidation.

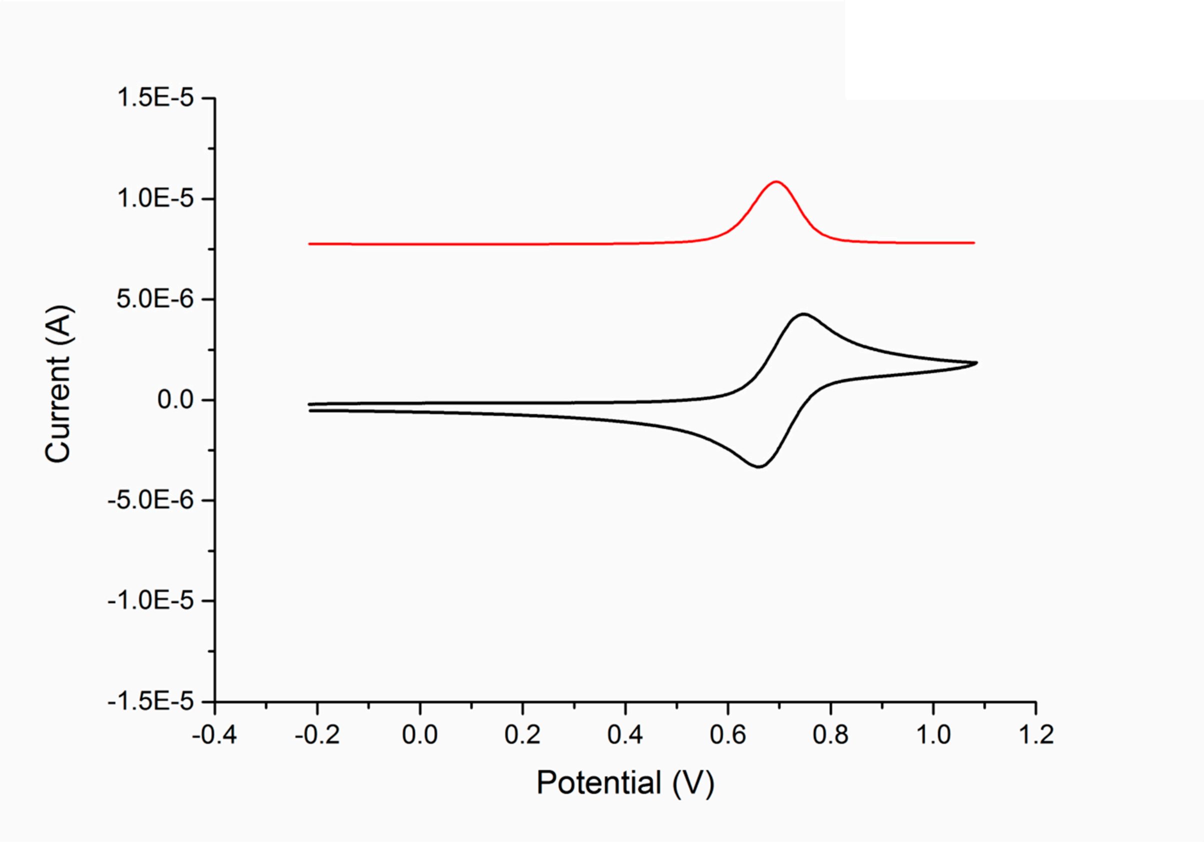

Figure S30: CV of **L1** (black trace) with inset DPV (red trace) (1 mM CH_2Cl_2 solution, 100 mVs^{-1}, Bu_4NPF_6).

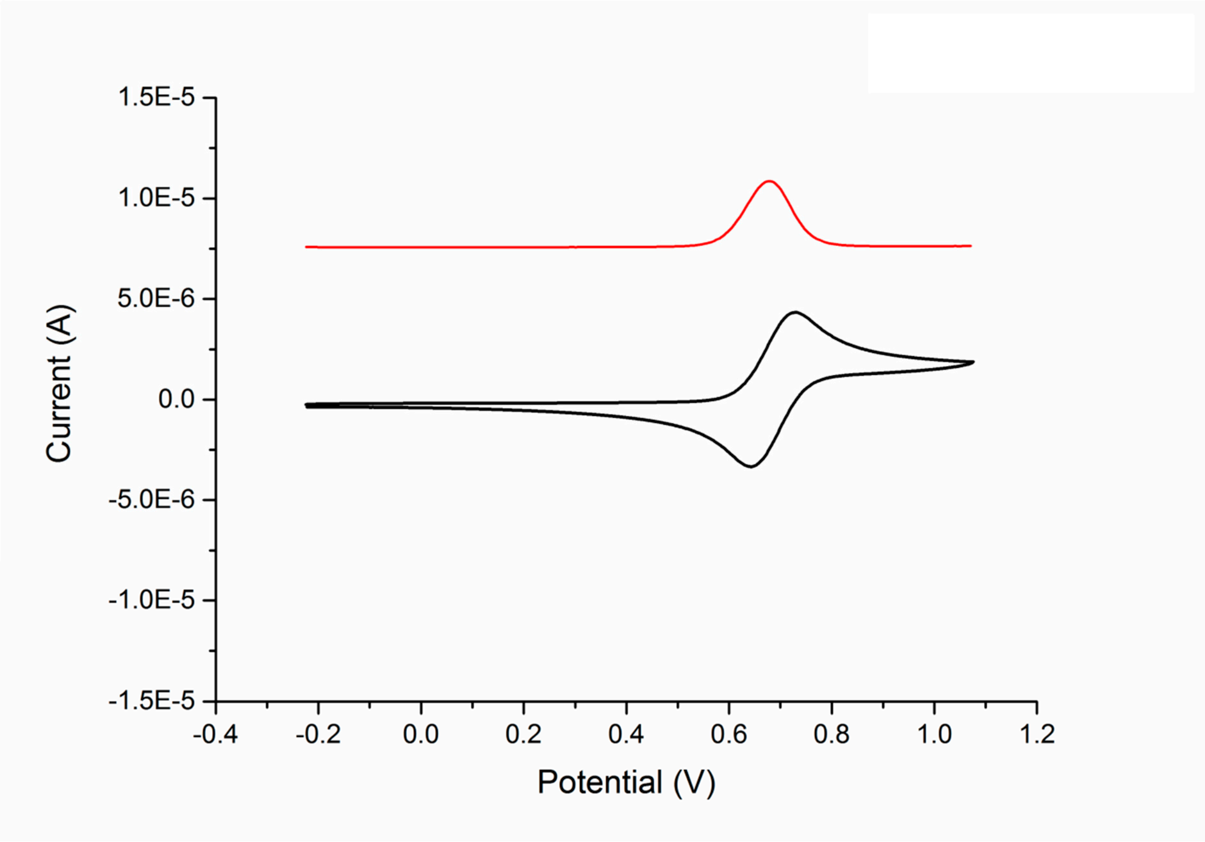

Figure S31: CV of **L2** (black trace) with inset DPV (red trace) (1 mM CH_2Cl_2 solution, 100 mVs^{-1}, Bu_4NPF_6).

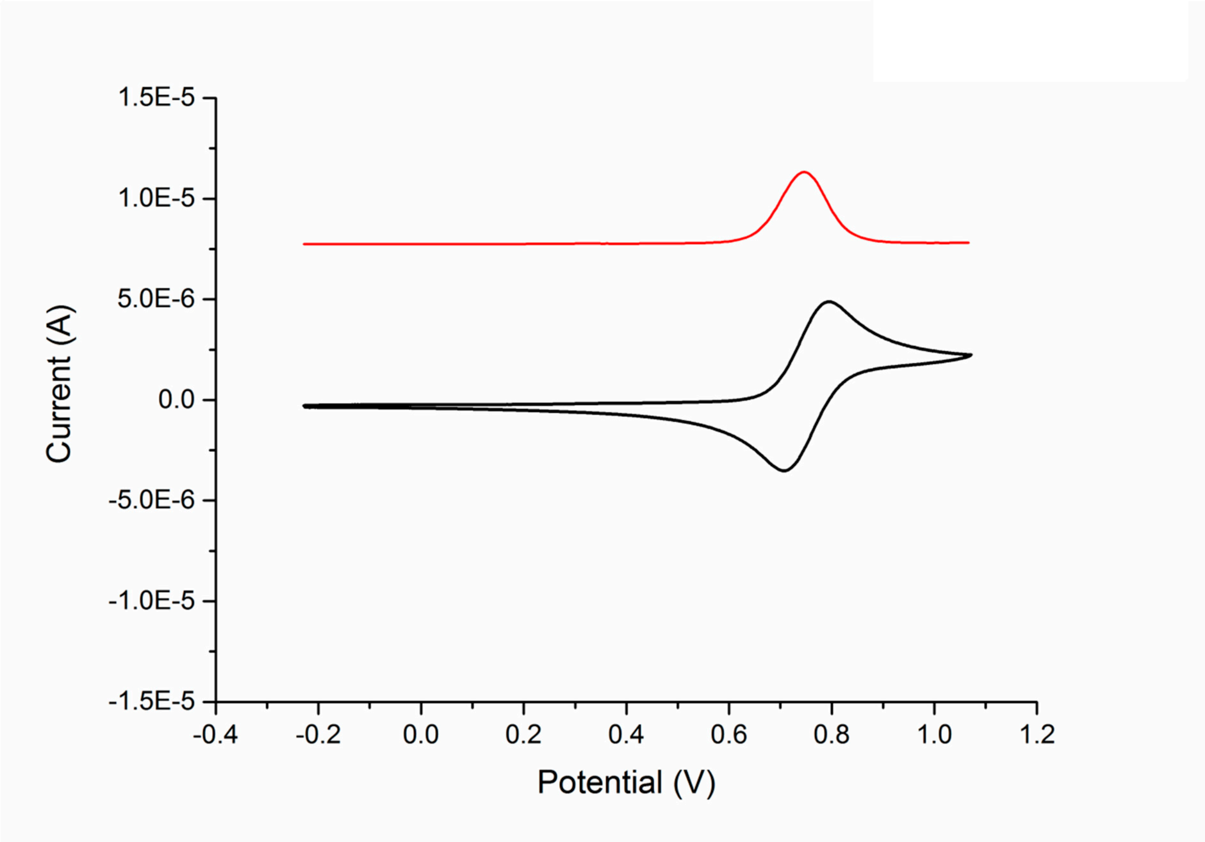

Figure S32: CV of **L3** (black trace) with inset DPV (red trace) (1 mM CH_2Cl_2 solution, 100 mVs^{-1}, Bu_4NPF_6).

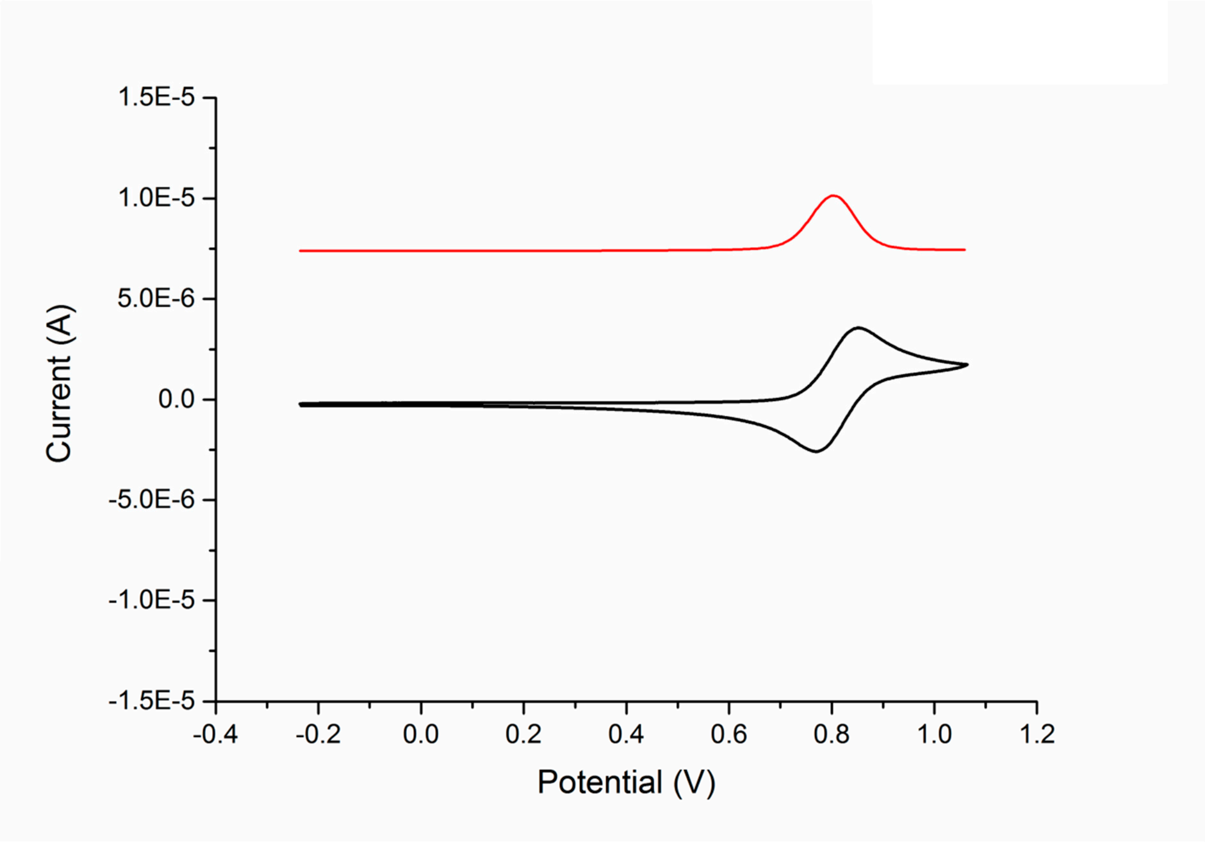

Figure S33: CV of **L4** (black trace) with inset DPV (red trace) (1 mM CH_2Cl_2 solution, 100 mVs^{-1}, Bu_4NPF_6).

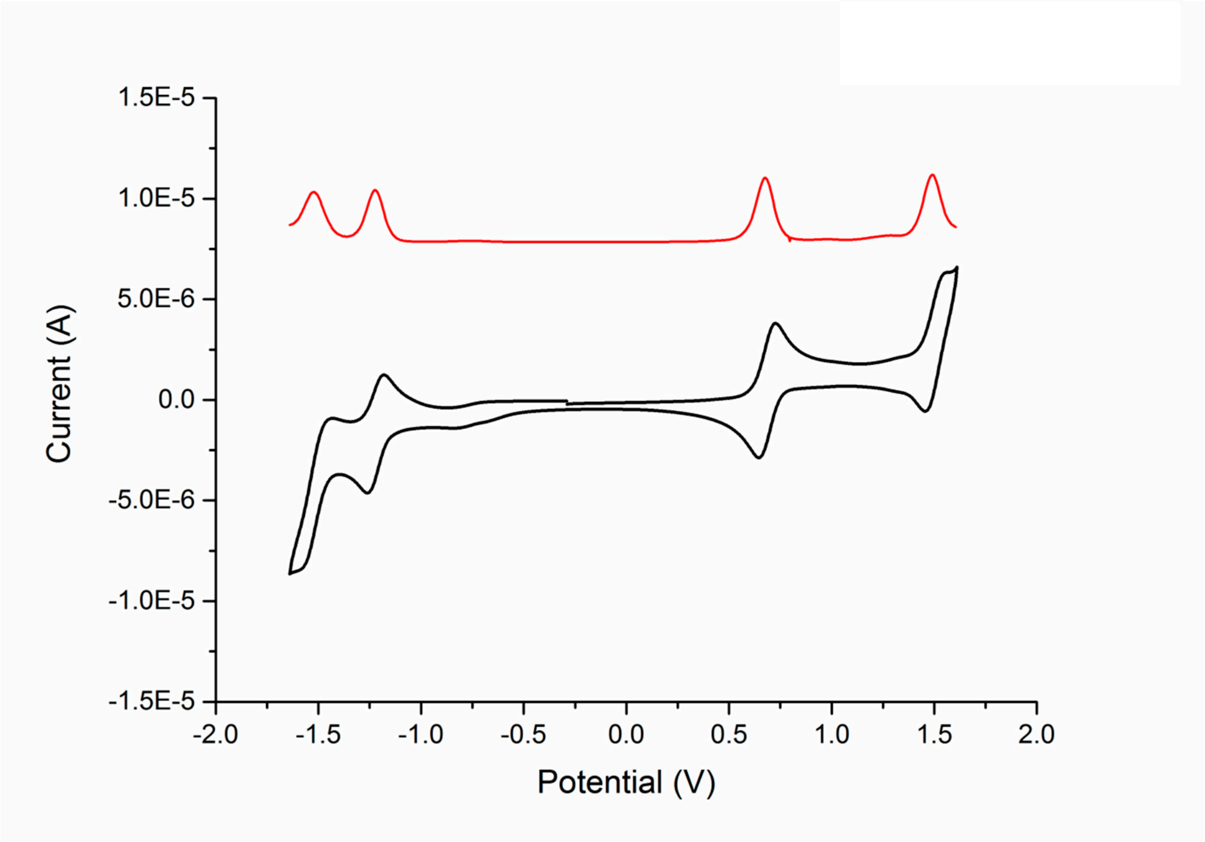

Figure S34: CV of **L1Ru** (black trace) with inset DPV (red trace) (1 mM CH_2Cl_2 solution, 100 mVs^{-1}, Bu_4NPF_6).

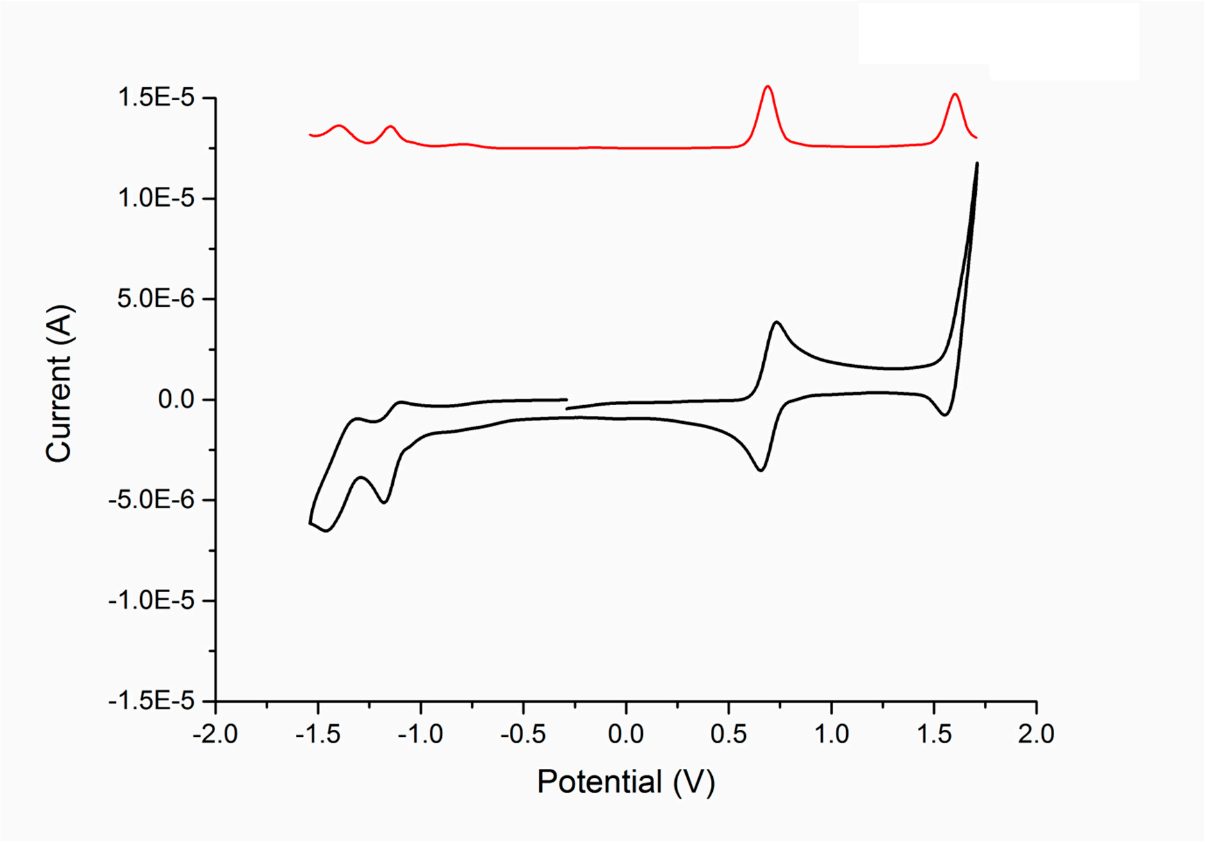

Figure S35: CV of **L2Ru** (black trace) with inset DPV (red trace) (1 mM CH_2Cl_2 solution, 100 mVs^{-1}, Bu_4NPF_6).

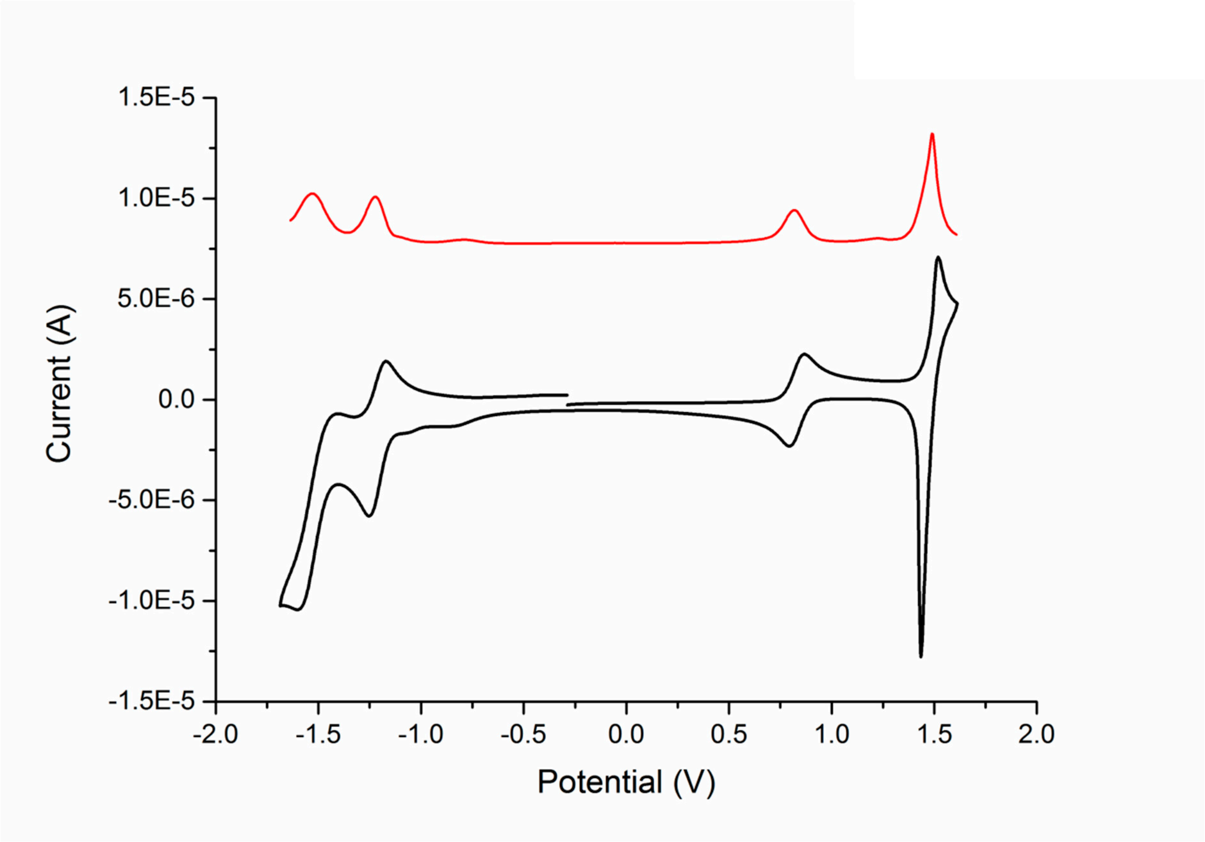

Figure S36: CV of **L3Ru** (black trace) with inset DPV (red trace) (1 mM CH_2Cl_2 solution, 100 mVs^{-1}, Bu_4NPF_6).

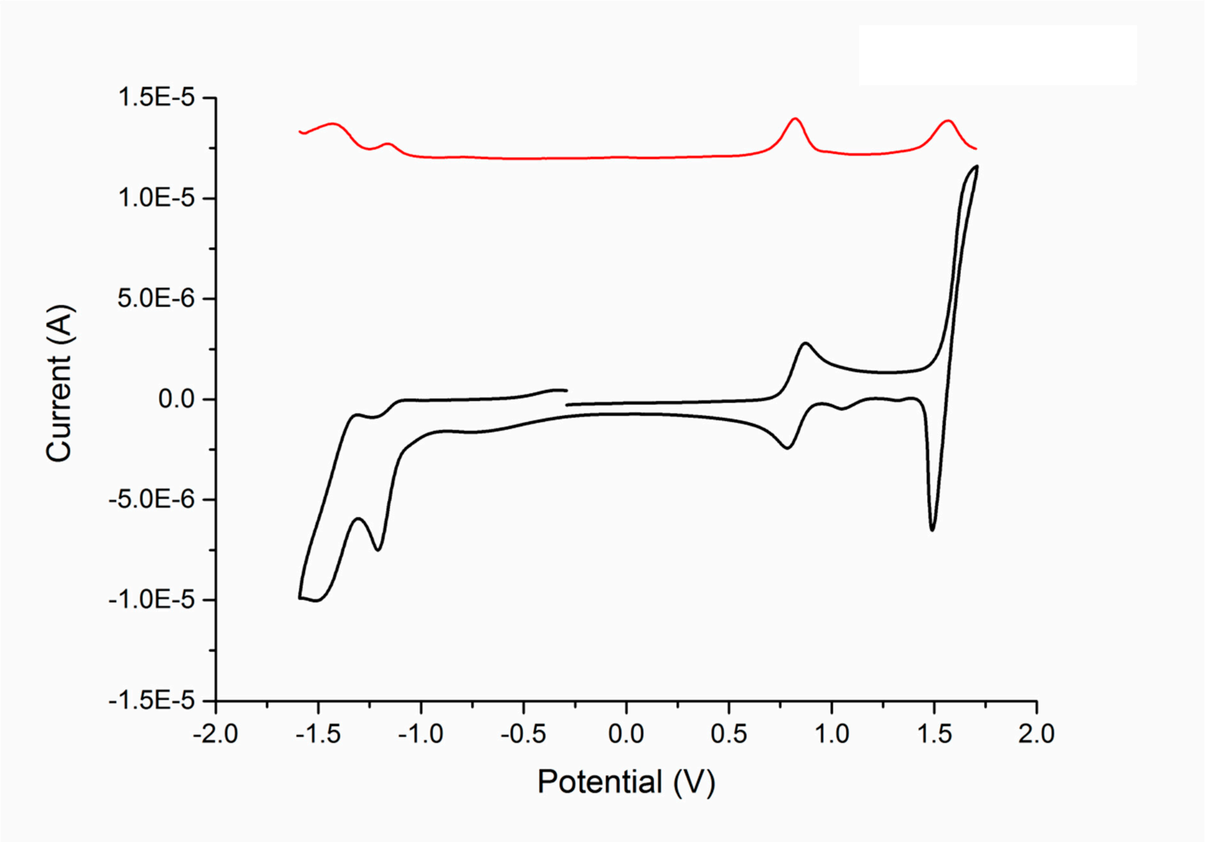

Figure S37: CV of **L4Ru** (black trace) with inset DPV (red trace) (1 mM CH_2Cl_2 solution, 100 mVs^{-1}, Bu_4NPF_6).

6 UV-Visible Spectra

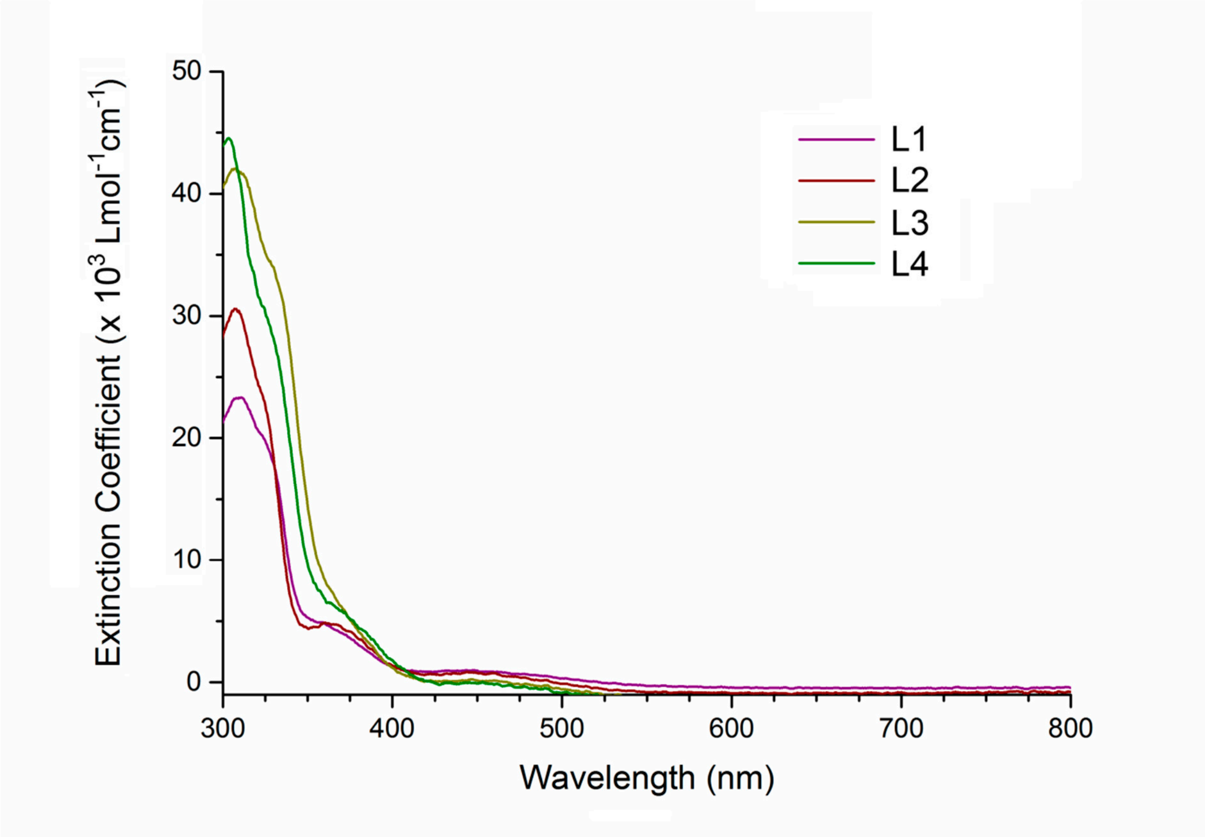

Figure S38: UV-Visible spectra of ligands **L1-4** recorded in dichloromethane solution (~10^{-5} molL^{-1}, 298 K).

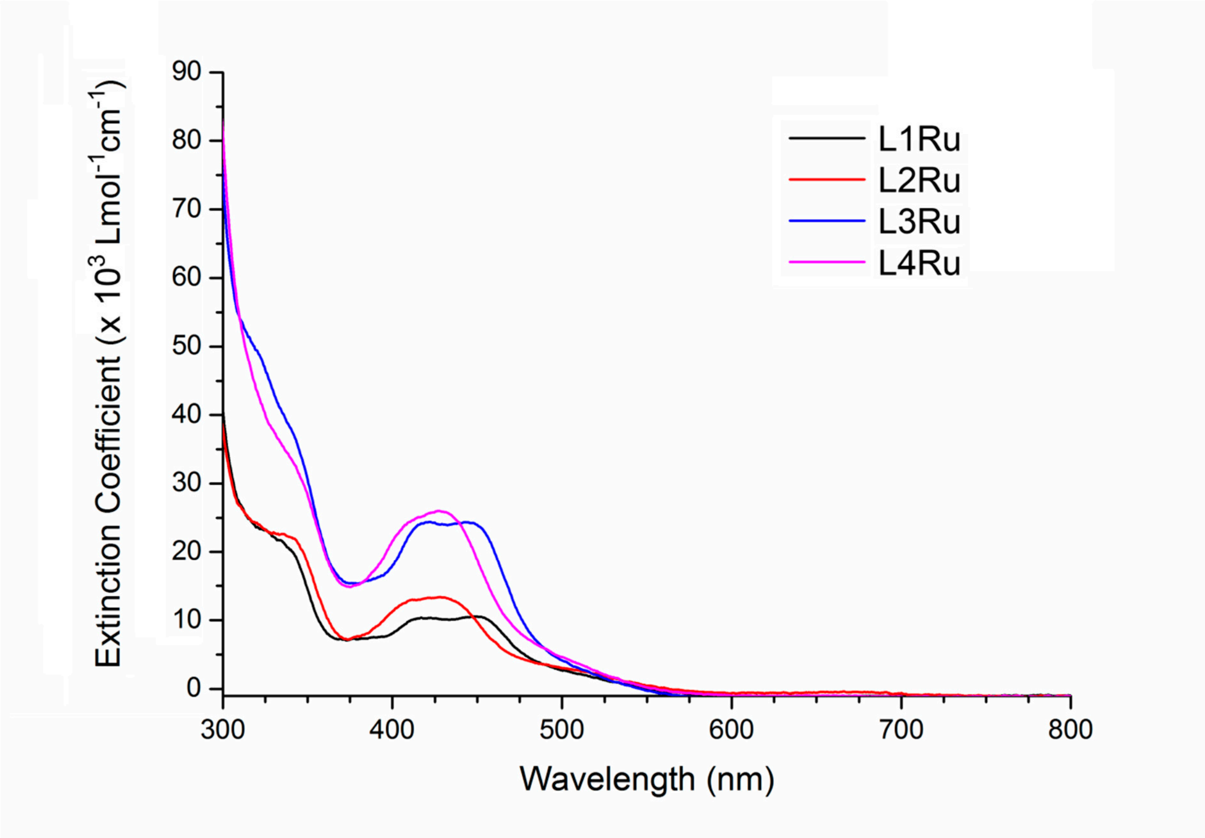

Figure S39: UV-Visible spectra of complexes **L1Ru-L4Ru** recorded in dichloromethane solution (~10^{-5} molL^{-1}, 298 K).

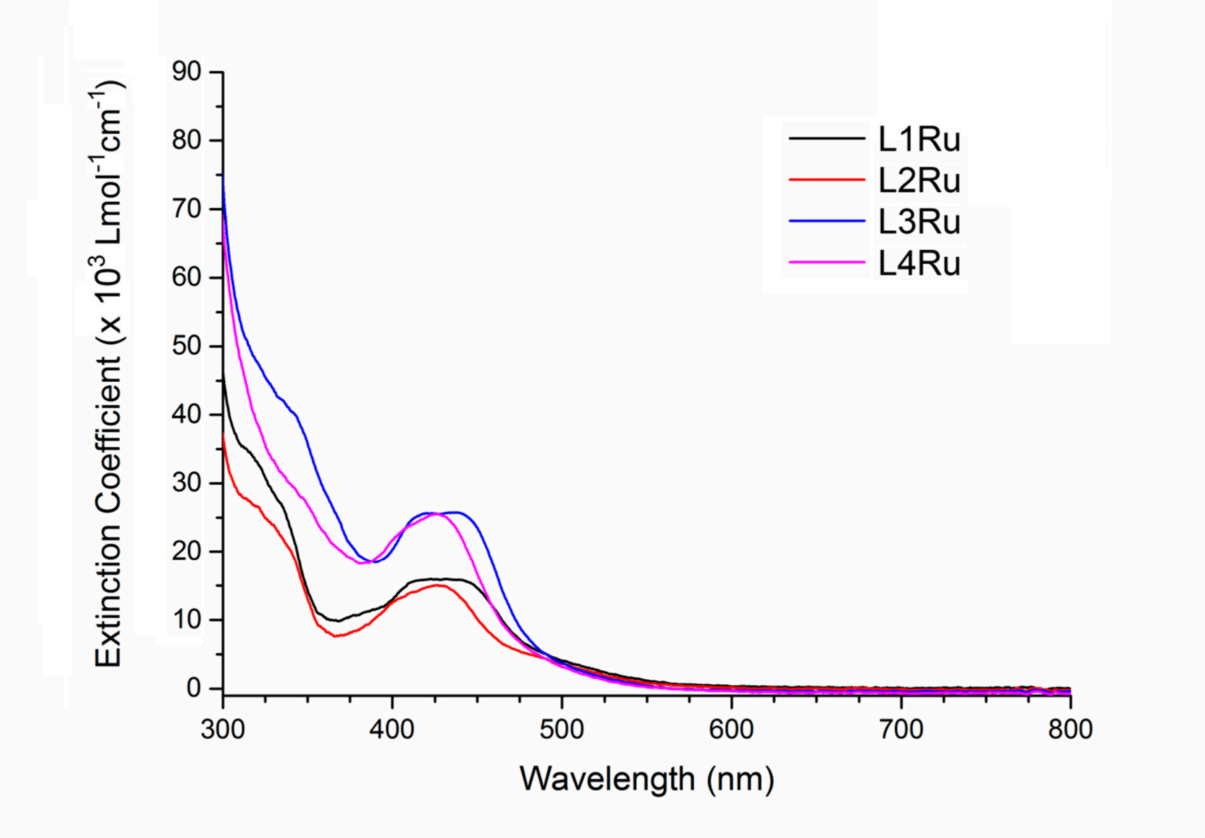

Figure S40: UV-Visible spectra of complexes **L1Ru-L4Ru** recorded in acetonitrile solution (~10^{-5} $molL^{-1}$, 298 K).

7 UV-light Exposure Time Course Experiments ^{1}H NMR Stack Plots

All photochemical experiments were carried out in a custom built UV photochemical reactor (at the University of Otago, Dunedin, NZ), containing four UV-C lamps (Rayonet RPR-2537A, Southern New England Ultraviolet Co., Branford, CT) emitting monochromatic 254 nm radiation, arranged symmetrically around the perimeter of the photoreactor. Samples were suspended in the centre of the UV-bulb arrangement and an in-built fan was used to maintain a constant temperature of 35 °C during irradiation experiments. All samples for photo-ejection experiments were 2 mM in 0.7 mL of [D3]acetonitrile in an NMR tube.

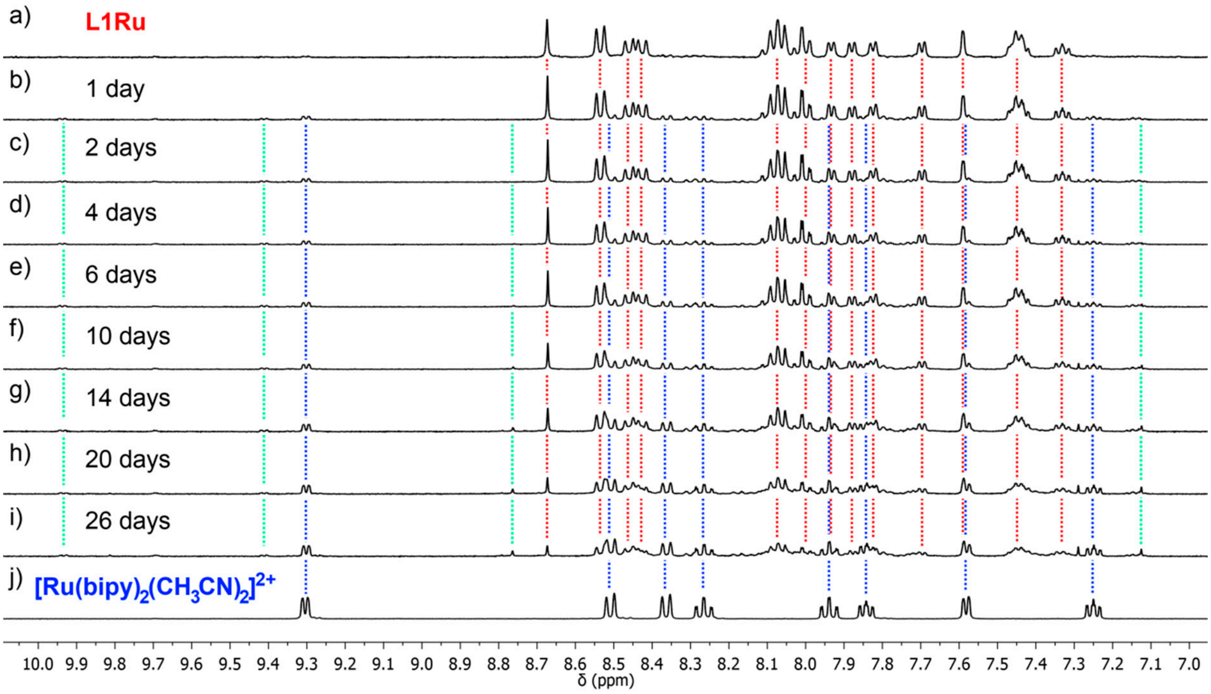

Figure S41: Partial stacked ^{1}H NMR spectra (400 MHz, CD_3CN, 298 K), showing the photo-ejection of $[Ru(bipy)_2(CD_3CN)_2]^{2+}$ (blue dashed lines) from **L1Ru** (red dashed lines) over time under UV (254 nm) irradiation; **a)** ^{1}H NMR of unirradiated **L1Ru**, **b)-i)** selected ^{1}H NMR of **L1Ru** after 1-26 days of UV irradiation, and **j)** ^{1}H NMR of independently synthesised $[Ru(bipy)_2(CH_3CN)_2](BF_4)_2$. Green dashed lines indicate the most visible signals corresponding to intermediate species.

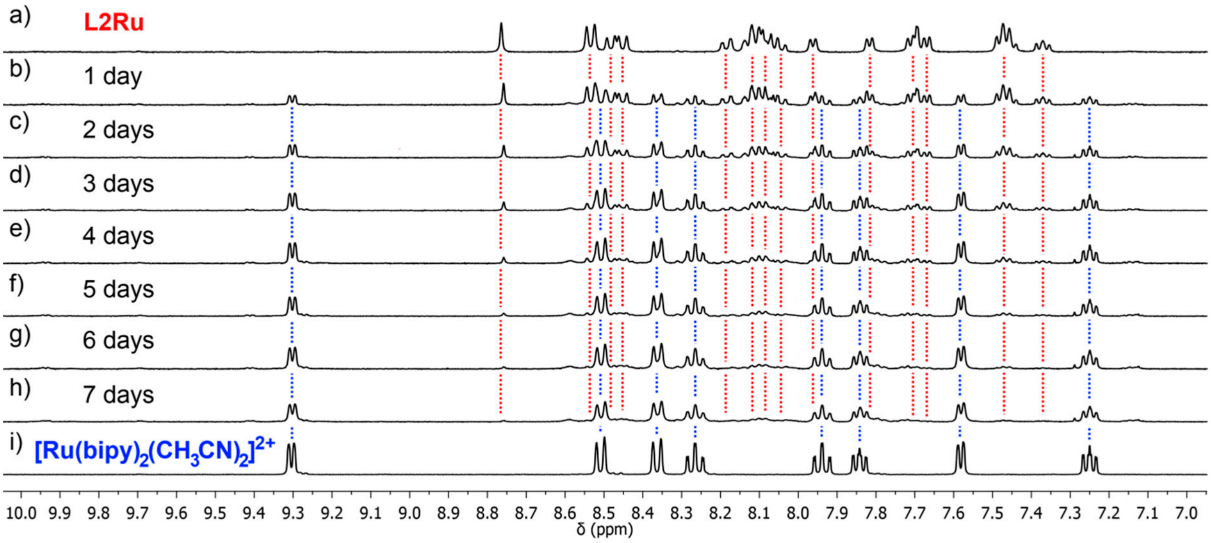

Figure S42: Partial stacked ^{1}H NMR spectra (400 MHz, CD_3CN, 298 K), showing the photo-ejection of $[Ru(bipy)_2(CD_3CN)_2]^{2+}$ (blue dashed lines) from **L2Ru** (red dashed lines) over time under UV (254 nm) irradiation; **a)** ^{1}H NMR of unirradiated **L2Ru**, **b)-h)** ^{1}H NMR of **L2Ru** after 1-7 days of UV irradiation, and **i)** ^{1}H NMR of independently synthesised $[Ru(bipy)_2(CH_3CN)_2](BF_4)_2$.

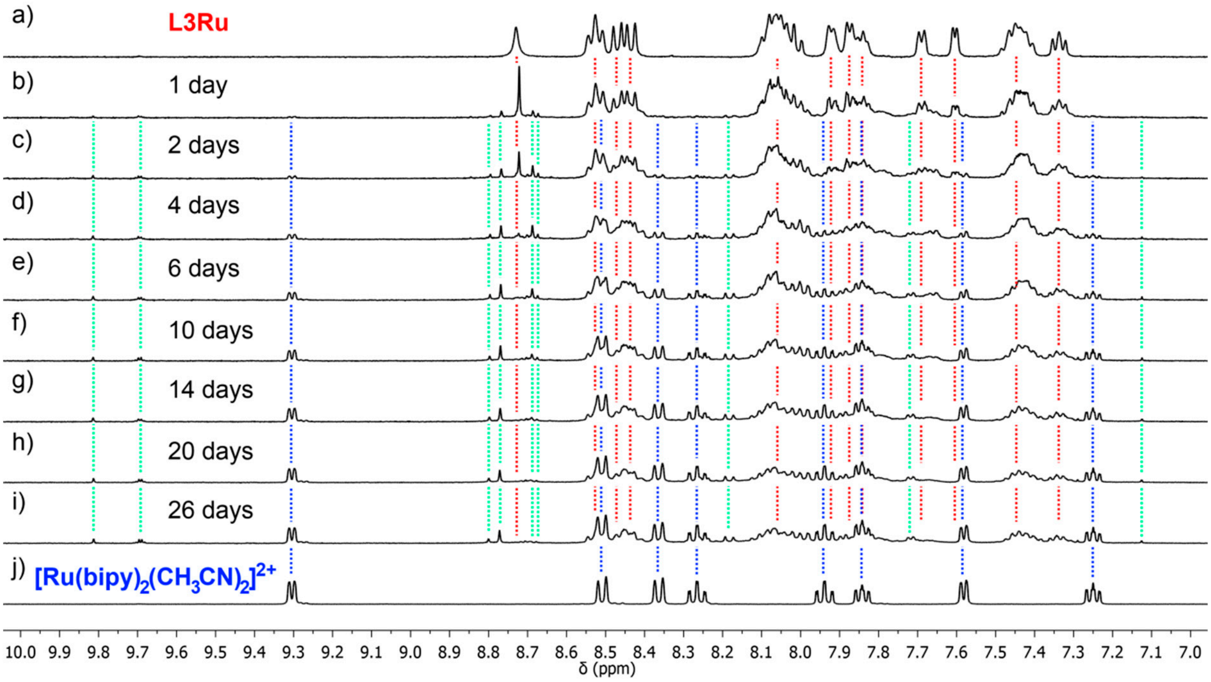

Figure S43: Partial stacked ^{1}H NMR spectra (400 MHz, CD_3CN, 298 K), showing the photo-ejection of $[Ru(bipy)_2(CD_3CN)_2]^{2+}$ (blue dashed lines) from **L3Ru** (red dashed lines) over time under UV (254 nm) irradiation; **a)** ^{1}H NMR of unirradiated **L3Ru**, **b)-i)** selected ^{1}H NMR of **L3Ru** after 1-26 days of UV irradiation, and **j)** ^{1}H NMR of independently synthesised $[Ru(bipy)_2(CH_3CN)_2](BF_4)_2$. Green dashed lines indicate the most visible signals corresponding to intermediate species.

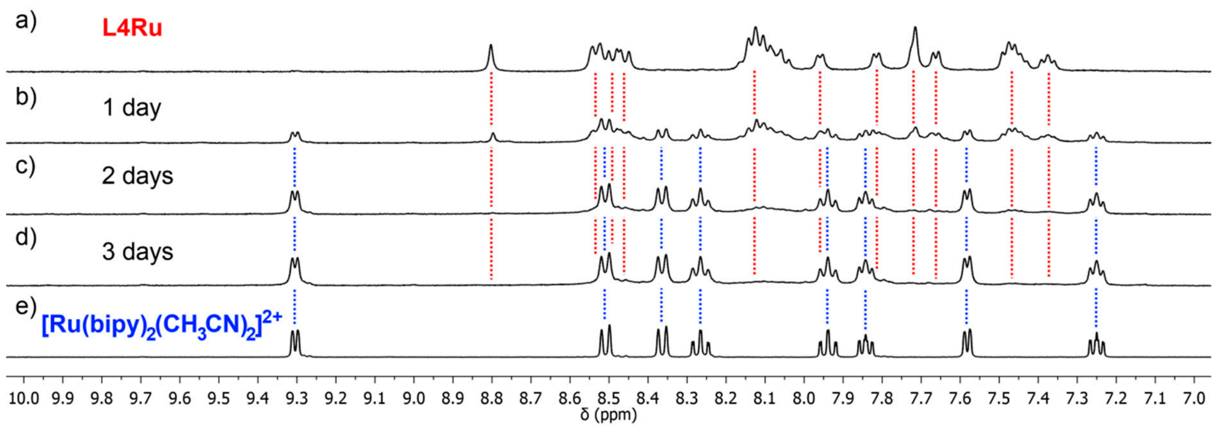

Figure S44: Partial stacked ^{1}H NMR spectra (400 MHz, CD_3CN, 298 K), showing the photo-ejection of $[Ru(bipy)_2(CD_3CN)_2]^{2+}$ (blue dashed lines) from **L4Ru** (red dashed lines) over time under UV (254 nm) irradiation; **a)** ^{1}H NMR of unirradiated **L4Ru**, **b)-d)** ^{1}H NMR of **L4Ru** after 1-3 days of UV irradiation, and **e)** ^{1}H NMR of independently synthesised $[Ru(bipy)_2(CH_3CN)_2](BF_4)_2$.

8 Selected Thermal Re-coordination Experiment Attempt ^{1}H NMR Data

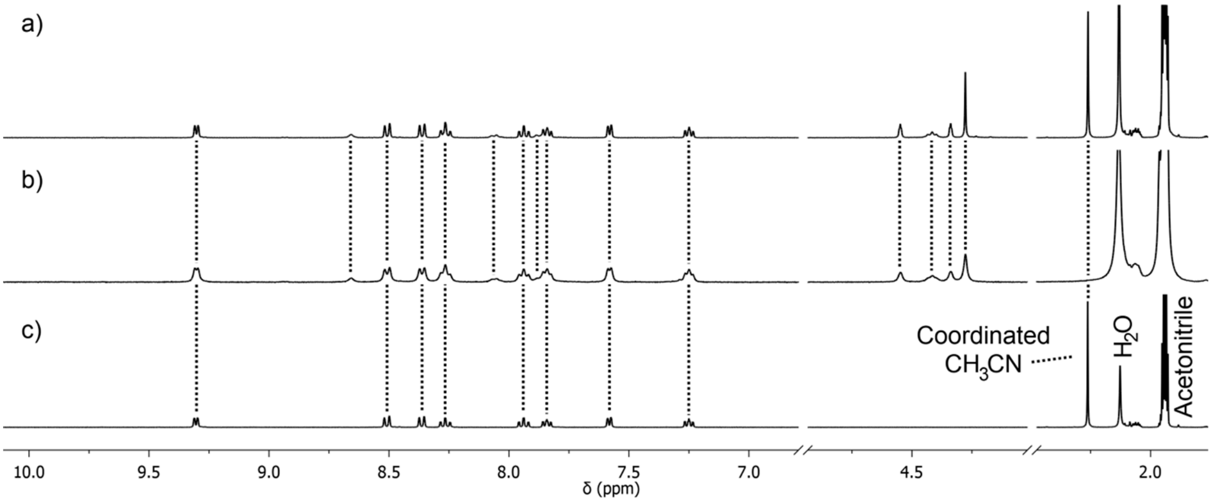

Figure S45: Partial stacked ^{1}H NMR (400 MHz, 298 K, CD_3CN) showing results of attempted coordination of **L1** with $[Ru(bipy)_2(CH_3CN)_2](BF_4)_2$ in CD_3CN; **a)** ^{1}H NMR shortly after combining **L1** and $[Ru(bipy)_2(CH_3CN)_2](BF_4)_2$ (1:1 eq.) in CD_3CN; **b)** ^{1}H NMR of the same mixture after heating at 80 ˚C for 24 hours in the dark; **c)** ^{1}H NMR of $[Ru(bipy)_2(CH_3CN)_2]](BF_4)_2$ in CD_3CN for comparison.

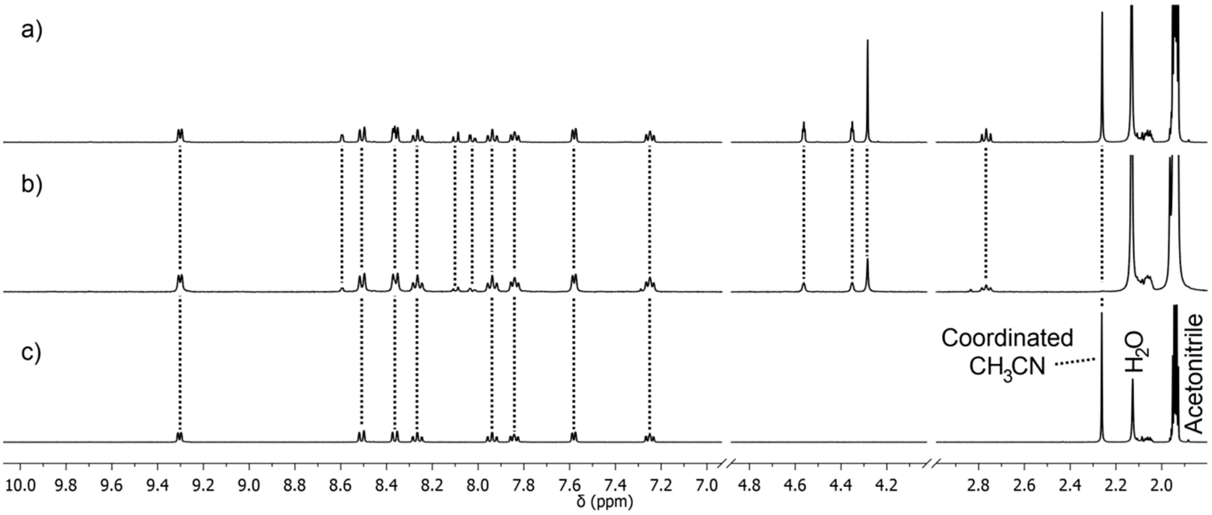

Figure S46: Partial stacked ^{1}H NMR (400 MHz, 298 K, CD_3CN) showing results of attempted coordination of **L2** with $[Ru(bipy)_2(CH_3CN)_2]](BF_4)_2$ in CD_3CN; **a)** ^{1}H NMR shortly after combining **L2** and $[Ru(bipy)_2(CH_3CN)_2](BF_4)_2$ (1:1 eq.) in CD_3CN; **b)** ^{1}H NMR of the same mixture after heating at 80 ˚C for 24 hours in the dark; **c)** ^{1}H NMR of $[Ru(bipy)_2(CH_3CN)_2](BF_4)_2$ in CD_3CN for comparison.

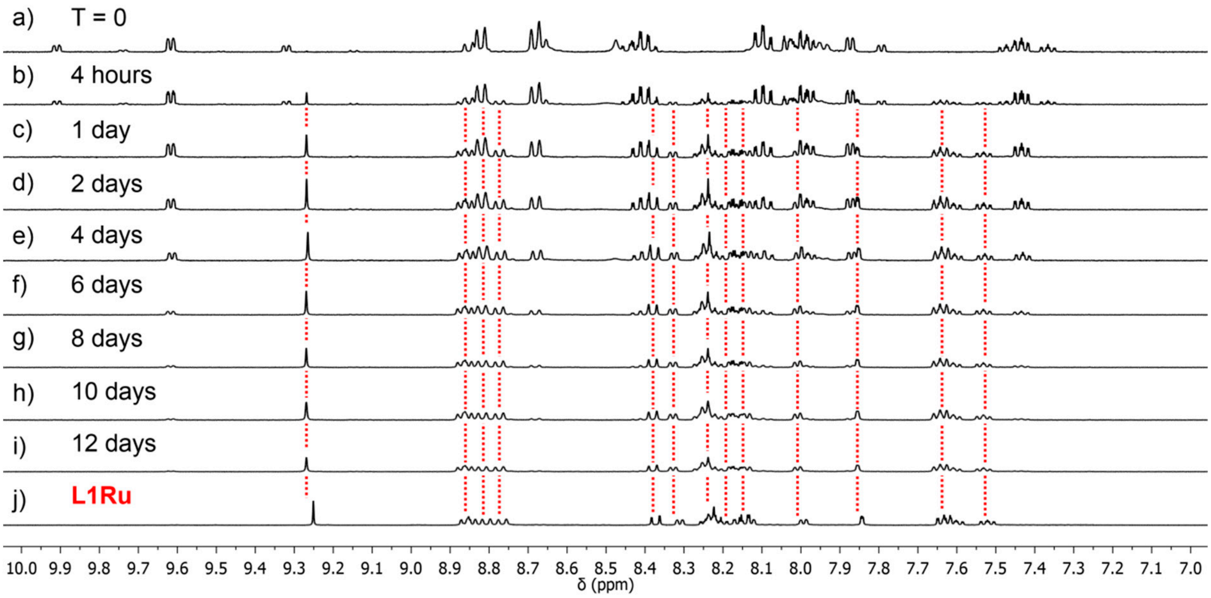

Figure S47: Partial stacked ^{1}H NMR (400 MHz, 298 K, d_6-acetone) showing results of coordination of **L1** with $[Ru(bipy)_2(CH_3CN)_2]](BF_4)_2$ in [D6]acetone solution upon heating (65 ˚C); **a)** Partial ^{1}H NMR of the mixture of **L1** and $[Ru(bipy)_2(CH_3CN)_2]](BF_4)_2$ recorded immediately after combining in [D6]acetone (note the complicated set of signals are due to partial displacement of coordinated CH_3CN ligands by acetone; **b)** after heating for four hours; **c)** after heating for one day; **d)** after heating for two days; **e)** after heating for four days; **f)** after heating for six days; **g)** after heating for eight days; **h)** after heating for 10 days; **i)** after heating for 12 days; **j)** spectrum of **L1Ru**.

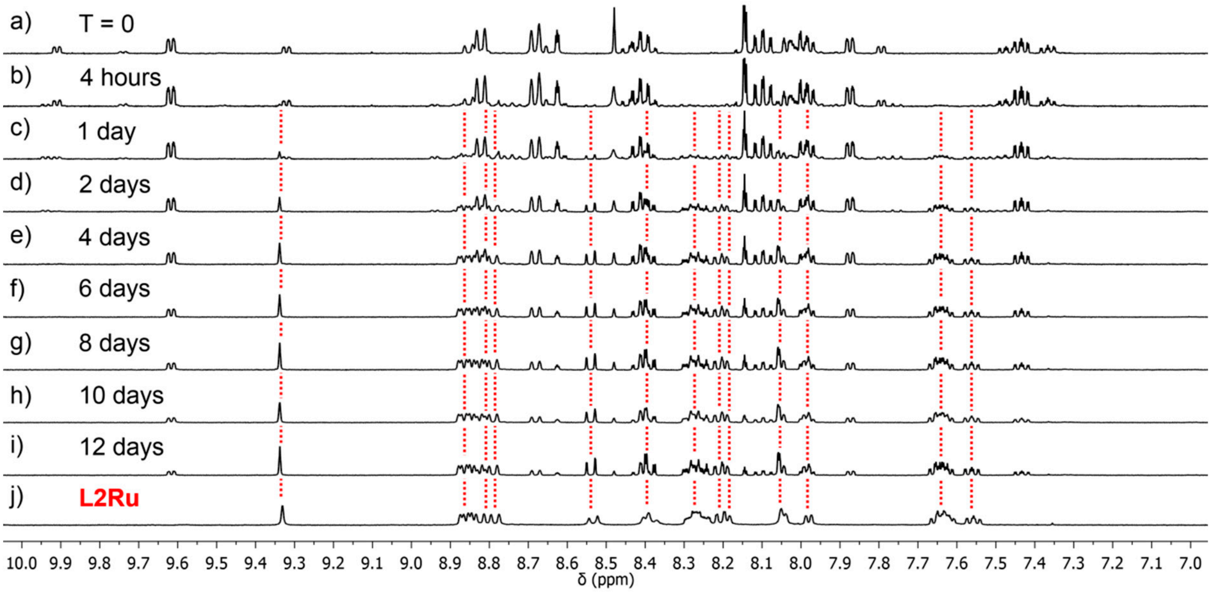

Figure S48: Partial stacked ^{1}H NMR (400 MHz, 298 K, d_6-acetone) showing results of coordination of **L2** with $[Ru(bipy)_2(CH_3CN)_2]](BF_4)_2$ in [D6]acetone solution upon heating (65 ˚C); **a)** Partial ^{1}H NMR of the mixture of **L2** and $[Ru(bipy)_2(CH_3CN)_2]](BF_4)_2$ recorded immediately after combining in [D6]acetone (note the complicated set of signals are due to partial displacement of coordinated CH_3CN ligands by acetone; **b)** after heating for four hours; **c)** after heating for one day; **d)** after heating for two days; **e)** after heating for four days; **f)** after heating for six days; **g)** after heating for eight days; **h)** after heating for 10 days; **i)** after heating for 12 days; **j)** spectrum of **L2Ru**.

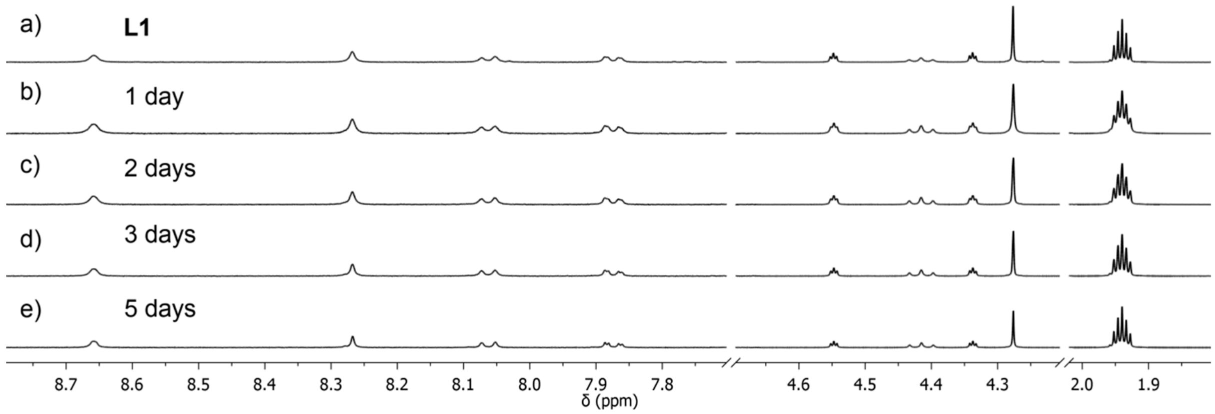

Figure S49: Partial stacked ^{1}H NMR (400 MHz, 298 K, CD_3CN) showing results of UV-irradiation of **L1** in [D3]acetonitrile (2 mM) solution over time. Each spectrum was normalised to the residual acetonitrile solvent signal and showed a decrease in intensity of the signals of **L1** over time, concomitant with loss of the orange colour of **L1** in solution and the formation of brown precipitate in the NMR tube; **a)** Partial ^{1}H NMR of **L1** before irradiation; **b)** after one day of irradiation; **c)** after two days of irradiation; **d)** after three days of irradiation; **e)** after five days of irradiation.

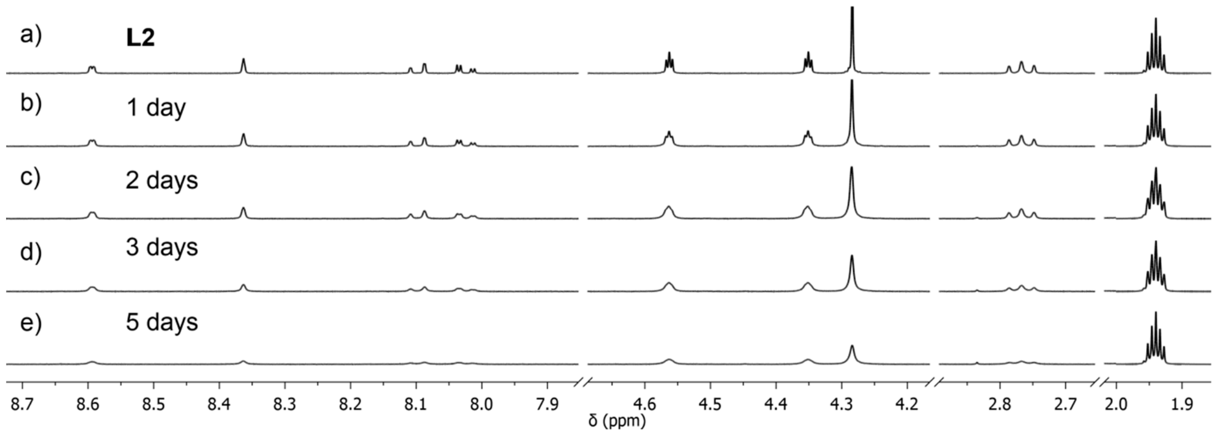

Figure S50: Partial stacked ^{1}H NMR (400 MHz, 298 K, CD_3CN) showing results of UV-irradiation of **L2** in [D3]acetonitrile (2 mM) solution over time. Each spectrum was normalised to the residual acetonitrile solvent signal and showed a decrease in intensity of the signals of **L2** over time, concomitant with loss of the orange colour of **L2** in solution and the formation of brown precipitate in the NMR tube; **a)** Partial ^{1}H NMR of **L2** before irradiation; **b)** after one day of irradiation; **c)** after two days of irradiation; **d)** after three days of irradiation; **e)** after five days of irradiation.

9 X-ray Crystallography Data

9.1 **L2 (*P2₁/c*)**

CCDC #: 1857651. Slow evaporation of a concentrated CH_2Cl_2/methanol solution of **L2**, resulted in the formation of orange rectangular plate crystals of **L2**. X-ray data were collected at 100 K on an Agilent Technologies Supernova system using Cu Kα radiation with exposures over 1.0˚, and data were treated using CrysAlisPro software [3]. The structure was solved using SHELXT and weighted full-matrix refinement on *F*2 was carried out using SHELXL-97 [4] both running within the OLEX2-v1.2.9 package [5]. All non-hydrogen atoms were refined anisotropically. Hydrogen atoms attached to carbons were placed in calculated positions and refined using a riding model. The structure was solved in the primitive space group $P2_1/c$ and refined to an R1 value of 8.1%. The asymmetric unit contains one of the ligand **L2** (figure S51).

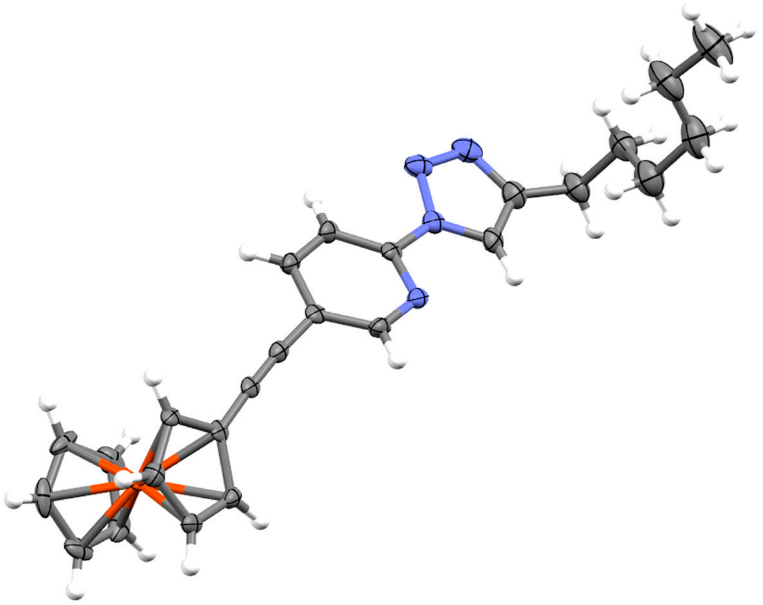

Figure S51: Mercury ellipsoid plot of the asymmetric unit of **L2**. Ellipsoids are shown at the 50% probability level. Colour scheme: carbon grey, hydrogen white, iron orange-red, nitrogen lavender.

Table S2: Crystallographic data for **L2**.

Empirical formula	$C_{25}H_{26}FeN_4$
Formula weight	438.35
Temperature/K	100.01(10)
Crystal system	monoclinic
Space group	$P2_1/c$
a/Å	20.6936(18)
b/Å	5.6569(3)
c/Å	20.5530(13)
α/°	90
β/°	117.608(10)
γ/°	90
Volume/$Å^3$	2132.0(3)
Z	4
Density (calc.) g/cm^3	1.366
Absorption correction $/mm^{-1}$	5.808
F(000)	920.0
Crystal size/mm^3	0.371 × 0.148 × 0.058
Radiation	CuKα (λ = 1.54184)
Theta range for data collection/°	8.61 to 149.78
Index ranges	$-25 \leq h \leq 25$, $-5 \leq k \leq 6$, $-24 \leq l \leq 25$
Reflections collected	20320
Independent reflections	4289 [R_{int} = 0.0713, R_{sigma} = 0.0443]
Data/restraints/parameters	4289/0/272
Goodness-of-fit on F^2	1.094
Final R indexes [I>=2σ (I)]	R_1 = 0.0808, wR_2 = 0.2077
Final R indexes [all data]	R_1 = 0.0874, wR_2 = 0.2132
Largest diff. peak/hole / e $Å^{-3}$	2.60/-0.86

9.2 **L4** ($P\bar{1}$)

CCDC #: 1857652. Slow evaporation of a concentrated CH_2Cl_2/methanol solution of **L4**, resulted in the formation of orange rectangular plate crystals of **L4**. X-ray data were collected at 100 K on an Agilent Technologies Supernova system using Cu Kα radiation with exposures over 1.0˚, and data were treated using CrysAlisPro software [3]. The structure was solved using SHELXS and weighted full-matrix refinement on *F*2 was carried out using SHELXL-97 [4] both running within the OLEX2-1.2 package [5]. All non-hydrogen atoms were refined anisotropically. Hydrogen atoms attached to carbons were placed in calculated positions and refined using a riding model. The structure was solved in the primitive space group $P\bar{1}$ and refined to an R1 value of 4.9%. The asymmetric unit contains one of the ligand **L4** (figure S52).

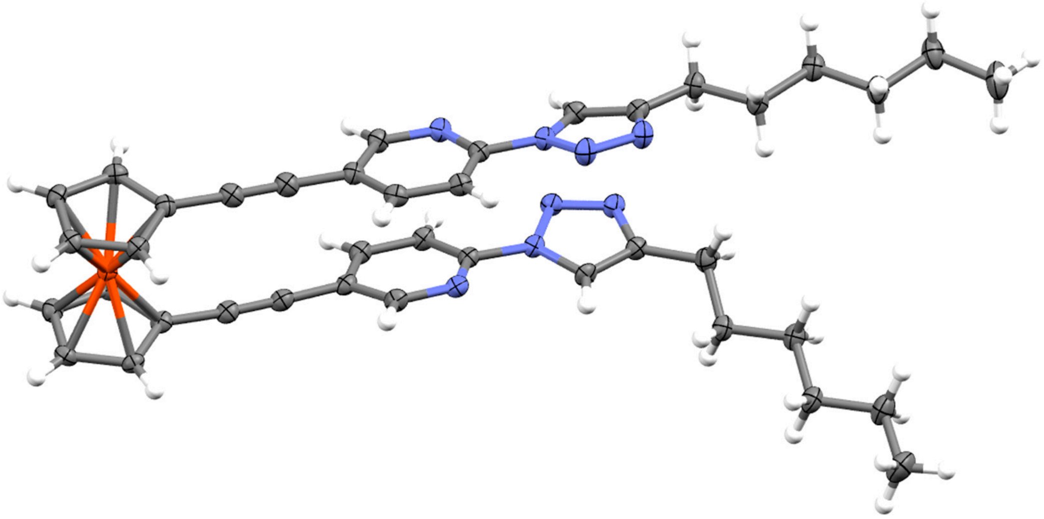

Figure S52: Mercury ellipsoid plot of the asymmetric unit of **L4**. Ellipsoids are shown at the 50% probability level. Colour scheme: carbon grey, hydrogen white, iron orange-red, nitrogen blue.

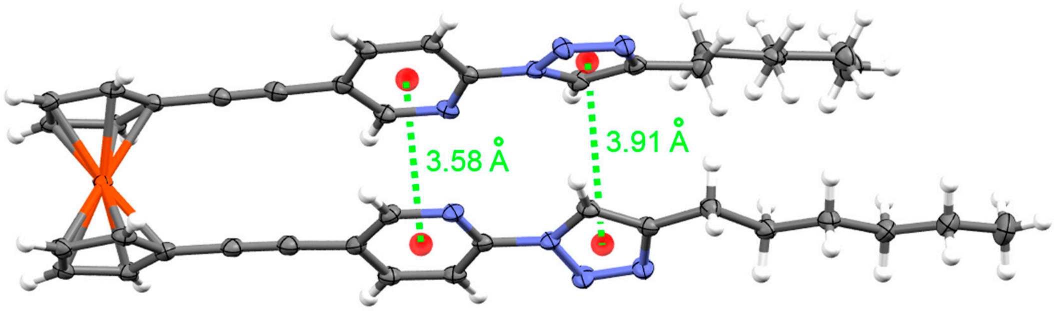

Figure S53: Mercury ellipsoid plot of **L4** showing the centroid –centroid distance of the two pyridine rings.

Table S3: Crystallographic data for **L4**.

Empirical formula	$C_{40}H_{42}FeN_8$
Formula weight	690.66
Temperature/K	100.00(10)
Crystal system	triclinic
Space group	P-1
a/Å	5.6064(2)
b/Å	14.7229(4)
c/Å	21.6649(5)
α/°	75.417(2)
β/°	85.830(2)
γ/°	84.863(2)
Volume/Å^3	1721.38(9)
Z	2
Density (calc.) g/cm^3	1.333
Absorption correction /mm^{-1}	3.835
F(000)	728.0
Crystal size/mm^3	0.218 × 0.086 × 0.054
Radiation	CuKα (λ = 1.54184)
Theta range for data collection/°	6.598 to 148.796
Index ranges	$-6 \le h \le 6$, $-18 \le k \le 18$, $-26 \le l \le 26$
Reflections collected	31319
Independent reflections	6849 [R_{int} = 0.0711, R_{sigma} = 0.0527]
Data/restraints/parameters	6849/0/444
Goodness-of-fit on F^2	1.036
Final R indexes [I>=2σ (I)]	R_1 = 0.0490, wR_2 = 0.1196
Final R indexes [all data]	R_1 = 0.0575, wR_2 = 0.1243
Largest diff. peak/hole / e Å^{-3}	0.44/-0.70

10 Computational Information

Computational modelling was performed with the Gaussian 09 software package [6] using the CAM-B3LYP [7] functional and 6-31G(d) basis set [8] for all atoms besides ruthenium. The ruthenium metal centres used a LanL2DZ effective core potential [9-11] (ECP) to approximate the computationally intensive core electrons. A DMF solvent field was implemented using an integral equation formalism polarisable continuum model (IEF-PCM) self-consistent reaction field (SCRF) [12] with the default solvent parameters provided by Gaussian. This methodology has been effective for modelling Cu(I) bipy-ferrocene systems [13-15] and gave similar results for ruthenium(II) here. Simulated Raman spectra were generated for these optimised structures with GaussSum v2.2 software [16], with a scaling factor of 0.95. Mean absolute deviations (MADs) between experimental and calculated Raman vibrational energies were calculated using well established methods [17] . These were 9 and 11 cm^{-1} for **L3Ru** and **L4Ru**, respectively, and indicate that the ground state structure is well modelled. The open structures (dihedral angle (α) ≈ ±180°) for complexes **L3Ru** and **L4Ru** were optimised and showed no negative frequencies, indicating that a local minimum was achieved. Using these optimised structures a number of intermediate structures were generated by changing the dihedral angle to ±140°, ±110° and ±80° and the energies at these dihedral angles were calculated using single point calculations. Using the open structures as zero points, the energy differences for the various dihedral angles have been evaluated and are shown in Figures S51-58.

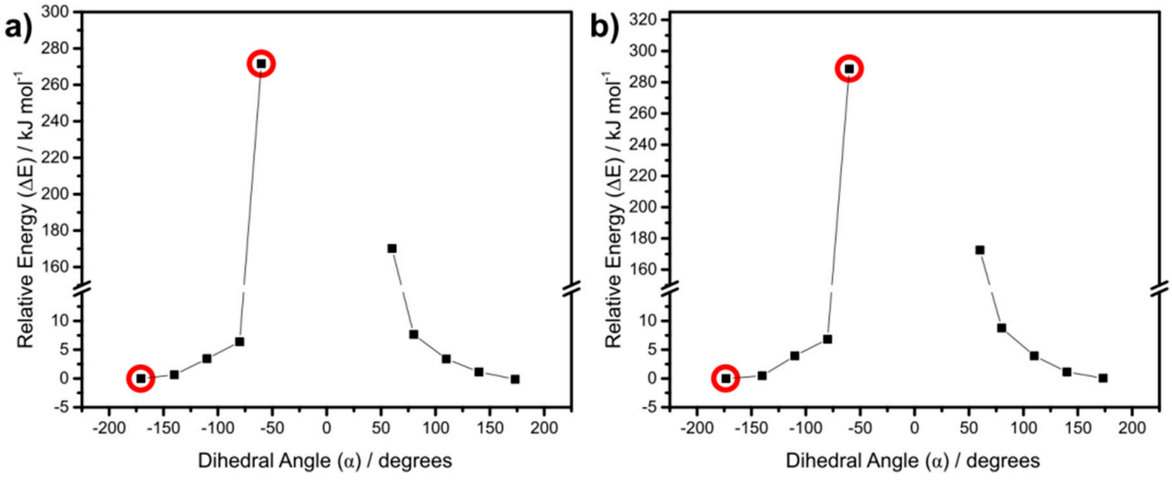

Figure S54: Calculated energies of the various dihedral angles for a) **L3Ru** and b) **L4Ru**. The structures circled in red are shown in Table S3 for reference.

Raman Details

FT-Raman spectra were collected on powder samples using a Bruker MultiRam spectrometer. The excitation source was a Nd:YAG laser with an excitation wavelength of 1064 nm. Raman photons were measured with a liquid nitrogen-cooled D418T germanium detector. Spectra were collected with 128-256 scans, with laser power of 5-50 mW and spectral resolution of 4 cm^{-1}.

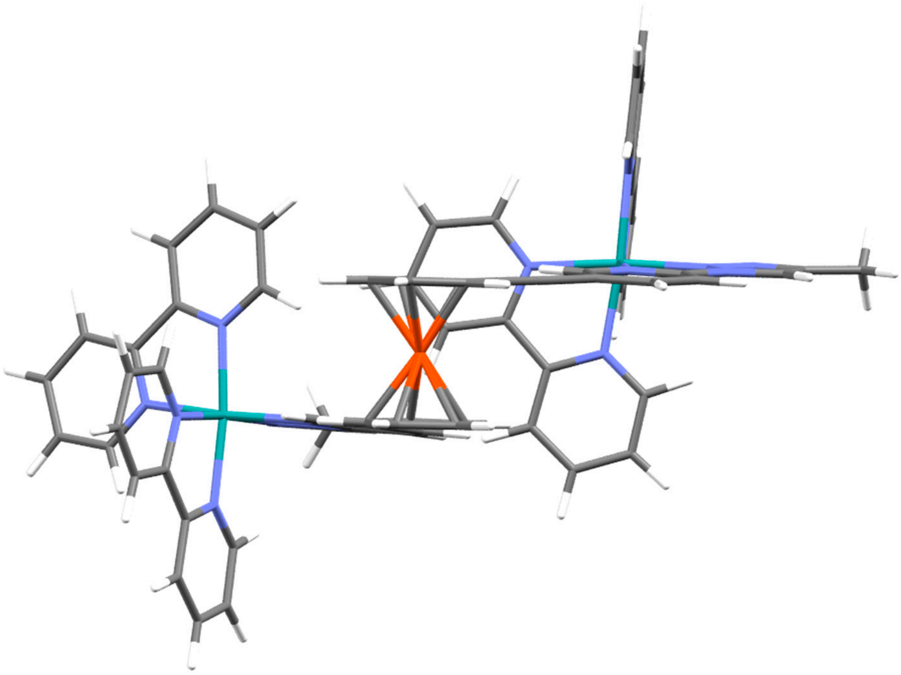

Figure S55: Side view of calculated structure of (Δ,Δ)-**L3Ru** with dihedral = 80˚.

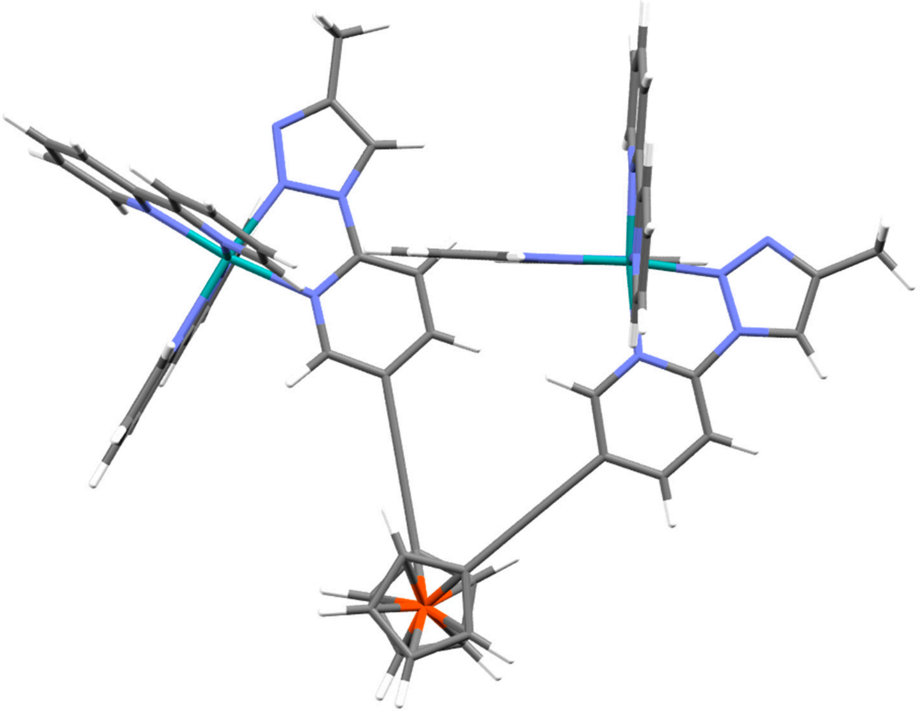

Figure S56: Top view of calculated structure of (Δ,Δ)-**L3Ru** with dihedral = 80˚.

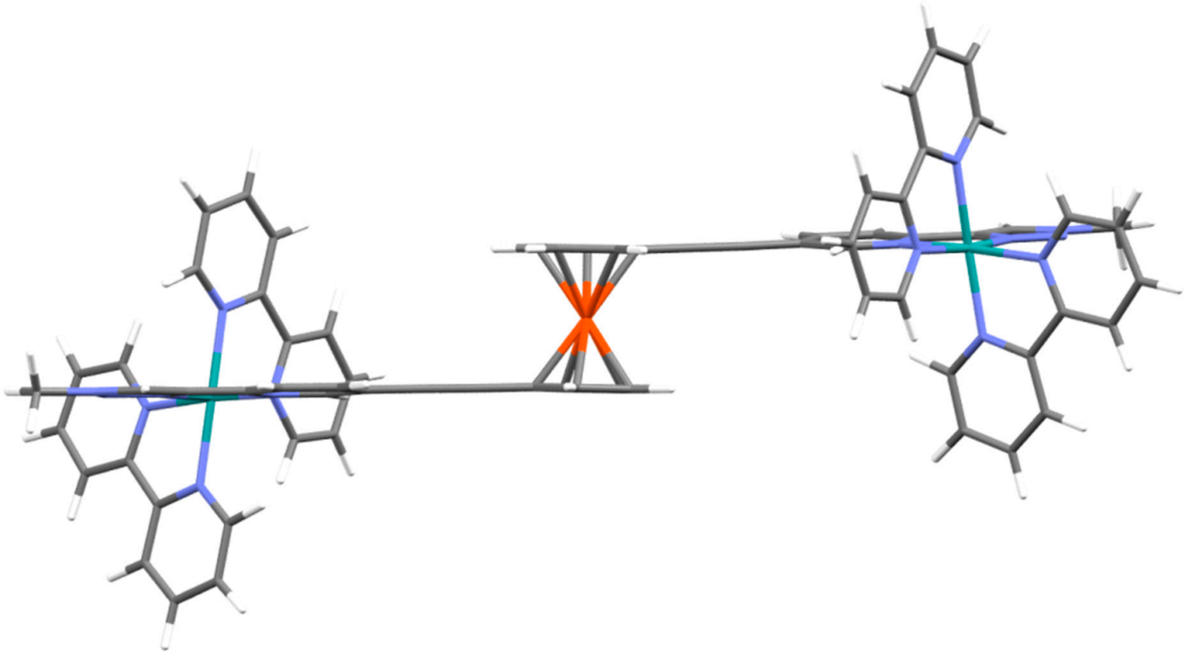

Figure S57: Side view of optimised structure of (Δ,Δ)-**L3Ru** with dihedral ≈ 170˚.

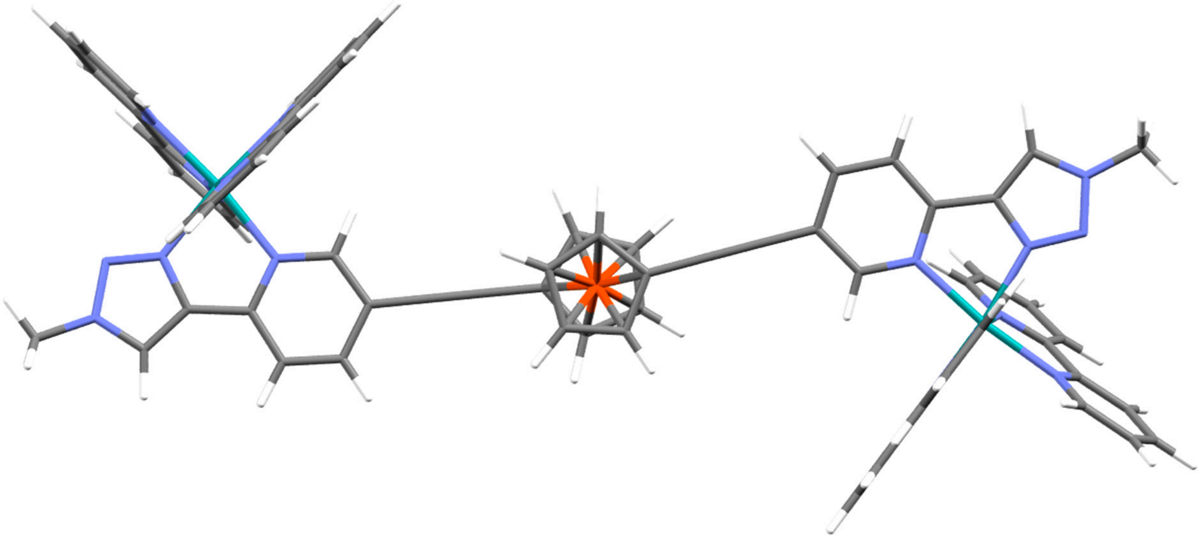

Figure S58: Top view of optimised structure of (Δ,Δ)-**L3Ru** with dihedral ≈ 170˚.

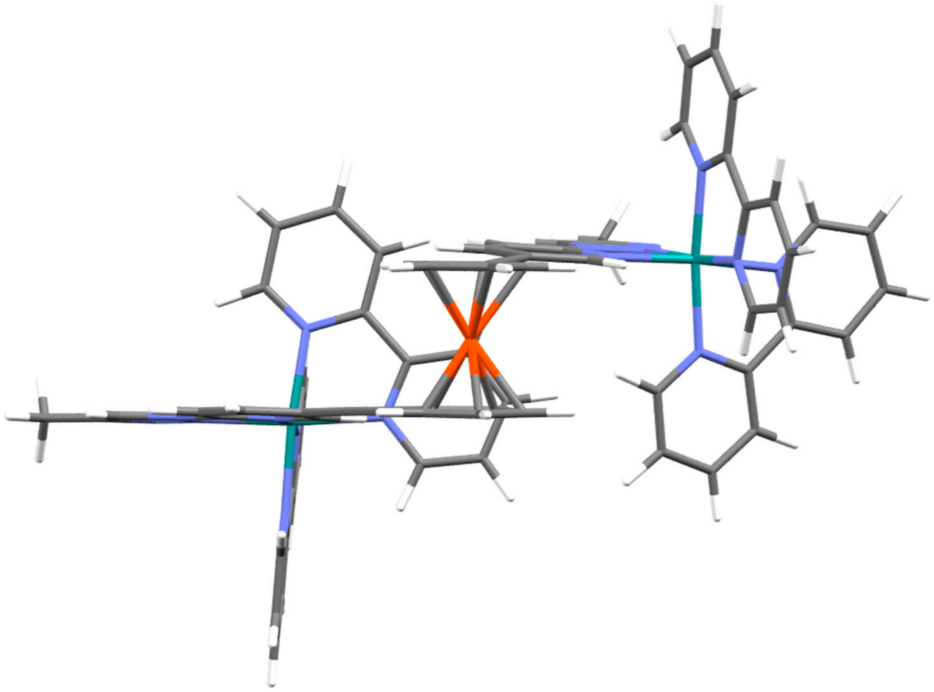

Figure S59: Side view of calculated structure of (Δ,Δ)-**L4Ru** with dihedral = 80˚.

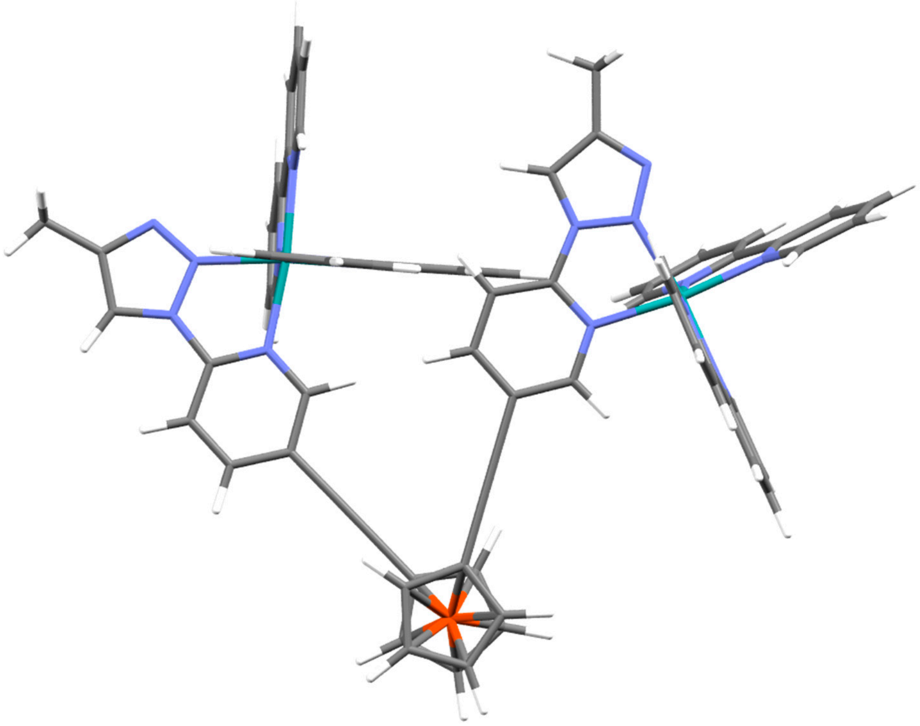

Figure S60: Top view of calculated structure of (Δ,Δ)-**L4Ru** with dihedral = 80˚.

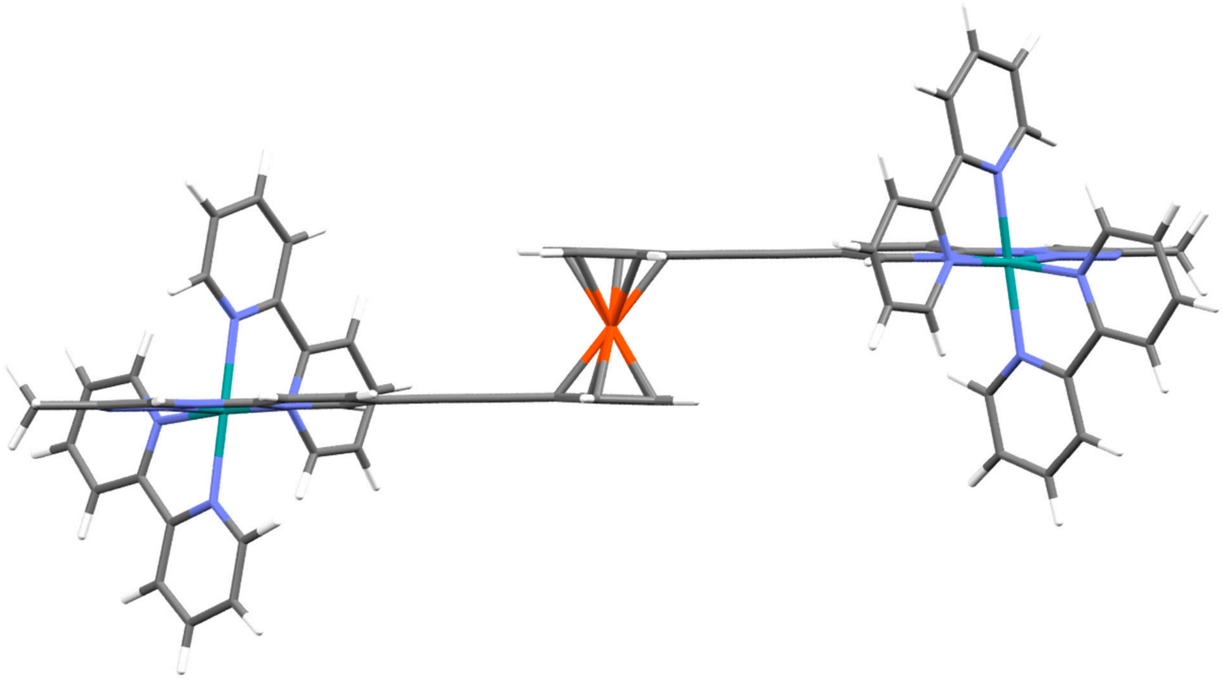

Figure S61: Side view of optimised structure of (Δ,Δ)-**L4Ru** with dihedral ≈ 173˚.

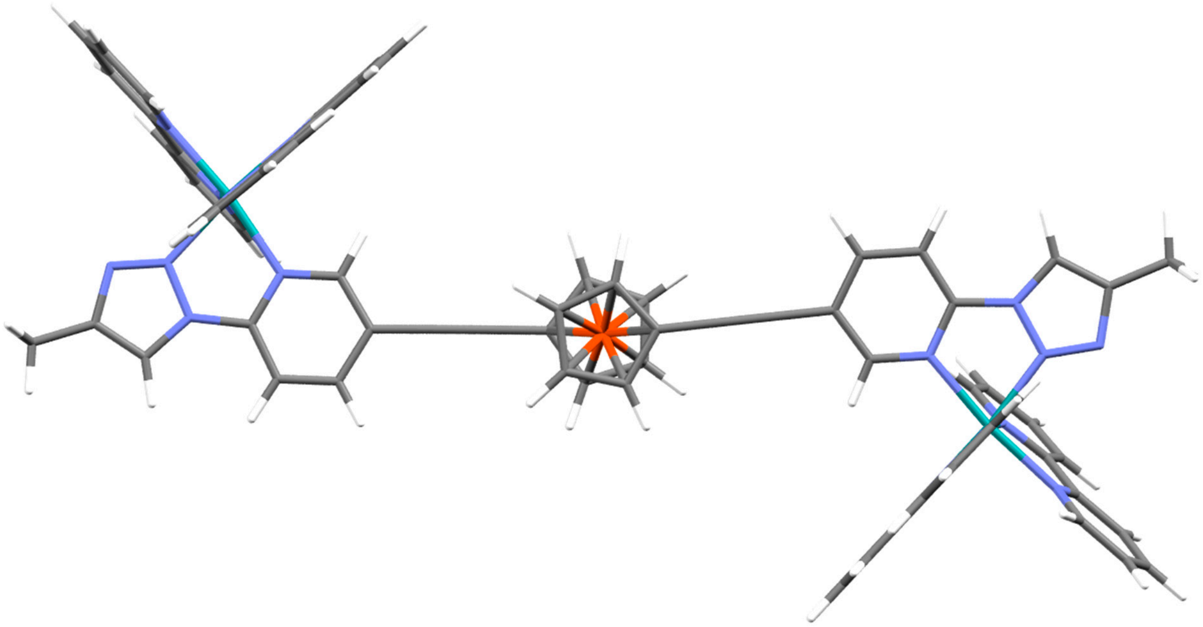

Figure S62: Top view of optimised structure of (Δ,Δ)-**L4Ru** with dihedral ≈ 173˚.

11 References

1. Findlay, J.A.; McAdam, C.J.; Sutton, J.J.; Preston, D.; Gordon, K.C.; Crowley, J.D. Metallosupramolecular architectures formed with ferrocene-linked bis-bidentate ligands: Synthesis, structures, and electrochemical studies. *Inorg. Chem.* **2018**, *57*, 3602-3614.
2. Noviandri, I.; Brown, K.N.; Fleming, D.S.; Gulyas, P.T.; Lay, P.A.; Masters, A.F.; Phillips, L. The decamethylferrocenium/decamethylferrocene redox couple: A superior redox standard to the ferrocenium/ferrocene redox couple for studying solvent effects on the thermodynamics of electron transfer. *J. Phys. Chem. B* **1999**, *103*, 6713-6722.
3. CrysAlisPRO. Oxford Diffraction /Agilent Technologies UK Ltd, Yarnton, England.
4. Sheldrick, G. *Acta Cryst.* **2008**, *A64*, 112-122.
5. Dolomanov, O.V.; Bourhis, L.J.; Gildea, R.J.; Howard, J.A.K.; Puschmann, H. Olex2: A complete structure solution, refinement and analysis program. *J. Appl. Crystallogr.* **2009**, *42*, 339-341.
6. M. J. Frisch, G.W.T., H. B. Schlegel, G. E. Scuseria, M. A. Robb, J. R. Cheeseman, G. Scalmani, V. Barone, G. A. Petersson, H. Nakatsuji, X. Li, M. Caricato, A. Marenich, J. Bloino, B. G. Janesko, R. Gomperts, B. Mennucci, H. P. Hratchian, J. V. Ortiz, A. F. Izmaylov, J. L. Sonnenberg, D. Williams-Young, F. Ding, F. Lipparini, F. Egidi, J. Goings, B. Peng, A. Petrone, T. Henderson, D. Ranasinghe, V. G. Zakrzewski, J. Gao, N. Rega, G. Zheng, W. Liang, M. Hada, M. Ehara, K. Toyota, R. Fukuda, J. Hasegawa, M. Ishida, T. Nakajima, Y. Honda, O. Kitao, H. Nakai, T. Vreven, K. Throssell, J. A. Montgomery, Jr., J. E. Peralta, F. Ogliaro, M. Bearpark, J. J. Heyd, E. Brothers, K. N. Kudin, V. N. Staroverov, T. Keith, R. Kobayashi, J. Normand, K. Raghavachari, A. Rendell, J. C. Burant, S. S. Iyengar, J. Tomasi, M. Cossi, J. M. Millam, M. Klene, C. Adamo, R. Cammi, J. W. Ochterski, R. L. Martin, K. Morokuma, O. Farkas, J. B. Foresman, and D. J. Fox *Gaussian 09*, D.01; Gaussian, Inc.: Wallingford CT, 2016.
7. Yanai, T.; Tew, D.P.; Handy, N.C. A new hybrid exchange–correlation functional using the coulomb-attenuating method (cam-b3lyp). *Chem. Phys. Lett.* **2004**, *393*, 51-57.
8. Hariharan, P.C.; Pople, J.A. The influence of polarization functions on molecular orbital hydrogenation energies. *Theor Chim Acta.* **1973**, *28*, 213-222.
9. Hay, P.J.; Wadt, W.R. Ab initio effective core potentials for molecular calculations. Potentials for the transition metal atoms Sc to Hg. *J Chem Phys.* **1985**, *82*, 270-283.
10. Hay, P.J.; Wadt, W.R. Ab initio effective core potentials for molecular calculations. Potentials for main group elements Na to Bi. *J Chem Phys.* **1985**, *82*, 284-298.
11. Hay, P.J.; Wadt, W.R. Ab initio effective core potentials for molecular calculations. Potentials for K to Au including the outermost core orbitals. *J Chem Phys.* **1985**, *82*, 299-310.
12. Tomasi, J.; Mennucci, B.; Cammi, R. Quantum mechanical continuum solvation models. *Chem. Rev.* **2005**, *105*, 2999-3094.
13. Barnsley, J.E.; Scottwell, S.Ø.; Elliott, A.B.S.; Gordon, K.C.; Crowley, J.D. Structural, electronic, and computational studies of heteroleptic Cu(I) complexes of 6,6′-dimesityl-2,2′-bipyridine with ferrocene-appended ethynyl-2,2′-bipyridine ligands. *Inorg. Chem.* **2016**, *55*, 8184-8192.
14. Scottwell, S.O.; Elliott, A.B.S.; Shaffer, K.J.; Nafady, A.; McAdam, C.J.; Gordon, K.C.; Crowley, J.D. Chemically and electrochemically induced expansion and contraction of a ferrocene rotor. *Chem. Commun.* **2015**, *51*, 8161-8164.
15. Scottwell, S.O.; Barnsley, J.E.; McAdam, C.J.; Gordon, K.C.; Crowley, J.D. A ferrocene based switchable molecular folding ruler. *Chem. Commun.* **2017**, *53*, 7628-7631.
16. O'boyle, N.M.; Tenderholt, A.L.; Langner, K.M. Cclib: A library for package-independent computational chemistry algorithms. *J Comput Chem.* **2008**, *29*, 839-845.
17. Earles, J.C.; Gordon, K.C.; Stephenson, A.W.I.; Partridge, A.C.; Officer, D.L. Spectroscopic and computational study of β-ethynylphenylene substituted zinc and free-base porphyrins. *Phys Chem Chem Phys* **2011**, *13*, 1597-1605.
